# Benefits and harms of medical cannabis: a scoping review of systematic reviews

**DOI:** 10.1186/s13643-019-1243-x

**Published:** 2019-12-10

**Authors:** Misty Pratt, Adrienne Stevens, Micere Thuku, Claire Butler, Becky Skidmore, L. Susan Wieland, Mark Clemons, Salmaan Kanji, Brian Hutton

**Affiliations:** 1 Knowledge Synthesis Group, Ottawa Methods Centre, Ottawa Hospital Research Institute, The Ottawa Hospital, General Campus, 501 Smyth Road, Ottawa, Ontario K1H 8 L6 Canada; 2 TRIBE Graduate Program, University of Split School of Medicine, Split, Croatia; 3 Department of Pharmacology and Therapeutics, McGill University, Montreal, Quebec H3A 2B4 Canada; 4 Ottawa, Canada; 5 Center for Integrative Medicine, University of Maryland School of Medicine, Baltimore, MD USA; 6 School of Epidemiology and Public Health, University of Ottawa, 451 Smyth Road, Ottawa, Ontario K1H 8 M5 Canada; 7 Division of Medical Oncology and Department of Medicine, University of Ottawa, Ottawa, Canada; 8 Department of Pharmacy, The Ottawa Hospital, Ottawa, Canada; 9 Clinical Epidemiology Program, The Ottawa Hospital Research Institute, Ottawa, Canada

**Keywords:** Cannabis, Marijuana, Medical marijuana, Scoping review, Systematic review

## Abstract

**Background:**

There has been increased interest in the role of cannabis for treating medical conditions. The availability of different cannabis-based products can make the side effects of exposure unpredictable. We sought to conduct a scoping review of systematic reviews assessing benefits and harms of cannabis-based medicines for any condition.

**Methods:**

A protocol was followed throughout the conduct of this scoping review. A protocol-guided scoping review conduct. Searches of bibliographic databases (e.g., MEDLINE®, Embase, PsycINFO, the Cochrane Library) and gray literature were performed. Two people selected and charted data from systematic reviews. Categorizations emerged during data synthesis. The reporting of results from systematic reviews was performed at a high level appropriate for a scoping review.

**Results:**

After screening 1975 citations, 72 systematic reviews were included. The reviews covered many conditions, the most common being pain management. Several reviews focused on management of pain as a symptom of conditions such as multiple sclerosis (MS), injury, and cancer. After pain, the most common symptoms treated were spasticity in MS, movement disturbances, nausea/vomiting, and mental health symptoms. An assessment of review findings lends to the understanding that, although in a small number of reviews results showed a benefit for reducing pain, the analysis approach and reporting in other reviews was sub-optimal, making it difficult to know how consistent findings are when considering pain in general. Adverse effects were reported in most reviews comparing cannabis with placebo (49/59, 83%) and in 20/24 (83%) of the reviews comparing cannabis to active drugs. Minor adverse effects (e.g., drowsiness, dizziness) were common and reported in over half of the reviews. Serious harms were not as common, but were reported in 21/59 (36%) reviews that reported on adverse effects. Overall, safety data was generally reported study-by-study, with few reviews synthesizing data. Only one review was rated as high quality, while the remaining were rated of moderate (*n* = 36) or low/critically low (*n* = 35) quality.

**Conclusions:**

Results from the included reviews were mixed, with most reporting an inability to draw conclusions due to inconsistent findings and a lack of rigorous evidence. Mild harms were frequently reported, and it is possible the harms of cannabis-based medicines may outweigh benefits.

**Systematic review registration:**

The protocol for this scoping review was posted in the Open Access (https://ruor.uottawa.ca/handle/10393/37247).

## Background

Interest in medical applications of marijuana (*Cannabis sativa*) has increased dramatically during the past 20 years. A 1999 report from the National Academies of Sciences, Engineering, and Medicine supported the use of marijuana in medicine, leading to a number of regulatory medical colleges providing recommendations for its prescription to patients [1]. An updated report in 2017 called for a national research agenda, improvement of research quality, improvement in data collection and surveillance efforts, and strategies for addressing barriers in advancing the cannabis agenda [2].

Proponents of medical cannabis support its use for a highly varied range of medical conditions, most notably in the fields of pain management [3] and multiple sclerosis [4]. Marijuana can be consumed by patients in a variety of ways including smoking, vaporizing, ingesting, or administering sublingually or rectally. The plant consists of more than 100 known cannabinoids, the main ones of relevance to medical applications being tetrahydrocannabinol (THC) and cannabidiol (CBD) [5]. Synthetic forms of marijuana such as dronabinol and nabilone are also available as prescriptions in the USA and Canada [6].

Over the last decade, there has been an increased interest in the use of medical cannabis products in North America. It is estimated that over 3.5 million people in the USA are legally using medical marijuana, and a total of USD$6.7 billion was spent in North America on legal marijuana in 2016 [7]. The number of Canadian residents with prescriptions to purchase medical marijuana from Health Canada–approved growers tripled from 30,537 in 2015 to near 100,000 in 2016 [8]. With the legalization of recreational-use marijuana in parts of the USA and in Canada in October 2018, the number of patients using marijuana for therapeutic purposes may become more difficult to track. The likely increase in the numbers of individuals consuming cannabis also necessitates a greater awareness of its potential benefits and harms.

Plant-based and plant-derived cannabis products are not monitored as more traditional medicines are, thereby increasing the uncertainty regarding its potential health risks to patients [3]. While synthetic forms of cannabis are available by prescription, different cannabis plants and products contain varied concentrations of THC and CBD, making the effects of exposure unpredictable [9]. While short-lasting side effects including drowsiness, loss of short-term memory, and dizziness are relatively well known and may be considered minor, other possible effects (e.g., psychosis, paranoia, anxiety, infection, withdrawal) may be more harmful to patients.

There remains a considerable degree of clinical equipoise as to the benefits and harms of marijuana use for medical purposes [10–13]. To understand the extent of synthesized evidence underlying this issue, we conducted a scoping review [14] of systematic reviews evaluating the benefits and/or harms of cannabis (plant-based, plant-derived, and synthetic forms) for any medical condition. We located and mapped systematic reviews to summarize research that is available for consideration for practice or policy questions in relation to medical marijuana.

## Methods

A scoping review protocol was prepared and posted to the University of Ottawa Health Sciences Library’s online repository (https://ruor.uottawa.ca/handle/10393/37247). We used the PRISMA for Scoping Reviews checklist to guide the reporting of this report (see Additional file 1) [15].

### Literature search and process of study selection

An experienced medical information specialist developed and tested the search strategy using an iterative process in consultation with the review team. Another senior information specialist peer-reviewed the strategy prior to execution using the PRESS Checklist [16]. We searched seven Ovid databases: MEDLINE®, including Epub Ahead of Print and In-Process & Other Non-Indexed Citations, Embase, Allied and Complementary Medicine Database, PsycINFO, the Cochrane Database of Systematic Reviews, the Database of Abstracts of Reviews of Effects, and the Health Technology Assessment Database. The final peer-reviewed search strategy for MEDLINE was translated to the other databases (see Additional file 2). We performed the searches on November 3, 2017.

The search strategy incorporated controlled vocabulary (e.g., “Cannabis,” “Cannabinoids,” “Medical Marijuana”) and keywords (e.g., “marijuana,” “hashish,” “tetrahydrocannabinol”) and applied a broad systematic review filter where applicable. Vocabulary and syntax were adjusted across the databases and where possible animal-only and opinion pieces were removed, from the search results.

Gray literature searching was limited to relevant drug and mental health databases, as well as HTA (Health Technology Assessment) and systematic review databases. Searching was guided by the Canadian Agency for Drugs and Technologies in Health’s (CADTH) checklist for health-related gray literature (see Additional file 3). We performed searches between January and February 2018. Reference lists of overviews were searched for relevant systematic reviews, and we searched for full-text publications of abstracts or protocols.

**Fig. 1:**
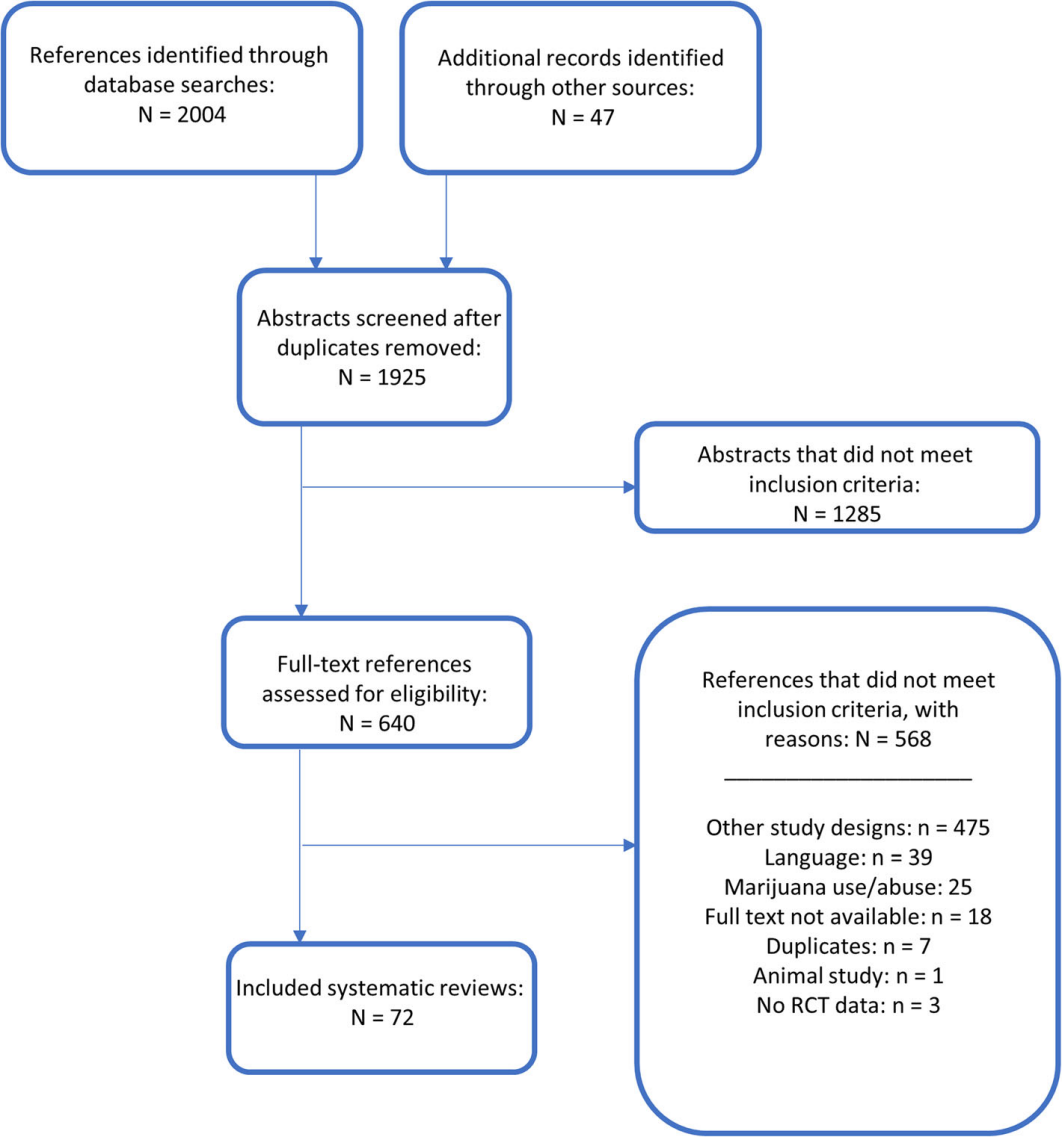
PRISMA-style flow diagram of the review selection process

Management of all screening was performed using Distiller SR Software ® (Evidence Partners Inc., Ottawa, Canada). Citations from the literature search were collated and de-duplicated in Reference Manager (Thomson Reuters: Reference Manager 12 [Computer Program]. New York: Thomson Reuters 2011), and then uploaded to Distiller. The review team used Distiller for Levels 1 (titles and abstracts) and 2 (full-text) screening. Pilot testing of screening questions for both levels were completed prior to implementation. All titles and abstracts were screened in duplicate by two independent reviewers (MT and MP) using the liberal accelerated method [17]. This method requires only one reviewer to assess an abstract as eligible for full-text screening, and requires two reviewers to deem the abstract irrelevant. Two independent reviewers (MT and MP) assessed full-text reports for eligibility. Disagreements during full-text screening were resolved through consensus, or by a third team member (AS). The process of review selection was summarized using a PRISMA flow diagram (Fig. 1) [18].

### Review selection criteria

English-language systematic reviews were included if they reported that they investigated harms and/or benefits of medical or therapeutic use of cannabis for adults and children for any indication. Definitions related to medical cannabis/marijuana are provided in Table 1. We also included synthetic cannabis products, which are prescribed medicines with specified doses of THC and CBD. Reviews of solely observational designs were included only in relation to adverse effects data, in order to focus on the most robust evidence available. We considered studies to be systematic reviews if at least one database was searched with search dates reported, at least one eligibility criterion was reported, the authors had assessed the quality of included studies, and there was a narrative or quantitative synthesis of the evidence. Reviews assessing multiple interventions (both pharmacological and complementary and alternative medicine (CAM) interventions) were included if the data for marijuana studies was reported separately. Published and unpublished guidelines were included if they conducted a systematic review encompassing the criteria listed above.

**Table 1:** Context for the use of cannabis-related terms during the review selection process

Term	Definition
Medical marijuana (or marijuana for medical use)	The term medical marijuana refers to using the whole, unprocessed marijuana plant or its basic extracts to treat symptoms of illness and other conditions (https://www.drugabuse.gov/publications/drugfacts/marijuana-medicine) Whether marijuana is recognized as medicine varies from country to country. The US Food and Drug Administration (FDA) has not recognized or approved the marijuana plant as medicine, but a growing number of states have legalized marijuana for medical use. In Canada, it is legal to possess cannabis for medical purposes, and legalization for non-medical use is set to take place in 2018.
Cannabis for therapeutic purposes (CTP)	A similar term to “medical marijuana,” CTP refers to legal access to cannabis for therapeutic purposes; this includes symptoms associated with health or mental disorders [19]

We excluded overviews of systematic reviews, reviews in abstract form only, and review protocols. We further excluded systematic reviews focusing on recreational, accidental, acute, or general cannabis use/abuse and interventions such as synthetic cannabinoids not approved for therapeutic use (e.g., K2 or Spice).

### Data collection and quality assessment

All data were collected electronically in a pre-developed form using Microsoft Excel software (Microsoft Corporation, Seattle, USA). The form was pilot tested on three included reviews by three people. One reviewer (MP or CB) independently extracted all data, and a second reviewer (MT) verified all of the items collected and checked for any omitted data. Disagreements were resolved by consensus and consultation with a third reviewer if necessary. A data extraction form with the list of included variables is provided in Additional file 4. All collected data has also been made available in the online supplemental materials associated with this report.

Quality assessment of systematic reviews was performed using the AMSTAR-2 [20] tool. One reviewer (MP or CB) independently assessed quality, while a second reviewer (MT) verified the assessments. Disagreements were resolved by consensus and consultation with a third reviewer if necessary. The tool consists of 16 items in total, with four critical domains and 12 non-critical domains. The AMSTAR-2 tool is not intended to generate an overall score, and instead allows for an overall rating based on weaknesses in critical domains. Reviews were rated as high (no critical flaws with zero or one non-critical flaw), moderate (no critical flaws with ≥ 1 non-critical flaw), low (one critical flaw with/without non-critical weakness), or critically low (> 1 critical flaw with/without non-critical weakness) quality.

### Evidence synthesis

We used a directed content analytic approach [21] with an initial deductive framework [22] that allowed flexibility for inductive analysis if refinement or development of new categorization was needed. The framework used to categorize outcome data results is outlined in Table 2. Where reviews had a mix of narrative and quantitative data, results from meta-analyses were prioritized over count data or study-by-study data. The extraction and reporting of data results was performed at a high level and did not involve an in-depth evaluation, which is appropriate for a scoping review [14]. Review authors’ conclusions and/or recommendations were extracted and reported narratively.

**Table 2:** Outcome result categorization

Outcome data categorization	Definition
Favors intervention	Review authors conducted a meta-analysis and/or narrative synthesis (i.e., count data) which shows a beneficial effect for cannabis.
Favors control	Review authors conducted a meta-analysis and/or narrative synthesis (i.e., count data) which shows a beneficial effect of control.
Unclear efficacy/insufficient data	Review authors do not provide enough information to make a clear conclusion, or state that data from included studies is insufficient.
No statistically significant difference between groups	Review authors conducted a meta-analysis and/or narrative synthesis (i.e., count data) which shows no significant difference between cannabis and control groups (precision with confidence interval [included null results] preferred over *p* value).
Reported study-by-study (SBS)	Authors narratively review the primary study data, without providing an overall count of positive, negative, or no difference effects. Included studies are reviewed individually, with or without author’s final recommendations and conclusions

### Changes from the study protocol

For feasibility, we decided to limit the inclusion of systematic reviews of only observational study designs to those that addressed adverse events data. All other steps of the review were performed as planned.

## Results

### Search findings

The PRISMA flow diagram describing the process of review selection is presented in Fig. 1. After duplicates were removed, the search identified a total of 1925 titles and abstracts, of which 47 references were located through the gray literature search. Of the total 1925 citations assessed during Level 1 screening, 1285 were deemed irrelevant. We reviewed full-text reports for the 640 reviews of potential relevance, and of these, 567 were subsequently excluded, leaving a total of 72 systematic reviews that were included; the associated data collected are provided in Additional file 5. A listing of the reports excluded during full-text review is provided in Additional file 6.

### Characteristics of included reviews

There were 63 systematic reviews [4, 19, 23–83] and nine guidelines with systematic reviews [84–92]. Overall, 27 reviews were performed by researchers in Europe, 16 in the USA, 15 in Canada, eight in Australia, two in Brazil, and one each in Israel, Singapore, South Africa, and China. Funding was not reported in 29 (40%) of the reviews, and the remaining reviews received funding from non-profit or academic (*n* = 20; 28%), government (*n* = 14; 19%), industry (*n* = 3; 4%), and mixed (*n* = 1; 1%) sources. Five reviews reported that they did not receive any funding for the systematic review. Tables 3, 4, 5, 6, 7, 8, 9, 10, 11, 12, and 13 provide an overview of the characteristics of the 72 included systematic reviews.

**Table 3:** Multiple sclerosis

Author, year	Search dates; no. databases searched	Funding source	*N*_studies_	Illness/condition	Intervention/comparator*	Outcomes	Reported results	AMSTAR-2 rating
Herzog, 2017 [4]	Inception–mid-Dec 2016; 8	No funding	10	Chronic illness (all included studies on MS)	I: Plant-derived cannabinoids C: Standard care (anti-spasticity drugs)	• Maintenance of treatment gains • QoL	Reported SBS	L
Claflin, 2017 [23]	January 17, 2018; 1	NR	7	MS	I: Plant-derived cannabinoids C: Placebo	• Pain • Incontinence • Spasticity • Muscle stiffness • VAS of most troublesome symptoms	Favors intervention for pain, incontinence, spasticity, and muscle stiffness; reported SBS for troublesome symptoms	L
Behm, 2017 [24]	Until 30 Nov 2017; 5	NR	4	MS	I: Plant-derived and synthetic cannabinoids C: Placebo; cannabis extract	• Gait speed	Reported SBS	L
Youssef, 2017 [34]	1 Jan 1946–11 Nov 2006; 4	Non-profit	3	MS	I: Plant-derived cannabinoids C: Placebo	• Decrease in incontinence episodes • Decrease in number nocturia episodes • Daytime voids • Voids per 24 h • Urgency episodes/d • Withdrawal due to AEs	Favors intervention for decrease in incontinence episodes; reported SBS for nocturia episodes, daytime voids, voids per 24 h, urgency episodes, and withdrawal due to AEs	L
Yadav, 2014 [92]	First search: 1970–Mar 2011; second search (Medline only): Mar 2011 to Sept 2013; 5	Non-profit	19	MS	I: All types of cannabinoids C: Placebo	• Pain • Central NP • Spasticity • Tremor • Bladder symptoms • Balance • Posture • Cognition • Total/average AEs	Reported SBS	M
Lakhan, 2009 [56]	1999–Apr 2009; 3	NR	6	MS	I: Plant-derived cannabinoids C: Placebo; THC	• Decreased spasticity • Mobility • Efficacy • Ashworth score • Walk time • Spasticity (VAS scores) • RMI score • Spasticity (subjective)	Favors intervention for decreased spasticity, mobility, spasticity (VAS scores), and subjective spasticity. No statistically significant difference between groups for efficacy, Ashworth score, and RMI score. Unclear efficacy for walk time.	M
Mills, 2007 [63]	MEDLINE 1966, EMBASE 1988, and Cochrane to Jun 2006; 4	NR	3	MS	I: Plant-derived and synthetic cannabinoids C: Placebo	• Tremor • Pain (VAS) • Arm and hand function • Ataxia • Disability outcomes	No statistically significant difference between groups for tremor. Reported SBS for all other outcomes	L
Shakespeare, 2003 [64]	MEDLINE 1966, EMBASE 1988 and Cochrane to Jun 2003; 4	NR	2	MS	I: Plant-derived cannabinoids C: Placebo	• Ashworth score • Brainstem functioning • MS functional composite score • Subjective global rating • Spasms and spasticity • Spasticity (NRS) • Spasm frequency	No statistically significant difference between groups for Ashworth score. Only one study included for all other outcomes	CL
Kuspinar, 2012 [71]	Start search date varies by database to Sept 2011; 4	Non-profit	1	MS	I: Plant-derived cannabinoids C: Placebo	• Number of incontinence episodes • QoL (incontinence questionnaire)	Reported SBS	L
NICE, 2014 [91]	Search updated on Feb 3, 2014; 6	Government	6	MS	I: Plant-derived cannabinoids C: Placebo	• Spasticity (Ashworth score) • Spasticity (NRS score) • Activities of daily living • Spasm severity (NRS) • Spasm severity (30% improvement in NRS) • Timed 10 min walk • Global impression of improvement • Motricity • QoL • Guys neurological disability scale • Adverse events	Favors intervention for spasticity (NRS score), spasm severity (30% improvement in NRS), and global impression of improvement. No statistically significant difference between groups for spasticity (Ashworth score), spasm severity (NRS), motricity, QoL, and most adverse effects. Favors control for activities of daily living and Guys neurological disability scale	M
da Rovare, 2017 [27]	Up to Mar 20, 2017; 4	NR	16 (24 pubs)	MS or paraplegia	I: Cannabinoids (not specified) C: placebo	• Spasticity • Spasm • Cognitive function • Daily activities • Motricity • Pain • Bladder function • Dizziness • Somnolence • Headache • Nausea • Dry mouth	No statistically significant differences between groups for spasticity, spasm, pain, cognitive function, daily activities, motricity, and bladder function. Favors placebo for dizziness, somnolence, nausea, and dry mouth	M

*MS* multiple sclerosis, *NICE* National Institute for Health and Care Excellence, *No*. number, *NR* not reported, *NRS* numerical rating scale, *QoL* quality of life, *RMI* Rivermead Mobility Index, *SBS* study-by-study, *VAS* visual analog scale

*A colon indicates that there were separate analyses for each comparator

**Table 4:** Movement disorders

Author, year	Search dates; # databases searched	Funding source	*N*_studies_	Illness/condition	Intervention/comparator	Outcomes	Conclusions from data	AMSTAR-2 rating
Koppel, 2014 [85]	Inception–Nov 2013; 5	Non-profit	34	MS, HD, PD, cervical dystonia, Tourette syndrome, epilepsy	I: All types of cannabinoids C: Placebo	• Spasticity • Central NP • Bladder symptoms • Tremor • Symptomatic HD treatment • Levodopa-induced dyskinesias • Tic severity • Cervical dystonia • Seizure frequency • Discontinuation of medication due to AEs	Reported SBS for all outcomes except discontinuation of medication due to AEs (favors placebo)	L
Mestre, 2009 [75]	Start search date varies by database to Dec 2007; 4	Non-profit	1	HD	I: Plant-derived cannabinoids C: placebo	• Chorea severity • Functional capacity • HD staging system • Motor function (SCL-90R) • Cognition (SCL-90R) • Psychological distress (SCL-90R)	Only one study included	M
Chung, 2006 [78]	Searched in Jul 2005; 11	Non-profit	1	PD	I: Plant-based cannabis C: placebo	• Therapeutic effect on L-dopa induced dyskinesia • Unified PD rating scale • QoL • Total AEs	Only one study included	L
Pringsheim, 2012 [86]	MEDLINE 1950 and EMBASE 1980 to Oct 2010; 2	Government	2	Tic disorders	I: Plant-derived cannabinoids C: Placebo	• Tic frequency and severity • Total AEs	Favors intervention	L
Curtis, 2009 [57]	Start search date varies by database and runs “to date”; 8	Government	2	Tourette syndrome	I: Plant-derived cannabinoids C: Placebo	• Tic reduction • Tic severity • STSSS • YGTSS • TSSL • Obsessive Compulsive disorder (TSSL) • TS–CGI • Video rating • Total AEs	Favors intervention for tic reduction; reported SBS for total AEs; only one study included for all other outcomes	M

*HD* Huntington’s disease, *MS* multiple sclerosis, *NR* not reported, *PD* Parkinson’s disease, *SBS* study-by-study, *SCL-90R* Symptoms Checklist-90 Revised, *QoL* quality of life, *STSSS* Shapiro Tourette Syndrome Severity Scale, *THC* tetrahydrocannabinol, *TS-CGI* Tourette Syndrome Clinical Global Impressions, *TSSL* Tourette’s Syndrome Symptom List (patient rated), *VAS* visual analog scale, *YGTSS* Yale Global Tic Severity Scale

**Table 5:** Pain

Author, year	Search dates; # databases searched	Funding source	*N*_studies_	Illness/condition	Intervention/comparator*	Outcomes	Conclusions from data	AMSTAR-2 rating
Chronic pain, any kind								
Martin-Sanchez, 2009 [58]	To Feb 2008; 3	Government	18	Chronic pain	I: Plant-derived and synthetic cannabinoids C: Placebo	• Pain • Euphoria • Dysphoria • Events linked to alterations in perception • Events affecting motor function • Events that altered cognitive function	Favors intervention for pain, euphoria, events linked to alternations in perception, events affecting motor function, and events that altered cognitive function. No statistically significant difference between groups for dysphoria.	M
Nielsen, 2017 [80]	No date limits; search run on Oct 29, 2015; 4	Government	9	Chronic pain	I: Plant-derived or synthetic cannabinoids alone or with opioids C: Placebo + opioids	• Analgesia • Pain intensity • Experimental pain • Opioid-sparing effect • Sleep • Energy • Social functioning	Favors intervention for analgesia. Favors control for opioid-sparing effect, but analysis of high-quality studies for this outcome shows unclear efficacy. Only one study included for all other outcomes	M
Nugent, 2017 [32]	Inception–Mar 2017; 5	Government	75	Chronic pain in various conditions	I: Plant-derived cannabinoids C: Placebo or NR	• Central NP • NP • Cancer pain • Risk of short-term non-serious AEs • Lung function • Pulmonary effects • Cardiovascular events	No statistically significant difference for NP in MS or cancer pain; favors intervention for NP in other conditions; reported SBS for risk of short-term AEs, lung function, pulmonary effects, cardiovascular events	M
Deshpande, 2015 [41]	Searched in April 2014; 3	NR	6	Chronic non-cancer pain	I: Plant-derived cannabinoids C: placebo	• Pain relief • Pain reduction • Effect on dose of other analgesics • QoL	Favors intervention for NP; only one study included on dose of other analgesics; no statistically significant difference between groups for QoL	L
Lynch, 2015 [68]	2010–Oct 2014; 10	NR	11	Chronic non-cancer pain: FM, medication overuse, MS, OA, diabetic and chemotherapy-induced neuropathy	I: All types of cannabinoids C: Unclear for one analysis (placebo, ibuprofen, o ramitriptyline); placebo; ibuprofen; amitriptyline	• Analgesia • Pain intensity • Pain • Analgesic intake and dependence • Sleep • Anxiety • Sleep quality • VAS and patient global assessment of change • NP • Muscle stiffness pain • Serious AEs	Favors intervention for analgesia (cannabinoids vs. placebo.) Reported SBS for serious AEs. Only one study included for all other outcomes	L
Aviram, 2017 [26]	1975–Jul 2015; 2	No funding	43	Chronic or postoperative pain	I: Plant-derived and synthetic cannabinoids C: placebo	• NP • Peripheral NP • Chronic pain • Postoperative pain • Acute postoperative pain	Favors intervention for all types of pain except acute postoperative pain (favors control)	M
Meng, 2017 [31]	To Mar 11, 2016; 6	Non-profit	11	Chronic NP	I: Plant-derived and synthetic cannabinoids C: Placebo; dihydrocodeine	• NP • Central NP • Peripheral NP • QoL • Anxiety • Satisfaction of participants • QST profile • Withdrawal due to AEs	Favors intervention for NP, QoL, satisfaction, and QST profile; reported SBS for central NP and withdrawal due to AEs (vs. placebo); no statistically significant difference between groups for peripheral NP (vs. placebo.) Reported SBS for mixed central and peripheral NP (vs. dihydrocodeine)	M
Andreae, 2015 [42]	Searched Apr 23, 2014;4	Mixed	5	Chronic NP	I: Plant-based cannabis C: placebo	• Peripheral NP • Withdrawal due to AEs	Favors intervention for peripheral NP. Reported SBS for withdrawal due to AEs.	M
Pain								
Campbell, 2001 [65]	MEDLINE 1966, EMBASE 1974, and Cochrane to Oct 1999; Oxford pain database: 1950–1994; 4	NR	9	Pain	I: All types of cannabinoids C: Placebo; codeine	• Nociceptive pain • Postoperative pain • Cancer pain • Abdominal pain • NP • Spasticity • Subjective improvement of MS symptoms • Balance • Withdrawal due to AEs	Favors intervention for nociceptive pain and postoperative pain (cannabinoids vs. placebo). No statistically significant difference between groups for nociceptive pain, postoperative pain, and cancer pain (cannabinoids vs. codeine). Reported SBS for withdrawal due to AEs (compared with both placebo and codeine.) Only one study included for all other outcomes	M
Finnerup, 2015 [69]	Jan 1966–to Jan 31 2014; 5	Non-profit	9	Pain in MS, diabetes, allodynia, SCI,	I: Plant-derived cannabinoids C: placebo	• NP (NNT for 30–50% pain reduction)	No statistically significant difference between groups	M
Iskedjian, 2007 [62]	Inception to end of June 2006; 4	Industry	7	NP	I: Plant-derived cannabinoids C: Placebo	• Pain • Withdrawals due to AEs • Dizziness • Somnolence • Headache • Nausea • Diarrhea • Fatigue	Favors intervention for pain; unclear/indeterminate for all adverse effects	CL
NICE, 2013 [90]	Searches conducted between Jul 17 and 31st and Aug 23rd and 29th of August; 10	NR	4	NP	I: Plant-derived cannabinoids C: Placebo; “other drugs”; amitriptyline; pregabalin	• Pain • Continuous pain • Burning pain • Patient reported global improvement • Sleep • Withdrawal due to adverse effects • Dizziness or vertigo • Drowsiness • Fatigue • Nausea • Vomiting • Burning pain • Cognitive impairment • Mood disturbance • Dry mouth	Favors intervention for pain, continuous pain, withdrawal due to adverse effects, sleep, dizziness, or vertigo (cannabinoids vs. placebo). Favors control for pain (cannabinoids vs. other drugs). No statistically significant difference between groups for withdrawal due to adverse effects and dizziness or vertigo (cannabinoids vs. amitriptyline), drowsiness, fatigue, nausea, vomiting, burning pain, cognitive impairment, mood disturbance, dry mouth, 30% pain relief, global improvement. Reported SBS for sleep (vs. placebo or pregabalin).	M
Acute pain								
Stevens, 2017 [38]	To Aug 20, 2016; 3	No funding	7	Acute pain	I: Cannabinoids (not specified) C: Placebo	• Acute pain • Total/average AEs • Withdrawal due to AEs	No statistically significant difference between groups; AEs reported SBS	M

*AE*: adverse effect, *NICE* National Institute for Health and Care Excellence, *NNT* numbers needed to treat, *NP* neuropathic pain, *NR* not reported, *QoL* quality of life, *QST* quantitative sensory testing, *SBS* study-by-study, *VAS* visual analog scale

*A colon indicates that there were separate analyses for each comparator; a “+” sign indicates placebo was combined with another comparator

**Table 6:** Cancer

Author, year	Search dates; # databases searched	Funding source	N_studies_	Illness/condition	Intervention/comparator*	Outcomes	Conclusions from data	AMSTAR-2 rating
van den Beuken-van Everdingen, 2017 [84]	Jan 2005–May 2014; 3	NR	3	Cancer	I: Plant-derived cannabinoids C: placebo; THC alone	• Cancer pain: nociceptive pain, NP, and chemotherapy-induced pain • Rate of adverse events	Only one study included	CL
Tateo, 2017 [37]	NR; 4	NR	8	Cancer	I: Plant-based and plant-derived cannabinoids C: Placebo; codeine; secobarbital; THC	• Pain • Sleep disruption • QoL • Impression of global change • Sensory function • Withdrawal due to AEs	Favors intervention for pain with nabiximols (vs. placebo); reported SBS for all other interventions and outcomes.	M
Smith, 2015 [40]	Inception–Jan 2015; 5	Non-profit	23	Cancer	I: Synthetic cannabinoids C: Placebo; prochlorperazine; domperidone; metoclopramide	• Absence of nausea • Absence of vomiting • Absence of both nausea and vomiting • participant preference • Dysphoria • Feeling high • Sedation • Withdrawal due to adverse events • Dizziness • Euphoria • Hallucinations • Postural hypotension • Depression • Withdrawal due to lack of efficacy	No statistically significant differences between groups for: absence of nausea, dysphoria, sedation, hallucinations, hypotension, depression, withdrawal due to lack of efficacy. Favors intervention for absence of vomiting, absence of nausea and vomiting, feeling high, withdrawal due to adverse events, dizziness, dysphoria, euphoria, patient preference Note: mixed results based on several subgroup analyses	M
Phillips, 2010 [54]	Inception to Feb or Mar 2008; 11	Non-profit	4	Cancer	I: Plant-derived and synthetic cannabinoids C: Prochlorperazine and metoclopramide; domperidone; prochlorperazine	• Nausea and vomiting	Only one study included	M
Machado Rocha, 2008 [60]	Inception to Dec 2006; 5	NR	30	Cancer	I: Synthetic cannabinoids C: Placebo; neuroleptic drugs	• Anti-emetic efficacy • Preference for drug	No statistically significant difference for anti-emetic effect of dronabinol vs. placebo, and nabilone or levonantradol vs. neuroleptics. Favors intervention for anti-emetic effect of dronabinol vs. neuroleptic and preference of drug.	M
Yavuzsen, 2005 [79]	Start search date varies by database to Oct 2004	NR	1	Cancer	I: Plant-based cannabis alone or in combination with megestrol acetate C: Megestrol acetate	• Weight • Appetite • QoL	Only one study included	CL
The Belgian Health Care Knowledge Centre (KCE), 2012 [89]	Dec 2011 to Aug 2012; 6	NR	4	Chemotherapy-related adverse events	I: Plant-based cannabis and synthetic cannabinoids + chemo C: Placebo (+ chemo)	• Complete response to anti-emetic therapy • Absence of delayed nausea • Significant delayed nausea • Absence of delayed emesis • QoL • Nausea absence • Vomiting and/or retching (mean number of episodes per week) • Patient’s wellness • At least one AE • Severe AEs • At least one treatment emerging AE • At least one serious AE	Only one study narratively described for each outcome, except for AEs. No statistically significant difference between groups for at least one AE and severe AEs (cannabis vs. placebo.) Favors dronabinol for at least one treatment emerging AE. Favors placebo for at least one serious AE.	M
American Society of Clinical Oncology, 2016 [88]	Searched on Nov 5, 2014; 1	Non-profit	4	Cancer	I: Plant-derived cannabinoids C: Placebo	• Pain (NRS score) • Worsening of nausea and vomiting	Only one study included	L
SIGN, 2008 [87]	1997 to Jun 2007; 8	Government	3	Cancer	I: Plant-based cannabis and plant-derived cannabinoids C: Placebo	• NP • Central NP	Favors intervention for NP (types of cannabis combined). Only one study included for NP (smoked cannabis) and central NP	M

*AE* adverse effect, *NP* neuropathic pain, *NR* not reported, *NRS* numerical rating scale, *QoL* quality of life, *THC* tetrahydrocannabinol, *SIGN* Scottish Intercollegiate Guidelines Network, *SBS* study-by-study

*A colon indicates that there were separate analyses for each comparator; a “+” sign indicates placebo was combined with another comparator

**Table 7:** Rheumatic disease

Author, year	Search dates; # databases searched	Funding source	*N*_studies_	Illness/condition	Intervention/comparator*	Outcomes	Conclusions from data	AMSTAR-2 rating
Walitt, 2016 [39]	To Apr 26, 2016; 3	No funding	2	Fibromyalgia	I: Synthetic cannabinoids C: Placebo; Amitriptyline	• Pain • Anxiety • QoL • Fatigue • Depression • Insomnia • Mood states • Withdrawal due to AEs	Reported SBS for all outcomes (vs. placebo or amitriptyline)	M
Fitzcharles, 2016 [49]	Sept 2013 (updated Jan 2015); 11	NR	4	Rheumatic diseases (inflammatory arthritis, OA, soft tissue rheumatism, and FM)	I: Plant-derived and synthetic cannabinoids C: Placebo; amitriptyline	• Pain • Sleep quality • Disease activity score • QoL • Sleep measures • Withdrawal due to AEs • Total AEs	Reported SBS for pain, sleep, disease activity, and withdrawal due to adverse events (vs. placebo); reported SBS for QoL, sleep measures, withdrawal due to adverse events, and total adverse events (vs. amitriptyline)	M
Richards, 2012 [51]	Inception to Nov 2010; 4	Non-profit	1	RA	I: Plant-derived cannabinoids C: Placebo	• Movement and morning pain • Total intensity of pain • Pain at present • Sleep • Withdrawal due to adverse events • Total adverse events	Favors intervention for movement and morning pain, sleep NRS, and total adverse events. No statistically significant difference between groups for total intensity of pain, pain at present, and withdrawal due to adverse events	M
Macfarlane, 2011 [73]	Start search date varies by database to Aug 2010; 7	Non-profit	1	RA	I: Plant-derived cannabinoids C: Placebo	• Pain • Quality of sleep • 28-joint disease activity score	Only one study included	L

*AE* adverse event, *FM* fibromyalgia, *NR* not reported, *NRS* numerical rating scale, *OA* osteoarthritis, *RA* rheumatoid arthritis, *SBS* study-by-study

*A colon indicates that there were separate analyses for each comparator

**Table 8:** Injury

Author, year {refid}	Search dates; # databases searched	Funding source	*N*_studies_	Illness/condition	Intervention/comparator	Outcomes	Conclusions from data	AMSTAR-2 rating
Snedecor, 2013 [29]	To Dec 2011; 5	Industry	1	NP associated with spinal cord injury	I: Synthetic cannabinoids C: Placebo	• NP • All-cause discontinuation	Favors control for NP; no statistically significant different between groups for all-cause discontinuation	CL
Mehta, 2016 [35]	2009–Sept 2015; 4	NR	2	Spinal cord injury	I: Plant-derived and Synthetic cannabinoids C: diphenhydramine	• NP • Spastic pain	Reported SBS	L
Meyer, 2010 [55]	1980–2008; 4	NR	2	Acquired brain injury	I: Synthetic cannabinoids C: Placebo	• Intercranial pressure • Glasgow outcome scale • Disability rating scale • Mortality rates • Activities of daily living • QoL	Reported SBS	M
Wheaton, 2009 [76]	Jan 1980 to May 2008; 2	Non-profit	2	Traumatic brain injury	I: Synthetic cannabinoids C: placebo	• Global outcome score (3 and 6 months)	No statistically significant difference between groups	CL

*NP* neuropathic pain, *NR* not reported, *QoL* quality of life, *SBS* study-by-study

**Table 9:** Mental health

Author, year {refid}	Search dates; # databases searched	Funding source	*N*_studies_	Illness/condition	Intervention/comparator	Outcomes	Conclusions from data	AMSTAR-2 rating
Walsh, 2017 [19]	1960–Sept 2015; 2	Government	31	Mental health	I: Plant-derived cannabinoids C: Cannabinoid with no THC	• Improvement in anxiety and depression	Only one RCT included (combined with cross-sectional data)	CL
O’Neil, 2017 [33]	Inception–Mar 2017; 6	Government	5	PTSD	I: All types of cannabinoids C: Different dose or different duration of dose of cannabinoids	N/A observational data	N/A observational data	M
McLoughlin, 2014 [45]	To Aug 12, 2013; 6	Non-profit	1	Schizophrenia	I: Plant-derived cannabinoids C: Amisulpride	• Mental state outcomes	Only one study included	H

*PTSD* posttraumatic stress disorder, *SBS* study-by-study

**Table 10:** HIV/AIDS

Author, year {refid}	Search dates; # databases searched	Funding source	*N*_studies_	Illness/condition	Intervention/comparator	Outcomes	Conclusions from data	AMSTAR-2 rating
Lutge, 2013 [48]	1980–Jul 2012; 7	NR	7	HIV/AIDS	I: Plant-based and synthetic cannabinoids C: Placebo	• Change in body fat • Appetite • Food/caloric intake • Nausea and vomiting • Performance • Mood • Subjective experience of drug • Peripheral NP • Effect on pharmacokinetics of protease inhibitors • Viral load and CD4 count • Resting heart rate • Skin temperature • Withdrawal due to adverse events	Reported SBS for all outcomes except change in mood, which found no statistically significant difference between groups	M
Phillips, 2010 [53]	Search on Jun 20, 2008 and updated Feb 22, 2010; 4	Non-profit	2	HIV	I: Plant-based cannabis C: placebo	• NP	Favors intervention	CL
Merlin, 2016 [67]	Inception to Jan 2015; CENTRAL: Jun 2014; 5	Government	1	HIV	I: Plant-based cannabis C: Placebo	• NP	Reported SBS	CL

*NP* neuropathic pain, *NR* not reported, *SBS* study-by-study

**Table 11:** Neurological conditions

Author, year {refid}	Search dates; # databases searched	Funding source	*N*_studies_	Illness/condition	Intervention/comparator	Outcomes	Conclusions from data	AMSTAR-2 rating
Baldinger, 2012 [50]	To Jan 2011 and Cochrane specialized register to Feb 14, 2011; 4	Non-profit	1	ALS/motor neuron disease	I: Plant-derived cannabinoids C: Placebo	• Muscle cramps • Muscle cramps as AEs	No statistically significant difference between groups	L
Gloss, 2014 [72]	Searched on Sept 9, 2013; 6	NR	4	Epilepsy	I: Plant-derived cannabinoids C: placebo	• Seizure freedom for 12 months	No studies assessed primary outcome in this review	M
Krishnan, 2009 [59]	Dec 2005–Apr 2008; 6	NR	1	Dementia	I: Synthetic cannabinoids C: Placebo	• Body weight • Triceps skinfold thickness • Disturbed behavior • Affect	Only one study included	L
Hanson, 2011 [74]	Jan 1990 to Oct 2009; 5	Government	1	Dementia	I: Synthetic cannabinoids C: placebo	• Weight • Negative affect • Disruptive behavior	Only one study included	L
CADTH, 2018 [83]	Jan 1 2012 to Nov 29, 2017; 6	No funding	4	Dementia	I: Plant-derived cannabinoids C: Placebo	• Static balance • Dynamic balance • Stride length • Total AEs • Dizziness • Somnolence • Balance disorders • Falls • Severe AEs	Only one study included	CL

*AE* adverse effect, *ALS* amyotrophic lateral sclerosis, *CADTH* Canadian Agency for Drugs and Technologies in Health, *NR* not reported

**Table 12:** Various conditions

Author, year	Search dates; # databases searched	Funding source	*N*_studies_	Illness/condition	Intervention/comparator*	Outcomes	Conclusions from data	AMSTAR-2 rating
Kim, 2017^97^	Inception–apr 2017; 3	Non-profit	24	Dystonia, HD, PD, Tourette syndrome, AD, dementia, ALS, psychosis, schizophrenia, anxiety	I: Plant-derived and synthetic cannabinoids C: Placebo; diazepam; amisulpride	• Weight gain • Anti-anxiety • CGI-C • Clinical improvements • Disturbed behavior • Sleep outcomes • Chorea outcomes • Dyskinesia • Motor symptoms • QoL • Tics • OCD behavior • Withdrawal due to adverse effects	Favors intervention for anti-anxiety effects; reported SBS for all other outcomes (vs. diazepam, placebo or amisulpride)	M
Goldenberg, 2017 [30]	To 2015; 4	No funding	20	Fibromyalgia, HIV, IBD, pain, MS, headache, cramps, cancer-related anorexia, traumatic brain injury	I: Plant-based and plant-derived cannabinoids C: Various combined (non-users, placebo, ibuprofen)	• QoL	No statistically significant difference between groups	L
Fitzcharles, 2016 [36]	To Apr 30, 2015; 2	NR	4	Various conditions (chronic spinal pain, rheumatoid arthritis, osteoarthritis, or fibromyalgia)	I: All types of cannabinoids C: Placebo or amitriptyline	• Pain • Anxiety • QoL • Fatigue • Depression • Withdrawal due to AEs • Total AEs	Favors intervention for pain (vs. placebo); no statistically significant difference between groups for anxiety, QoL, fatigue, and depression; reported SBS for withdrawal due to AEs and total AEs. Only a single study included comparing cannabinoids to amitriptyline.	M
Whiting, 2015 [43]	Inception to between Apr 2014 and Apr 2015; 8	Government	79	Various conditions: cancer (chemo-induced nausea and vomiting), appetite stimulation for HIV/AIDS, chronic pain, spasticity in MS or paraplegia, depression, anxiety disorder, sleep disorder, psychosis, intraocular pressure in glaucoma, Tourette syndrome	I: All types of cannabinoids C: Placebo	• NP • Cancer pain • Nausea and vomiting • QoL • Spasticity • Walking speed • Activities of daily living • CGI-C • Spasticity (various measures) • Sleep outcomes • Any AEs • Serious AEs • Withdrawal due to AEs	Mixed results based on how pain is measured (3 MAs favor intervention for NRS scores, patients CGI-C, and NP Scale and 2 show no statistically significant difference between groups for pain reduction ≥ 30% NRS or VAS and BPI-S); favors intervention for nausea and vomiting, spasticity (NRS or VAS), sleep quality, and CGI-C; no statistically significant difference between groups for QoL, 30% or 50% reduction in spasticity NRS, Ashworth score, ADL, sleep disturbance, any AEs, serious AEs, and withdrawal due to AEs	M
Gates, 2014 [46]	NR; 8	NR	28	Various (pain, MS, anorexia, cancer, immune deficiency)	I: Cannabinoids (not specified) C: NR; experimental drugs	• Impact on sleep • Subjective measures of sleep • Objective measures of sleep • Effect of dose on sleep	Favors intervention for impact on sleep, insufficient evidence for subjective measure of sleep (vs. experimental drugs), reported SBS for objective measures of sleep, and effect of dose on sleep	L
van den Elsen, 2014 [47]	To Oct 7, 2013; 4	Government	5	AD, PD, chemotherapy-induced nausea, and vomiting, COPD	I: Plant-based and synthetic cannabinoids C: Placebo; Prochlorperazine	• Dyskinesia • Breathlessness • Chemotherapy-induced nausea and vomiting • Behavioral problems • Weight gain • Triceps skinfold thickness	Reported SBS	M
Lynch, 2011 [52]	Search run between Sept 7 and Oct 7 2010 and not limited by date; 11	NR	18	Chronic pain, fibromyalgia, HIV, MS, rheumatoid arthritis, brachial plexus avulsion, spinal cord or brachial plexus injury, limb amputation	I: All types of cannabinoids C: Placebo; dihydrocodeine	• Central NP • Central pain • NP • Analgesia • Spasticity-related pain • FM pain • Allodynia • Hyperalgesia • Sensory Neuropathy • Spinal pain • Sleep • RA Disease Activity • Activities of daily living • FIQ	Favors intervention for all types of pain combined and sleep. Favors control for activities of daily living. Reported SBS for NP (vs. dihydrocodeine)	L
Wang, 2008 [61]	MEDLINE: Jan 1966 to week 5 of Oct 2007; PsycINFO: Jan 1967 to week 5 of Oct 2007; and EMBASE: Jan 1980 to week 42 of 2007; 3	Government	31	Various (looking at adverse events)	I: Plant-derived cannabinoids C: Placebo; standard care	• Serious adverse events • Death rate • Rate of non-serious adverse events • Average rate of non-serious adverse events	Favors intervention for rate and average rate of non-serious adverse events, except in the case of THC:CBD vs. standard care (no significant difference between groups). No statistically significant difference between groups for serious adverse events and death rate.	L
CADTH, 2011 [6]	Jan 1 2010 to Sept 18, 2015; 4	NR	5	PTSD, FM, chronic pain, spasticity-related pain, MS, peripheral NP, SCI	I: Synthetic cannabinoids C: Placebo, placebo + gabapentin	• Recurring/distressing dreams—PTSD scale • General wellbeing questionnaire • CGI-C • Pain • Peripheral NP • Quality of sleep • Spasticity	Only one study included	L

*AE* adverse effect, *AD* Alzheimer’s disease, *ALS* amyotrophic lateral sclerosis, *CADTH* Canadian Agency for Drugs and Technologies in Health, *CGI-C* Clinical Global Impression of Change scale, *COPD* Chronic Obstructive Pulmonary Disease, *FIQ* fibromyalgia impact questionnaire, *FM* fibromyalgia, *HD* Huntington’s disease, *IBD* inflammatory bowel disease, *MS* multiple sclerosis, *NP* neuropathic pain, *NR* not reported, *PD* Parkinson’s disease, *PTSD* posttraumatic stress disorder, *RA* rheumatoid arthritis, *SBS* study-by-study, *SCI* spinal cord injury

*A colon indicates that there were separate analyses for each comparator; a “+” sign indicates placebo was combined with another comparator

**Table 13:** Other conditions

Author, year {refid}	Search dates; # databases searched	Funding source	*N*_studies_	Illness/condition	Intervention/comparator*	Outcomes	Conclusions from data	AMSTAR-2 rating
Huntley, 2000 [66]	Inception to Dec 1999; 4	NR	1	Asthma	I: Plant-derived cannabinoids C: Placebo	• Airway resistance (raw)	Only one study included	M
Singh, 2007 [77]	Inception to Dec 2004; 6	Non-profit	4	Asthma	I: Plant-based cannabis C: Placebo; isoproterenol; low-dose marijuana	• Pulmonary function test	Reported SBS	M
Snedecor, 2013 [70]	To Jun 2011; 5	Industry	1	Diabetes	I: Plant-derived cannabinoids C: placebo	• Peripheral NP (mean reduction in pain) • Peripheral NP (mean probability of 30% pain reduction)	Unclear efficacy (not enough information provided by authors)	L
Norton, 2017 [28]	Inception–Feb 2016; 5	NR	3	Inflammatory bowel disease	I: Plant-based cannabis C: No comparator (observational study)	N/A observational data	N/A observational data	CL
Langhorst, 2015 [44]	Inception–Mar 12, 2014; 4	Non-profit	1	Inflammatory bowel disease (Crohn's, ulcerative colitis, IBS)	I: Plant-based cannabis C: Placebo cigarettes (THC removed)	• Response rate • QoL • Remission rate	Only one study included	M
CADTH, 2014 [81]	Jan 1, 1982 to Aug 8 2014; 5	NR	4	Nausea and vomiting (non-chemotherapy-induced)	I: Synthetic cannabinoids C: Metoclopramide	• Postoperative nausea and vomiting • Patient rating of nausea and vomiting (VAS)	Only one study included	L

*CADTH* Canadian Agency for Drugs and Technologies in Health, *IBS* irritable bowel syndrome, *NR* not reported, *QoL* quality of life, *SBS* study-by-study, *VAS* visual analog scale

*A colon indicates that there were separate analyses for each comparator

The reviews were published between 2000 and 2018 (median year 2014), and almost half (47%) were focused solely on medical cannabis. Four (6%) reviews covered both medical and other cannabis use (recreational and substance abuse), 19 (26%) reported multiple pharmaceutical interventions (cannabis being one), six (8%) reported various CAM interventions (cannabis being one), and nine (13%) were mixed pharmaceutical and CAM interventions (cannabis being one). Multiple databases were searched by almost all of the reviews (97%), with Medline/PubMed or Embase common to all.

### Cannabis use

**Fig. 2:**
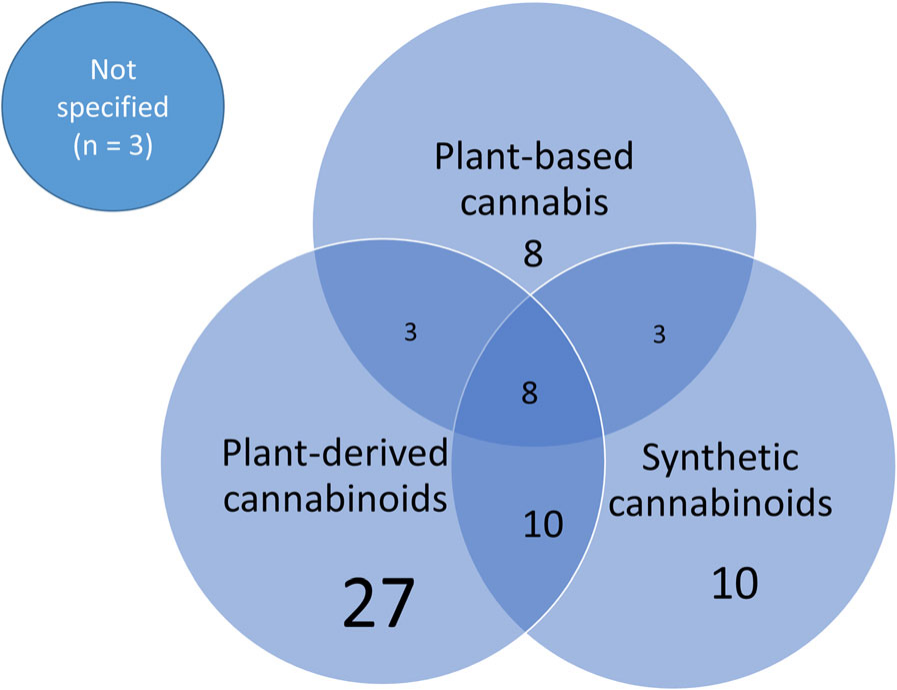
Review coverage of the various cannabis-based interventions

Figure 2 illustrates the different cannabis-based interventions covered by the included reviews. Plant-based cannabis consists of whole plant products such as marijuana or hashish. Plant-derived cannabinoids are active constituents of the cannabis plant, such as tetrahydrocannabinol (THC), cannabidiol (CBD), or a combination of THC:CBD (also called nabiximols, under the brand name Sativex) [3]. Synthetic cannabinoids are manufactured rather than extracted from the plant and include drugs such as nabilone and dronabinol.

Twenty-seven reviews included solely interventions from plant-derived cannabinoids, 10 studied solely synthetic cannabinoids, and eight included solely studies on plant-based cannabis products. Twenty-four reviews covered a combination of different types of cannabis, and the remaining three systematic reviews did not report which type of cannabinoid was administered in the included studies.

### Population

**Fig. 3:**
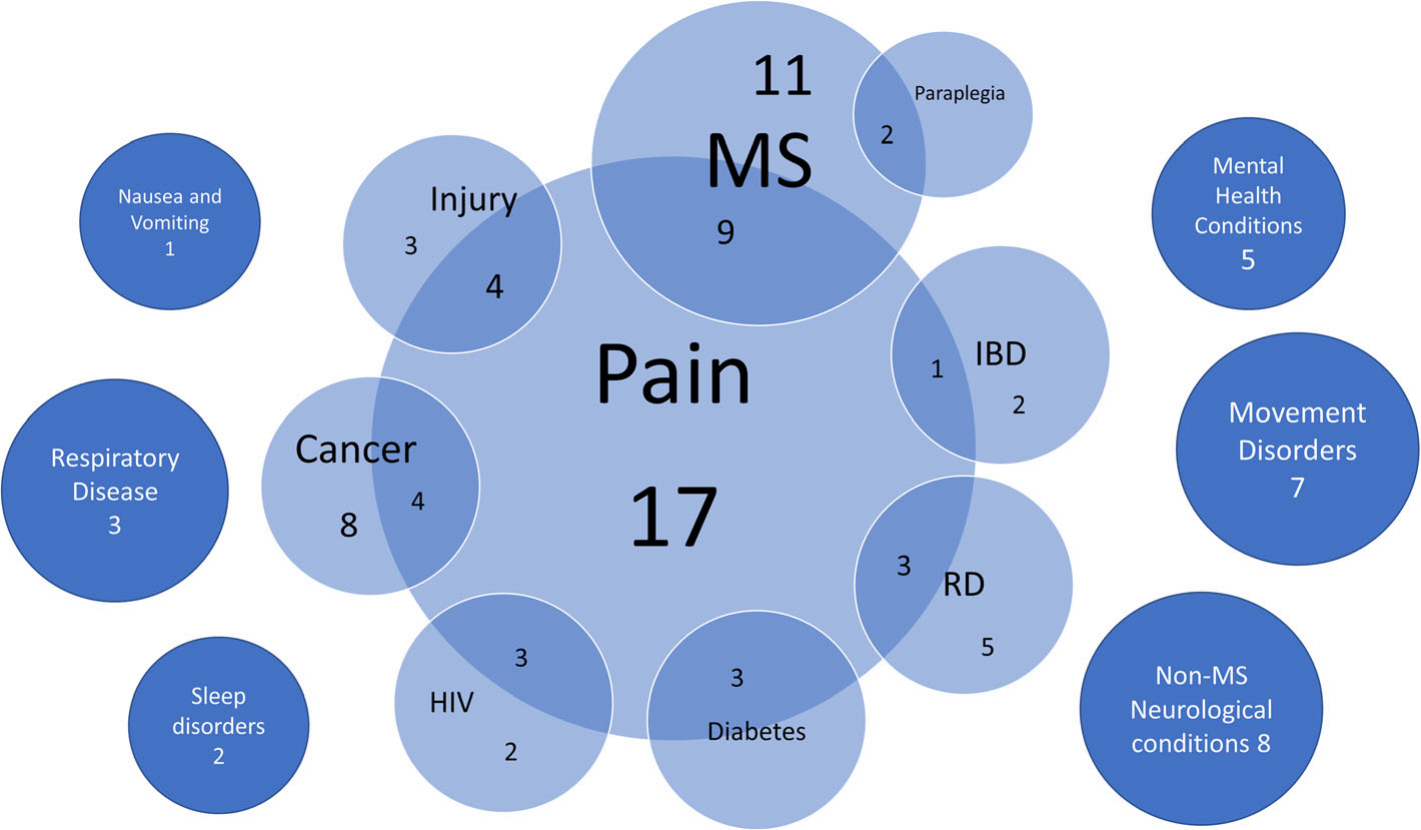
Conditions or symptoms across reviews that were treated with cannabis. IBD inflammatory bowel disease, MS multiple sclerosis, RD rheumatic disease

The systematic reviews covered a wide range of conditions and illnesses, the most notable being pain management. Seventeen reviews looked at specific types of pain including neuropathic [31, 42, 62, 69, 85, 90], chronic [26, 32, 52, 58, 80], cancer [84, 87], non-cancer [41, 68], and acute [38] types of pain (one review covered all types of pain) [65]. Twenty-seven reviews (38%) also focused on management of pain as a symptom of conditions such as multiple sclerosis (MS) [6, 23, 27, 43, 46, 52, 63, 85, 92], injury [29, 35, 36, 69], cancer [37, 43, 65, 88], inflammatory bowel disease (IBD) [28], rheumatic disease (RD) [49, 51, 73], diabetes [68–70], and HIV [48, 53, 67]. In Fig. 3, the types of illnesses addressed by the set of included reviews are graphically represented, with overlap between various conditions and pain. Some systematic reviews covered multiple diseases, and therefore the total number of conditions represented in Fig. 3 is greater than the total number of included reviews.

One review included a pediatric-only population, in the evaluation of marijuana for nausea and vomiting following chemotherapy [54]. Although trials in both adult and child populations were eligible for thirteen (18%) reviews, only two additional reviews included studies in children; these reviews evaluated cannabis in cancer [60] and a variety of conditions [25]. Many of the reviews (*n* = 25, 35%) included only adults ≥ 18 years of age. Almost half of the reviews (*n* = 33, 46%) did not report a specific population for inclusion.

**Fig. 4:**
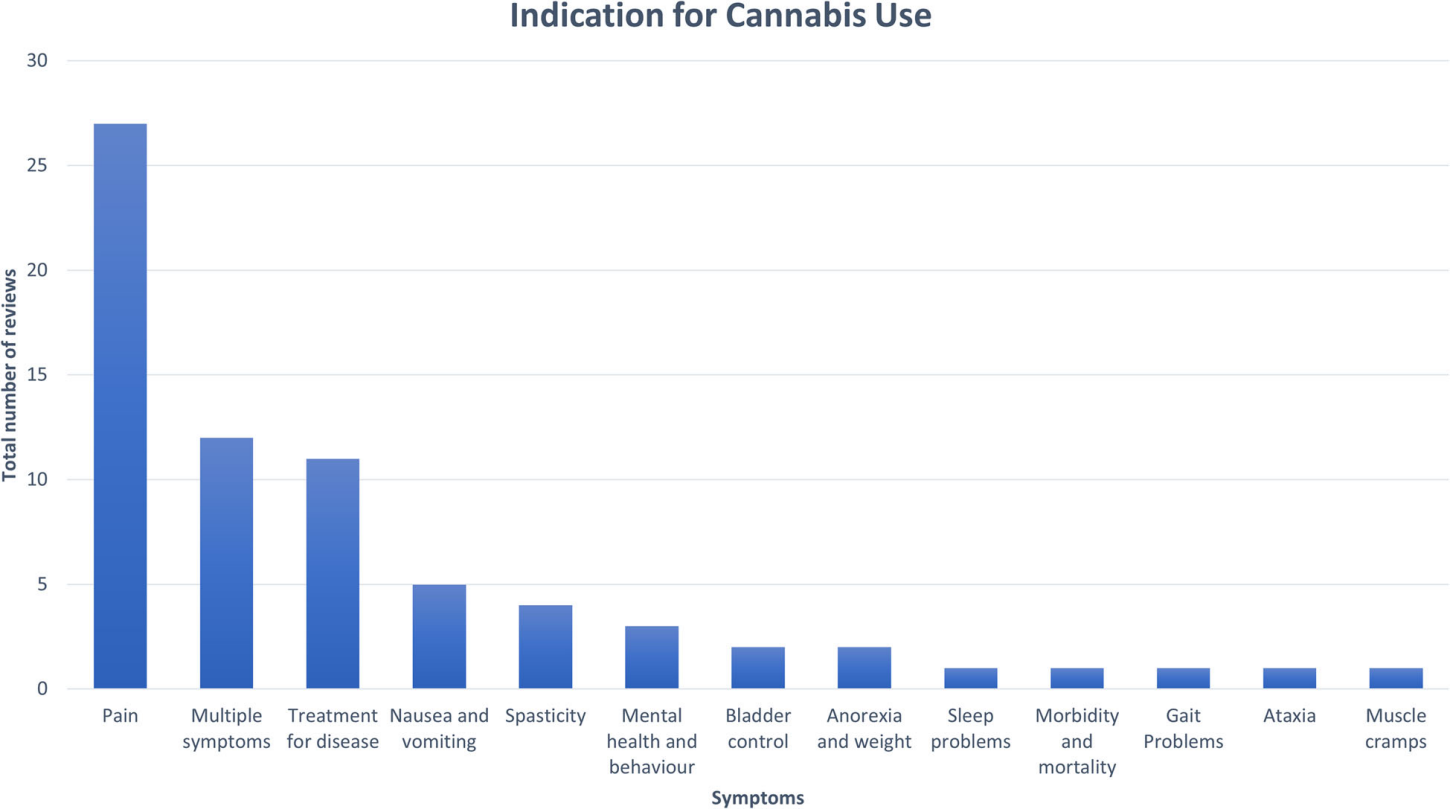
Indications for cannabis use across included reviews

Cannabis was prescribed for a wide range of medical issues. The indication for cannabis use is illustrated in Fig. 4. Pain management (*n* = 27) was the most common indication for cannabis use. A number of reviews sought to address multiple disease symptoms (*n* = 12) or explored a more holistic treatment for the disease itself (*n* = 11). After pain, the most common symptoms being treated with cannabis were spasticity in MS, movement disturbances (such as dyskinesia, tics, and spasms), weight or nausea/vomiting, and mental health symptoms.

**Fig. 5:**
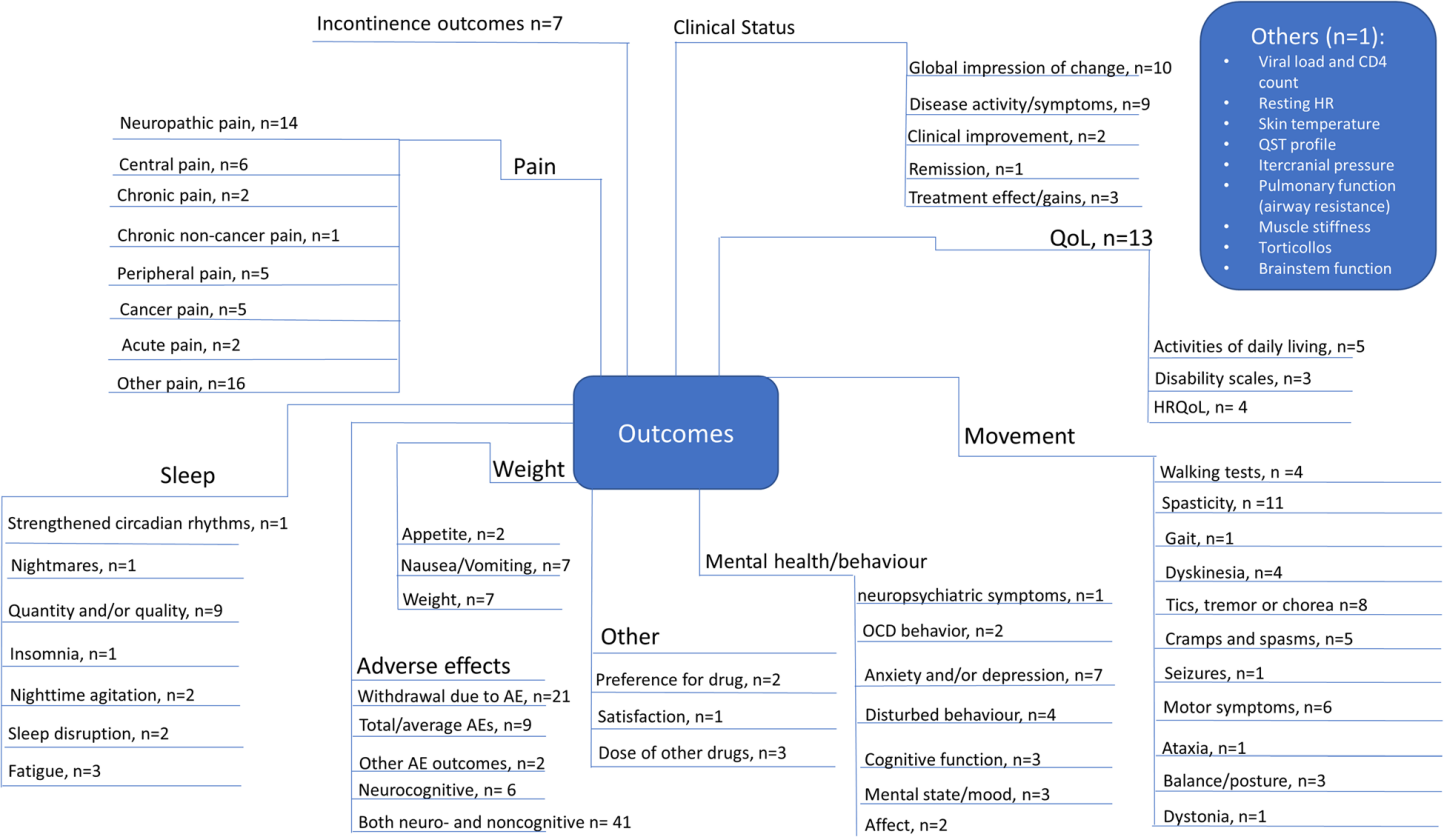
Outcomes

Figure 5 summarizes the breadth of outcomes analyzed in the included reviews. The most commonly addressed outcomes were withdrawal due to adverse effects, “other pain,” neuropathic pain, spasticity, and the global impression of the change in clinical status. Many outcomes were reported using a variety of measures across reviews. For example, spasticity was measured both objectively (using the Ashworth scale) and subjectively (using a visual analog scale [VAS] or numerical rating scale [NRS]). Similarily, outcomes for pain included VAS or NRS scales, reduction in pain, pain relief, analgesia, pain intensity, and patient assessment of change in pain.

### Quality of the systematic reviews

Quality assessments of the included reviews based upon AMSTAR-2 are detailed in Additional file 7 and Additional file 8. Only one review was rated as high quality [45]. All other reviews were deemed to be of moderate (*n* = 36) or low/critically low (*n* = 35) methodological quality. Assessments for the domains deemed of critical importance for determining quality ratings are described below.

Only 20% of reviews used a comprehensive search strategy; another 47% were given a partial score because they had not searched the reference lists of the included reviews, trial registries, gray literature, and/or the search date was older than 2 years. The remaining reviews did not report a comprehensive search strategy.

Over half of the reviews (51%) used a satisfactory technique for assessing risk of bias (ROB) of the individual included studies, while 35% were partially satisfactory because they had not reported whether allocation sequence was truly random and/or they had not assessed selective reporting. The remaining reviews did not report a satisfactory technique for assessing ROB.

Most reviews (71%) could not be assessed for an appropriate statistical method for combining results in a meta-analysis, as they synthesized study data narratively. Approximately 19% of reviews used an appropriate meta-analytical approach, leaving 10% that used inappropriate methods.

The final critical domain for the AMSTAR-2 determines whether review authors accounted for ROB in individual studies when discussing or interpreting the results of the review. The majority of reviews (83%) did so in some capacity.

### Mapping results of included systematic reviews

**Fig. 6:**
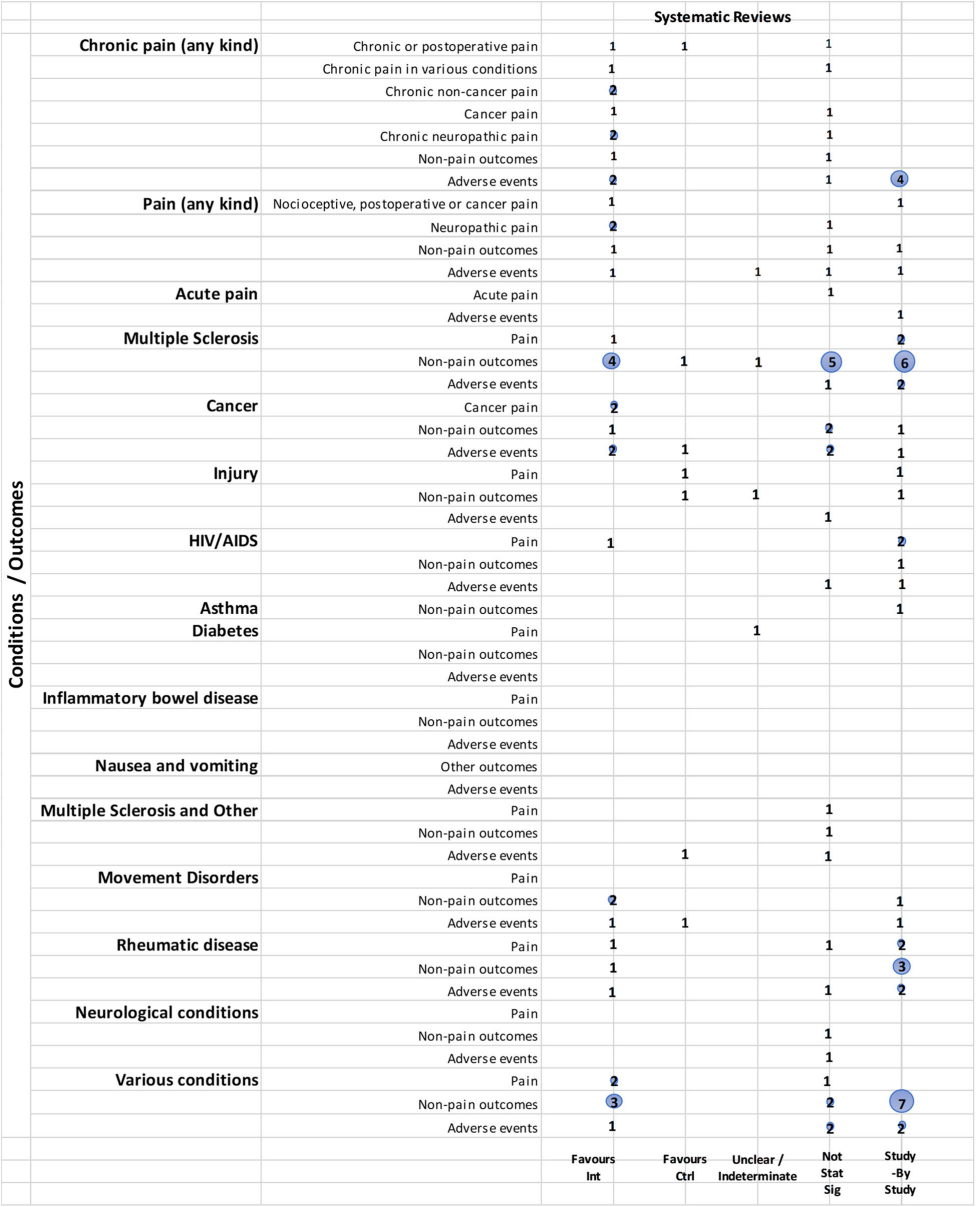
Cannabis vs. placebo. Authors’ presentations of the findings were mapped using the categorization shown in Table 2. According to the reviews’ intended scope for the condition being treated, outcomes were mapped into “pain,” “non-pain outcomes,” and “adverse events.” For each condition and outcome pair (i.e., each row in the grid), the number of reviews reporting findings is shown according to the results categorization. For pain, reviews numbered in different categories signal discordant findings across those reviews. For non-pain outcomes, reviews presenting findings in the different categories would signal different results for different outcomes, as well as discordant findings within and across reviews. Adverse events are grouped as a whole and “favors intervention” would be interpreted as a decrease in events with cannabis when compared with the control group. Favors int = favors intervention; Favors Ctrl = favors control; Not stat sig = not statistically significant

**Fig. 7:**
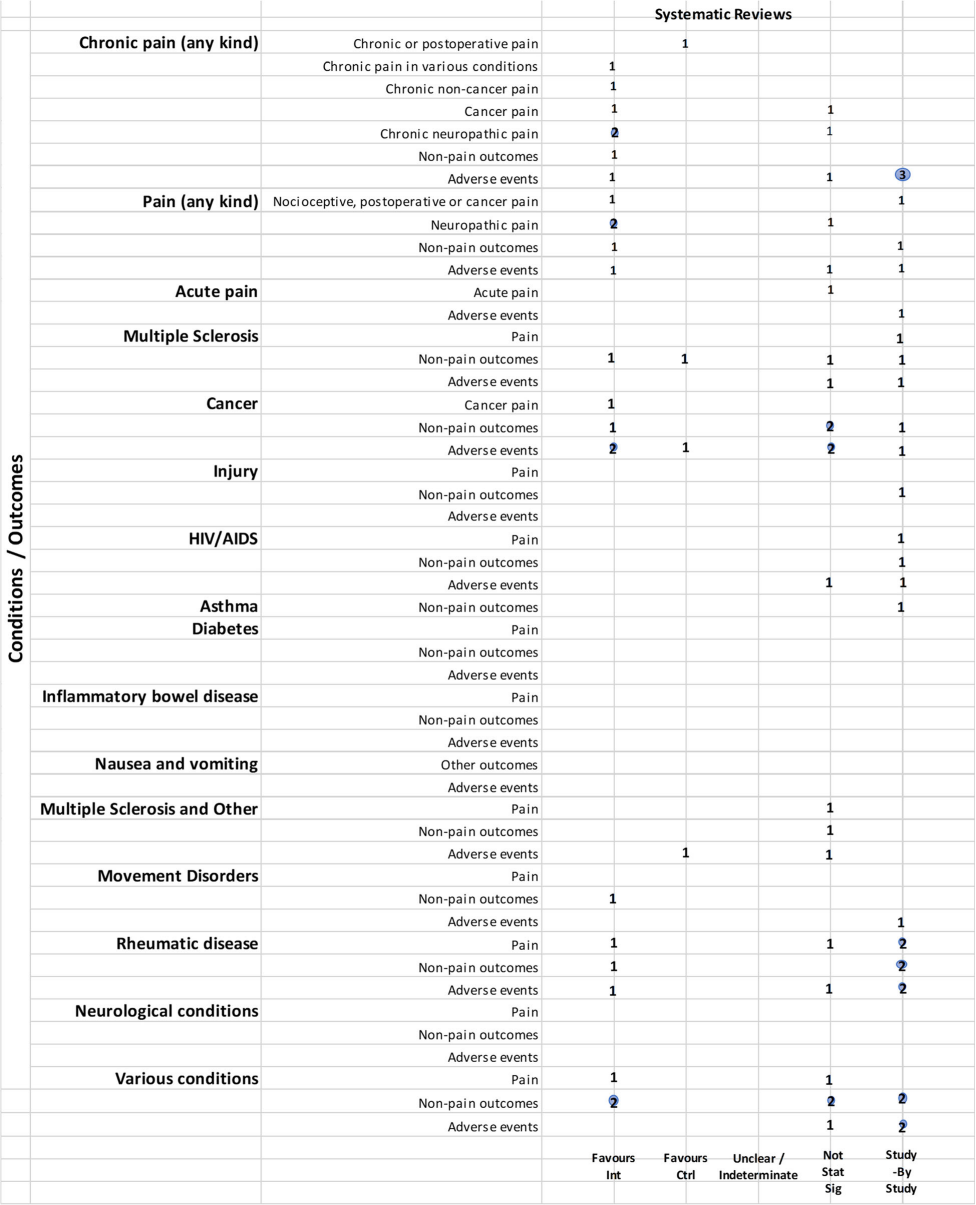
Cannabis vs. placebo, high and moderate quality reviews. Authors’ presentations of the findings were mapped using the categorizations shown in Table 2. According to the reviews’ intended scope for the condition being treated, outcomes were mapped into “pain,” “non-pain outcomes,” and “adverse events.” For each condition and outcome pair (i.e., each row in the grid), the number of reviews reporting findings is shown according to the results categorization. For pain, reviews numbered in different categories signal discordant findings across those reviews. For non-pain outcomes, reviews presenting findings in the different categories would signal different results for different outcomes, as well as discordant findings within and across reviews. Adverse events are grouped as a whole and “favors intervention” would be interpreted as a decrease in events with cannabis when compared with the control group. *Favors int* = favors intervention; *Favors Ctrl* = favors control; *Not stat sig* = not statistically significant

**Fig. 8:**
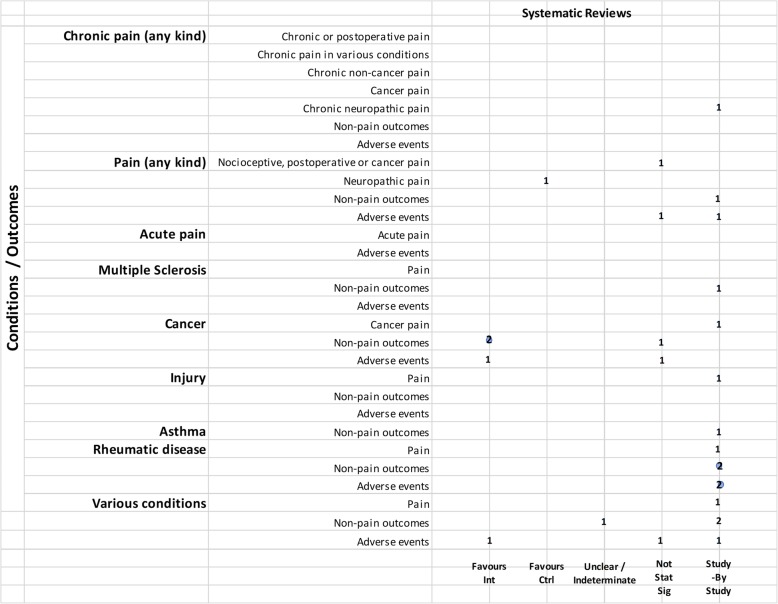
Cannabis vs. active drugs. Authors’ presentations of the findings were mapped using the categorizations shown in Table 2. According to the reviews’ intended scope for the condition being treated, outcomes were mapped into “pain,” “non-pain outcomes,” and “adverse events.” For each condition and outcome pair (i.e., each row in the grid), the number of reviews reporting findings is shown according to the results categorization. For pain, reviews numbered in different categories signal discordant findings across those reviews. For non-pain outcomes, reviews presenting findings in the different categories would signal different results for different outcomes, as well as discordant findings within and across reviews. Adverse events are grouped as a whole and “favors intervention” would be interpreted as a decrease in events with cannabis when compared with the control group. *Favors int* = favors intervention; *Favors Ctrl* = favors control; *Not stat sig* = not statistically significant

**Fig. 9:**
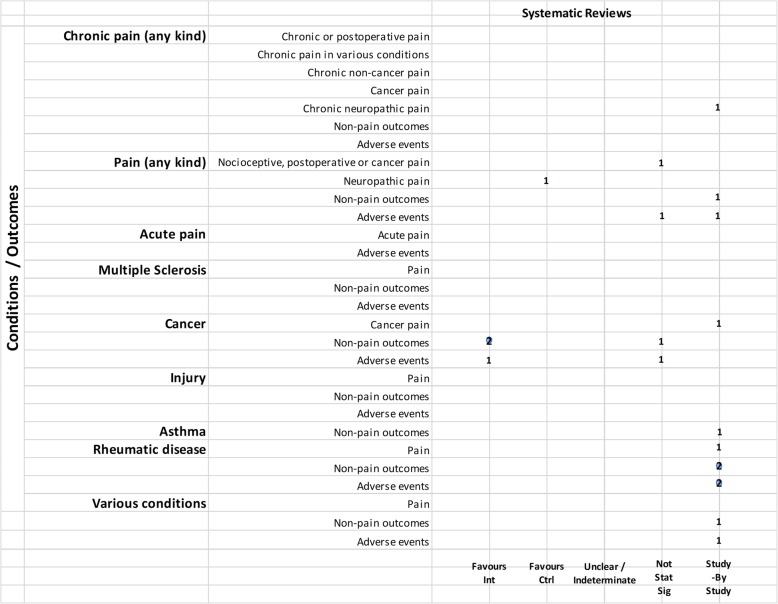
Cannabis vs. active drugs, high and moderate quality reviews. Authors’ presentations of the findings were mapped using the categorizations shown in Table 2. According to the reviews’ intended scope for the condition being treated, outcomes were mapped into “pain,” “non-pain outcomes,” and “adverse events.” For each condition and outcome pair (i.e., each row in the grid), the number of reviews reporting findings is shown according to the results categorization. For pain, reviews numbered in different categories signal discordant findings across those reviews. For non-pain outcomes, reviews presenting findings in the different categories would signal different results for different outcomes, as well as discordant findings within and across reviews. Adverse events are grouped as a whole and “favors intervention” would be interpreted as a decrease in events with cannabis when compared with the control group. *Favors int* = favors intervention; *Favors Ctrl* = favors control; *Not stat sig* = not statistically significant

**Fig. 10:**
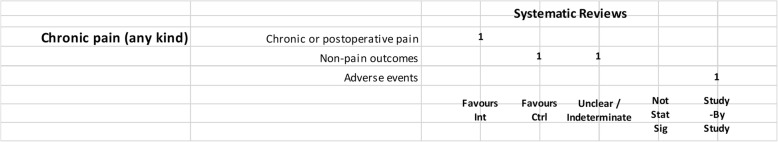
Cannabis vs. placebo + active drug. Authors’ presentations of the findings were mapped using the categorizations shown in Table 2. According to the reviews’ intended scope for the condition being treated, outcomes were mapped into “pain,” “non-pain outcomes,” and “adverse events.” For each condition and outcome pair (i.e., each row in the grid), the number of reviews reporting findings is shown according to the results categorization. For pain, reviews numbered in different categories signal discordant findings across those reviews. For non-pain outcomes, reviews presenting findings in the different categories would signal different results for different outcomes, as well as discordant findings within and across reviews. Adverse events are grouped as a whole and “favors intervention” would be interpreted as a decrease in events with cannabis when compared with the control group. *Favors int* = favors intervention; *Favors Ctrl* = favors control; *Not stat sig* = not statistically significant

**Fig. 11:**
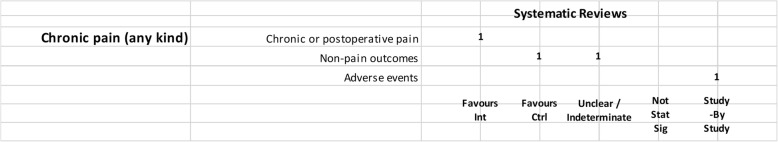
Cannabis vs. placebo + active drug, high and moderate quality reviews. Authors’ presentations of the findings were mapped using the categorizations shown in Table 2. According to the reviews’ intended scope for the condition being treated, outcomes were mapped into “pain,” “non-pain outcomes,” and “adverse events.” For each condition and outcome pair (i.e., each row in the grid), the number of reviews reporting findings is shown according to the results categorization. For pain, reviews numbered in different categories signal discordant findings across those reviews. For non-pain outcomes, reviews presenting findings in the different categories would signal different results for different outcomes, as well as discordant findings within and across reviews. Adverse events are grouped as a whole and “favors intervention” would be interpreted as a decrease in events with cannabis when compared with the control group. *Favors int* = favors intervention; *Favors Ctrl* = favors control; *Not stat sig* = not statistically significant

**Fig. 12:**
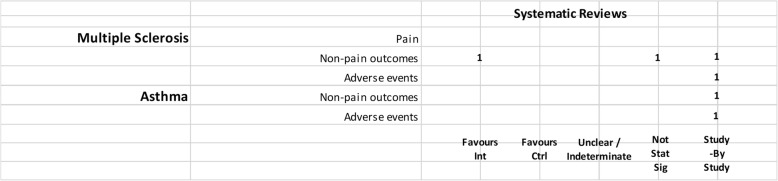
One cannabis formulation vs. other. Authors’ presentations of the findings were mapped using the categorizations shown in Table 2. According to the reviews’ intended scope for the condition being treated, outcomes were mapped into “pain,” “non-pain outcomes,” and “adverse events.” For each condition and outcome pair (i.e., each row in the grid), the number of reviews reporting findings is shown according to the results categorization. For pain, reviews numbered in different categories signal discordant findings across those reviews. For non-pain outcomes, reviews presenting findings in the different categories would signal different results for different outcomes, as well as discordant findings within and across reviews. Adverse events are grouped as a whole and “favors intervention” would be interpreted as a decrease in events with cannabis when compared with the control group. *Favors int* = favors intervention; *Favors Ctrl* = favors control; *Not stat sig* = not statistically significant

**Fig. 13:**
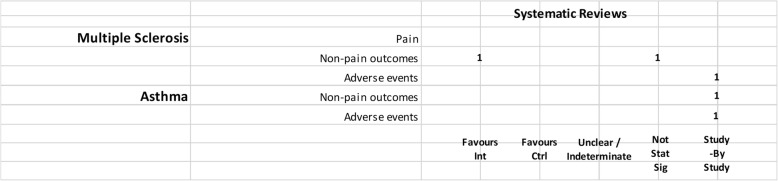
One cannabis formulation vs. other, high and moderate quality reviews. Authors’ presentations of the findings were mapped using the categorizations shown in Table 2. According to the reviews’ intended scope for the condition being treated, outcomes were mapped into “pain,” “non-pain outcomes,” and “adverse events.” For each condition and outcome pair (i.e., each row in the grid), the number of reviews reporting findings is shown according to the results categorization. For pain, reviews numbered in different categories signal discordant findings across those reviews. For non-pain outcomes, reviews presenting findings in the different categories would signal different results for different outcomes, as well as discordant findings within and across reviews. Adverse events are grouped as a whole and “favors intervention” would be interpreted as a decrease in events with cannabis when compared with the control group. *Favors int* = favors intervention; *Favors Ctrl* = favors control; *Not stat sig* = not statistically significant

**Fig. 14:**
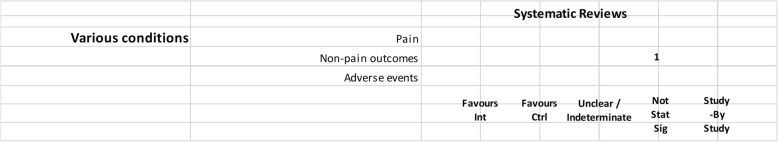
Cannabis vs. all comparators combined. Authors’ presentations of the findings were mapped using the categorizations shown in Table 2. According to the reviews’ intended scope for the condition being treated, outcomes were mapped into “pain,” “non-pain outcomes,” and “adverse events.” For each condition and outcome pair (i.e., each row in the grid), the number of reviews reporting findings is shown according to the results categorization. For pain, reviews numbered in different categories signal discordant findings across those reviews. For non-pain outcomes, reviews presenting findings in the different categories would signal different results for different outcomes, as well as discordant findings within and across reviews. Adverse events are grouped as a whole and “favors intervention” would be interpreted as a decrease in events with cannabis when compared with the control group. *Favors int* = favors intervention; *Favors Ctrl* = favors control; *Not stat sig* = not statistically significant

We mapped reviews according to authors’ comparisons, the conditions or symptoms they were evaluating, and the categorization of the results (see Table 2). In some cases, reviews contributed to more than one comparison (e.g., cannabis versus placebo or active drug). As pain was the most commonly addressed outcome, we mapped this outcome separately from all other endpoints. This information is shown for all reviews and then restricted to reviews of moderate-to-high quality (as determined using the AMSTAR-2 criteria): cannabis versus placebo (Figs. 6 and 7), cannabis versus active drugs (Figs. 8 and 9), cannabis versus a combination of placebo and active drug (Figs. 10 and 11), one cannabis formulation versus other (Figs. 12 and 13), and cannabis analyzed against all other comparators (Fig. 14). Details on how to read the figures are provided in the corresponding figure legends. The median number of included studies across reviews was four, and ranged from one to seventy-nine (not shown in figures).

#### Cannabis versus placebo

Most reviews (59/72, 82%) compared cannabis with placebo. Of these reviews, 34 (58%) addressed pain outcomes and 47 (80%) addressed non-pain outcomes, with most outcomes addressed by three reviews or fewer (Fig. 6). Some reviews had a mix of quantitative syntheses and study-by-study data reported (13/59, 22%), while another group of reviews (14/59, 24%) only reported results study-by-study. Overall, 24% (14/59) of the cannabis versus placebo reviews had only one included study.

1. Pain outcomes
  i. *Reviews focused on addressing pain across conditions.* In most cases, findings were discordant across reviews for the pain outcomes measured. For chronic non-cancer pain, however, two reviews favored cannabis over placebo for decreasing pain. One review assessing acute pain for postoperative pain relief found no difference between various cannabinoid medications and placebo. The distribution of findings was similar when restricting to moderate-to-high-quality reviews.
  ii. *Reviews focused on treating a condition or family of related conditions*. Various results were observed for pain. For MS and HIV/AIDS, one review each reported quantitative results favoring cannabis for decreased pain but with other reviews reporting results study-by-study, it is difficult to know, broadly, how consistent those findings are. For cancer, two reviews reported results favoring cannabis for decreased pain. For rheumatic disease, findings are discordant between two reviews, and another two reviews reported results study-by-study. One review that included studies of MS or paraplegia found no difference in pain between groups. For treating injury, one review showed that the placebo group had less pain and one review reported data study-by-study. No reviews addressed pain in movement disorders, neurological conditions, and IBD.

For those reviews assessing pain as part of a focus on treating a range of conditions, two showed cannabis reduced pain [43, 52], but one showed mixed results depending on how pain was measured [43]. These reviews covered several different conditions, including injury, chronic pain, rheumatoid arthritis, osteoarthritis, fibromyalgia, HIV/AIDS, cancer, and MS or paraplegia.

When restricting to moderate-to-high-quality reviews, only one review each in multiple sclerosis and HIV/AIDS with a study-by-study analysis on pain remained. One review on cancer favored cannabis for pain reduction. Findings remained the same for MS or paraplegia and rheumatic disease. No review for injury and paint outcomes was of higher quality.

2. Non-pain outcomes

The types of non-pain outcomes included in the reviews varied by condition/illness. The most commonly reported outcomes (see Fig. 5 for overall outcomes) when comparing cannabis to placebo included muscle- or movement-related outcomes (*n* = 20), quality of life (*n* = 14), and sleep outcomes (*n* = 10).

There was no consistent pattern for non-pain outcomes either within or across medical conditions. Many (*n* = 24, 33%) reviews assessing non-pain outcomes reported the results of those analyses study-by-study. Conflicting results are observed in some cases due to the use of different measures, such as different ways of quantifying spasticity in patients with multiple sclerosis [56, 91]. One review each addressing neurological conditions [50] (outcome: muscle cramps) and MS/paraplegia [27] (outcomes: spasticity, spasm, cognitive function, daily activities, motricity, and bladder function) showed no difference between groups.

3. Adverse effects

Adverse effects were reported in most reviews comparing cannabis with placebo (49/59, 83%). Most adverse events were reported study-by-study, with few reviews (*n* = 16/59, 27%) conducting a narrative or quantitative synthesis. Serious adverse effects were reported in 21/59 (36%) reviews, and minor adverse effects were reported in 30/59 (51%) reviews. The remaining reviews did not define the difference between serious and minor adverse events. The most commonly reported serious adverse events included psychotic symptoms (*n* = 6), severe dysphoric reactions (*n* = 3), seizure (*n* = 3), and urinary tract infection (*n* = 2). The most commonly reported minor adverse events included somnolence/drowsiness (*n* = 28), dizziness (*n* = 27), dry mouth (*n* = 20), and nausea (*n* = 18). Many reviews (*n* = 37/59, 63%) comparing cannabis to placebo reported both neurocognitive and non-cognitive adverse effects. Withdrawals due to adverse events were reported in 22 (37%) reviews.

Of the moderate-/high-quality reviews, adverse effect analyses were reported in reviews on pain, multiple sclerosis, cancer, HIV/AIDS, movement disorders, rheumatic disease, and several other conditions. Two reviews on pain showed fewer adverse events with cannabis for euphoria, events linked to alternations in perception, motor function, and cognitive function, withdrawal due to adverse events, sleep, and dizziness or vertigo [58, 90]. One review on MS showed that there was no statistically significant difference between cannabis and placebo for adverse effects such as nausea, weakness, somnolence, and fatigue [91], while another review on MS/paraplegia reported fewer events in the placebo group for dizziness, somnolence, nausea, and dry mouth [27]. Within cancer reviews, one review found no statistically significant difference between cannabis and placebo for dysphoria or sedation but reported fewer events with placebo for “feeling high,” and fewer events with cannabis for withdrawal due to adverse effects [40]. In rheumatic disease, one review reported fewer total adverse events with cannabis and found no statistically significant difference between cannabis and placebo for withdrawal due to adverse events [51].

#### Cannabis versus other drugs

Relatively fewer reviews compared cannabis with active drugs (*n* = 23/72, 32%) (Fig. 8). Many of the reviews did not synthesize studies quantitatively, and results were reported study-by-study. The most common conditions in reviews comparing cannabis to active drugs were pain, cancer, and rheumatic disease. Comparators included ibuprofen, codeine, diphenhydramine, amitriptyline, secobarbital, prochlorperazine, domperidone, metoclopramide, amisulpride, neuroleptics, isoproterenol, megestrol acetate, pregabalin, gabapentin, and opioids.

1. Pain outcomes
  i. *Reviews focused on addressing pain across conditions.* When comparing across reviews, a mix of results are observed (see Fig. 8), and some were reported study-by-study. One review found no statistically significant difference between cannabinoids and codeine for nociceptive pain, postoperative pain, and cancer pain [65]. Another review favored “other drugs” (amitriptyline and pregabalin) over cannabinoids for neuropathic pain [90]. The distribution of findings was similar when restricting to moderate-to-high-quality reviews.
  ii. *Reviews focused on treating a condition or family of related conditions.* One review on cancer compared cannabinoids and codeine or secobarbital and reported pain results study-by-study. Another review on fibromyalgia comparing synthetic cannabinoids with amitriptyline also reported pain data study-by-study [39].
2. Non-pain outcomes

Two reviews on cancer favored cannabinoids over active drugs (prochlorperazine, domperidone, metoclopramide, and neuroleptics) for patient preference and anti-emetic efficacy [40, 60]. Non-pain outcomes were reported study-by-study for the outcome of sleep in neuropathic pain [90] and rheumatic disease [39, 49]. In a review covering various conditions (pain, MS, anorexia, cancer, and immune deficiency), results were unclear or indeterminate for subjective measures of sleep [46].

3. Adverse effects

Adverse effects were reported in 20/24 (83%) of the reviews comparing cannabis to active drugs, and only 6/20 (30%) reported a narrative or quantitative synthesis. Many reviews that reported narrative data did not specify whether adverse effects could be attributed to a placebo or active drug comparator.

Of the moderate-to-high-quality reviews, two pain reviews found no statistically significant difference for cannabis compared to codeine or amitriptyline for withdrawals due to adverse events [65, 90]. Results from one cancer review were mixed, with fewer adverse events for cannabis (compared to prochlorperazine, domperidone, or metoclopramide) or no difference between groups, depending on the type of subgroup analysis that was conducted [40].

#### Cannabis + active drugs versus placebo + active drugs

Two reviews compared cannabis with placebo cannabis in combination with an active drug (opioids and gabapentin) (Figs. 10 and 11). Both were scored to be of moderate quality. Although one review showed that cannabis plus opioids decreased chronic pain [80], another review on pain in MS included only a single study [81], precluding the ability to determine concordance of results. Cannabis displayed varied effects on non-pain outcomes, including superiority of placebo over cannabis for some outcomes. One review reported withdrawal due to adverse events study-by-study and also reported that side effects such as nausea, drowsiness, and dizziness were more frequent with higher doses of cannabinoids (data from two included studies) [80].

#### Cannabis versus other cannabis comparisons

Six (8%) reviews compared different cannabis formulations or doses (Figs. 12 and 13). Almost all were reported as study-by-study results, with two reviews including only one RCT. One review for PTSD found only observational data [33] and another review on anxiety and depression combined data from one RCT with cross-sectional study data [19]. A single review on MS reported a narrative synthesis that found a benefit for spasticity. However, it was unclear if the comparator was placebo or THC alone [56]. Four reviews reported adverse effects study-by-study, with a single review comparing side effects from different dosages; in this review, combined extracts of THC and CBD were better tolerated than extracts of THC alone [56].

#### Cannabis versus all comparators

One review combined all comparators for the evaluation (Fig. 14). The review (combining non-users, placebo and ibuprofen) covered a range of medical conditions and was rated as low quality [30]. No adverse effects were evaluated for this comparison.

### Mapping the use of quality assessment and frameworks to interpret the strength of evidence

Although 83% of reviews incorporated risk of bias assessments in their interpretation of the evidence, only 11 (15%) reviews used a framework such as GRADE to evaluate important domains other than risk of bias that would inform the strength of the evidence.

### Mapping authors’ conclusions or recommendations

Most reviews (43/72 60%) indicated an inability to draw conclusions, whether due to uncertainty, inconsistent findings, lack of (high quality) evidence, or focusing their conclusion statement on the need for more research. Almost 15% of reviews (10/72) reported recommendations or conclusions that included some uncertainty. One review (1%) provided a statement of the extent of the strength of the evidence, which differed according to outcome.

Eleven reviews provided clearer conclusions (14%). Four indicated that cannabis was not effective or not cost-effective compared to placebo in relation to multiple sclerosis, acute pain, cancer, and injury. Three reviews addressing various conditions provided varying conclusions: one stated cannabis was not effective, one indicated it was modestly safe and effective, and one concluded that cannabis was safe and efficacious as short-term treatment; all reviews were of low quality. The three remaining reviews stated moderate or modest effects for improving chronic pain, compared with placebo or other analgesia; two of those reviews were of medium AMSTAR-2 quality, and one used the GRADE framework for interpreting the strength of the evidence.

The eight remaining included reviews (11%) did not provide a clear conclusion statement or reported only limitations.

### Mapping authors’ limitations of the research

Several of the reviews indicated that few studies, small sample sizes, short duration of treatment, and issues related to outcomes (e.g., definition, timing, and types) were drawbacks to the literature. Some reviews noted methodological issues with and heterogeneity among studies as limitations. A few authors stated that restricting eligibility to randomized trials, English-language studies, or full publications may have affected their review results.

## Discussion

With the increasing use of medical cannabis, an understanding of the landscape of available evidence syntheses is needed to support evidence-informed decision-making, policy development, and to inform a research agenda. In this scoping review, we identified 72 systematic reviews evaluating medical cannabis for a range of conditions and illnesses. Half of the reviews were evaluated as being of moderate quality, with only one review scoring high on the AMSTAR-2 assessment tool.

There was disparity in the reported results across reviews, including non-synthesized (study-by-study) data, and many were unable to provide a definitive statement regarding the effectiveness of cannabis (as measured by pain reduction or other relevant outcomes), nor the extent of increased side effects and harms. This is consistent with the limitations declared in general across reviews, such as the small numbers of relevant studies, small sample sizes of individual studies, and methodological weaknesses of available studies. This common theme in review conclusions suggests that while systematic reviews may have been conducted with moderate or high methodological quality, the strength of their conclusions are driven by the availability and quality of the relevant underlying evidence, which was often found to be limited.

Relatively fewer reviews addressed adverse effects associated with cannabis, except to narratively summarize study level data. Although information was provided for placebo-controlled comparisons, none of the comparative effectiveness reviews quantitatively assessed adverse effects data. For the placebo-controlled data, although the majority of adverse effects were mild, the number of reviews reporting serious adverse effects such as psychotic symptoms [25, 42] and suicidal ideation [68, 85] warrants caution.

A mix of reviews supporting and not supporting the use of cannabis, according to authors’ conclusions, was identified. Readers may wish to consider the quality of the reviews, the use of differing quality assessment tools, additional considerations covered by the GRADE framework, and the potential for spin as possible reasons for these inconsistencies. It is also possible that cannabis has differing effects depending on its type (e.g., synthetic), dose, indication, the type of pain being evaluated (e.g., neuropathic), and the tools used for outcome assessment, which can be dependent on variations in condition. Of potential interest to readers may be a closer examination of the reviews evaluating chronic pain, in order to locate the source(s) of discordance. For example, one review was deemed of moderate quality, used the GRADE framework, and rated the quality of evidence for the effectiveness of cannabis for reducing neuropathic pain as moderate, suggesting that further investigation of cannabis for neuropathic pain may be warranted [80]. The exploration aspects outlined in this paragraph are beyond the purview of scoping review methodology; a detailed assessment of the reviews, including determining the overlap of included studies among similar reviews, potential reasons for the observed discordance of findings, what re-analysis of study-by-study analyses would yield, and an undertaking of missing GRADE assessments would fall outside the bounds of a scoping review and require the use of overview methodology [14].

Our findings are consistent with a recently published summary of cannabis-based medicines for chronic pain management [3]. This report found inconsistent results in systematic reviews of cannabis-based medicines compared to placebo for chronic neuropathic pain, pain management in rheumatic diseases and painful spasms in MS. The authors also concluded that cannabis was not superior to placebo in reducing cancer pain. Four out of eight included reviews scored high on the original AMSTAR tool. The variations between the two tools can be attributed to the differences in our overall assessments. Lastly, the summary report included two reviews that were not located in our original search due to language [93] and the full-text [94] of an abstract [95] that was not located in our search.

This scoping review has identified a plethora of synthesized evidence in relation to medical cannabis. For some conditions, the extent of review replication may be wasteful. Many reviews have stated that additional trials of methodologically robust design and, where possible, of sufficient sample size for precision, are needed to add to the evidence base. This undertaking may require the coordination of multi-center studies to ensure adequate power. Future trials may also help to elucidate the effect of cannabis on different outcomes.

Given authors’ reporting of issues in relation to outcomes, future prospective trials should be guided by a standardized, “core” set of outcomes to strive for consistency across studies and ensure relevance to patient-centered care. Development of those core outcomes should be developed using the Core Outcome Measures in Effectiveness Trials (COMET) methodology [96], and further consideration will need to be made in relation to what outcomes may be common across all cannabis research and which outcomes are condition-specific. With maturity of the evidence base, future systematic reviews should seek and include non-journal-published (gray literature) reports and ideally evaluate any non-English-language papers; authors should also adequately assess risk of bias and undertake appropriate syntheses of the literature.

The strengths of this scoping review include the use of an a priori protocol, peer-reviewed search strategies, a comprehensive search for reviews, and consideration of observational designs for adverse effects data. For feasibility, we restricted to English-language reviews, and it is unknown how many of the 39 reviews in other languages that we screened would have met our eligibility criteria. The decision to limit the inclusion of reviews of observational data to adverse effects data was made during the process of full-text screening and for pragmatic reasons. We also did not consider a search of the PROSPERO database for ongoing systematic reviews; however, in preparing this report, we performed a search and found that any completed reviews were already considered for eligibility or were not available at the time of our literature search. When charting results, we took a broad perspective, which may be different than if these reviews were more formally assessed during an overview of systematic reviews.

## Conclusions

Cannabis-based medicine is a rapidly emerging field of study, with implications for both healthcare practitioners and patients. This scoping review is intended to map and collate evidence on the harms and benefits of medical cannabis. Many reviews were unable to provide firm conclusions on the effectiveness of medical cannabis, and results of reviews were mixed. Mild adverse effects were frequently but inconsistently reported, and it is possible that harms may outweigh benefits. Evidence from longer-term, adequately powered, and methodologically sound RCTs exploring different types of cannabis-based medicines is required for conclusive recommendations.

## Supplementary information

**Additional file 1.** PRISMA Scoping Review Extension Completed Checklist.

**Additional file 2.** Literature Search Strategies.

**Additional file 3.** Grey Literature Sources.

**Additional file 4.** Listing of Data Extraction Items.

**Additional file 5.** Data extractions from included studies.

**Additional file 6.** Listing of Studies Excluded During Full Text Screening.

**Additional file 7.** AMSTAR Scoring Outline.

**Additional file 8.** AMSTAR Scores by Review.

## Data Availability

All data generated or analyzed during this study are included in this published article (and its supplementary information files).

## Abbreviations

CADTH: Canadian Agency for Drugs and Technologies in Health
CAM: Complementary and alternative medicine
CBD: Cannabidiol
GRADE: Grading of Recommendations Assessment, Development and Evaluation
HIV: Human immunodeficiency virus
IBD: Inflammatory bowel disease
MS: Multiple sclerosis
NRS: Numeric rating scale
RCT: Randomized controlled trial
RD: Rheumatic disease
ROB: Risk of bias
THC: Tetrahydrocannabinol
VAS: Visual analog scale
