# Supplementary material for: Benefits and harms of medical cannabis: a scoping review of systematic reviews
Source: Syst Rev. 2019 Dec 10;8:320. doi: 10.1186/s13643-019-1243-x (PMC6905063; doi:10.1186/s13643-019-1243-x)
Supplement: Supplementary file 2 — Additional file 2. Literature Search Strategies. [file 13643_2019_1243_MOESM2_ESM.docx]

**Appendix 2: Search Strategy**

Database: AMED (Allied and Complementary Medicine) <1985 to October 2017>, Embase Classic+Embase <1947 to 2017 November 02>, PsycINFO <1806 to October Week 5 2017>, EBM Reviews - Cochrane Database of Systematic Reviews <2005 to November 2, 2017>, EBM Reviews - Database of Abstracts of Reviews of Effects <1st Quarter 2016>, EBM Reviews - Health Technology Assessment <4th Quarter 2016>, Ovid MEDLINE(R) Epub Ahead of Print, In-Process & Other Non-Indexed Citations, Ovid MEDLINE(R) Daily and Ovid MEDLINE(R) <1946 to Present>

Search Strategy:

--------------------------------------------------------------------------------

1 Cannabis/ (43482)

2 exp Cannabinoids/ (75945)

3 Medical Marijuana/ (1149)

4 Marijuana Smoking/ (6815)

5 ("c.indica" or cannabi* or bhang or cannador or charas or eucannabinolide* or ganja or ganjah or hash or hashish or hemp or marihuana* or marijuana*).tw,kf. (111417)

6 (epidiolex or gwp 42003p or gwp42003p or nabidiolex).tw,kf. (79)

7 (dronabinol or thc or trans tetrahydrocannabinol* or tetrahydrocannabinol* or ea 1477 or ea1477 or marinol or qcd 84924 or syndros or tetranabinex).tw,kf. (23408)

8 (deltanyne or "abbott 40566" or namisol or dronabinolum or "QCD 84924" or "CCRIS 4726").tw,kf. (22)

9 or/1-8 [MARIJUANA] (142700)

10 limit 9 to systematic reviews [Limit not valid in AMED,Embase,CDSR,DARE,CLHTA; records were retained] (75932)

11 meta analysis.pt. (92963)

12 exp meta-analysis as topic/ (55036)

13 (meta-analy* or metanaly* or metaanaly* or met analy* or integrative research or integrative review* or integrative overview* or research integration or research overview* or collaborative review*).tw,kf. (357167)

14 (systematic review* or systematic overview* or evidence-based review* or evidence-based overview* or (evidence adj3 (review* or overview*)) or meta-review* or meta-overview* or meta-synthes* or "review of reviews" or technology assessment* or HTA or HTAs).tw,kf. (444798)

15 exp Technology assessment, biomedical/ (23384)

16 (cochrane or health technology assessment or evidence report).jw. (45708)

17 (network adj (MA or MAs)).tw,kf. (15)

18 (NMA or NMAs).tw,kf. (3905)

19 indirect* compar*.tw,kf. (5109)

20 (indirect treatment* adj1 compar*).tw,kf. (559)

21 (mixed treatment* adj1 compar*).tw,kf. (1401)

22 (multiple treatment* adj1 compar*).tw,kf. (359)

23 (multi-treatment* adj1 compar*).tw,kf. (3)

24 simultaneous* compar*.tw,kf. (2359)

25 mixed comparison?.tw,kf. (78)

26 or/11-25 (751129)

27 9 and 26 (2706)

28 10 or 27 [MARIJUANA - REVIEWS] (76481)

29 exp Animals/ not (exp Animals/ and Humans/) (16028624)

30 28 not 29 [ANIMAL-ONLY REMOVED] (42478)

31 (comment or editorial or interview or letter or news or newspaper article).tw,kw. (883908)

32 30 not 31 [OPINION PIECES REMOVED] (40952)

33 32 use ppez [MEDLINE RECORDS] (1059)

34 cannabis/ (43482)

35 exp cannabinoid/ (63182)

36 medical cannabis/ (1541)

37 exp "cannabis use"/ (7145)

38 ("c.indica" or cannabi* or bhang or cannador or charas or eucannabinolide* or ganja or ganjah or hash or hashish or hemp or marihuana* or marijuana*).tw,kw. (112390)

39 (epidiolex or gwp 42003p or gwp42003p or nabidiolex).tw,kw. (79)

40 (dronabinol or thc or trans tetrahydrocannabinol* or tetrahydrocannabinol* or ea 1477 or ea1477 or marinol or qcd 84924 or syndros or tetranabinex).tw,kw. (23676)

41 (deltanyne or "abbott 40566" or namisol or dronabinolum or "QCD 84924" or "CCRIS 4726").tw,kw. (22)

42 or/34-41 [MARIJUANA] (142383)

43 meta-analysis/ (233488)

44 "systematic review"/ (154451)

45 "meta analysis (topic)"/ (37616)

46 (meta-analy* or metanaly* or metaanaly* or met analy* or integrative research or integrative review* or integrative overview* or research integration or research overview* or collaborative review*).tw,kw. (359782)

47 (systematic review* or systematic overview* or evidence-based review* or evidence-based overview* or (evidence adj3 (review* or overview*)) or meta-review* or meta-overview* or meta-synthes* or "review of reviews" or technology assessment* or HTA or HTAs).tw,kw. (447632)

48 biomedical technology assessment/ (22246)

49 (cochrane or health technology assessment or evidence report).jw. (45708)

50 (network adj (MA or MAs)).tw,kw. (15)

51 (NMA or NMAs).tw,kw. (3924)

52 indirect comparison?.tw,kw. (4771)

53 (indirect treatment* adj1 comparison?).tw,kw. (560)

54 (mixed treatment* adj1 comparison?).tw,kw. (1401)

55 (multiple treatment* adj1 comparison?).tw,kw. (267)

56 (multi-treatment* adj1 comparison?).tw,kw. (3)

57 simultaneous comparison?.tw,kw. (1029)

58 mixed comparison?.tw,kw. (79)

59 or/43-58 (800640)

60 42 and 59 [MARIJUANA - REVIEWS] (3028)

61 exp animal experimentation/ or exp animal model/ or exp animal experiment/ or nonhuman/ or exp vertebrate/ (47893282)

62 exp human/ or exp human experimentation/ or exp human experiment/ (37171841)

63 61 not 62 (10726390)

64 60 not 63 [ANIMAL-ONLY REMOVED] (2975)

65 (editorial or letter).pt. (3052771)

66 64 not 65 [OPINION PIECES REMOVED] (2949)

67 66 use emczd [EMBASE RECORDS] (1538)

68 exp cannabis/ (45988)

69 exp cannabinoids/ (75945)

70 marijuana usage/ (2495)

71 ("c.indica" or cannabi* or bhang or cannador or charas or eucannabinolide* or ganja or ganjah or hash or hashish or hemp or marihuana* or marijuana*).tw. (111050)

72 (epidiolex or gwp 42003p or gwp42003p or nabidiolex).tw. (79)

73 (dronabinol or thc or trans tetrahydrocannabinol* or tetrahydrocannabinol* or ea 1477 or ea1477 or marinol or qcd 84924 or syndros or tetranabinex).tw. (23286)

74 (deltanyne or "abbott 40566" or namisol or dronabinolum or "QCD 84924" or "CCRIS 4726").tw. (22)

75 or/68-74 [MARIJUANA] (141686)

76 meta analysis/ (233488)

77 (meta-analy* or metanaly* or metaanaly* or met analy* or integrative research or integrative review* or integrative overview* or research integration or research overview* or collaborative review*).tw. (356178)

78 (systematic review* or systematic overview* or evidence-based review* or evidence-based overview* or (evidence adj3 (review* or overview*)) or meta-review* or meta-overview* or meta-synthes* or "review of reviews" or technology assessment* or HTA or HTAs).tw. (443487)

79 (network adj (MA or MAs)).tw. (15)

80 (NMA or NMAs).tw. (3895)

81 indirect comparison?.tw. (4708)

82 (indirect treatment* adj1 comparison?).tw. (546)

83 (mixed treatment* adj1 comparison?).tw. (1335)

84 (multiple treatment* adj1 comparison?).tw. (258)

85 (multi-treatment* adj1 comparison?).tw. (3)

86 simultaneous comparison?.tw. (1029)

87 mixed comparison?.tw. (78)

88 or/76-87 (714500)

89 75 and 88 [MARIJUANA - REVIEWS] (2626)

90 exp Animals/ not (exp Animals/ and Humans/) (16028624)

91 89 not 90 [ANIMAL-ONLY REMOVED] (2052)

92 91 use ppez,emczd,amed,coch,dare,clhta (1629)

93 91 not 92 [PSYCINFO RECORDS] (423)

94 exp cannabis/ (45988)

95 cannabinoids/ (20568)

96 ("c.indica" or cannabi* or bhang or cannador or charas or eucannabinolide* or ganja or ganjah or hash or hashish or hemp or marihuana* or marijuana*).tw. (111050)

97 (epidiolex or gwp 42003p or gwp42003p or nabidiolex).tw. (79)

98 (dronabinol or thc or trans tetrahydrocannabinol* or tetrahydrocannabinol* or ea 1477 or ea1477 or marinol or qcd 84924 or syndros or tetranabinex).tw. (23286)

99 (deltanyne or "abbott 40566" or namisol or dronabinolum or "QCD 84924" or "CCRIS 4726").tw. (22)

100 or/94-99 [MARIJUANA] (133237)

101 meta analysis/ (233488)

102 (meta-analy* or metanaly* or metaanaly* or met analy* or integrative research or integrative review* or integrative overview* or research integration or research overview* or collaborative review*).tw. (356178)

103 (systematic review* or systematic overview* or evidence-based review* or evidence-based overview* or (evidence adj3 (review* or overview*)) or meta-review* or meta-overview* or meta-synthes* or "review of reviews" or technology assessment* or HTA or HTAs).tw. (443487)

104 (network adj (MA or MAs)).tw. (15)

105 (NMA or NMAs).tw. (3895)

106 indirect comparison?.tw. (4708)

107 (indirect treatment* adj1 comparison?).tw. (546)

108 (mixed treatment* adj1 comparison?).tw. (1335)

109 (multiple treatment* adj1 comparison?).tw. (258)

110 (multi-treatment* adj1 comparison?).tw. (3)

111 simultaneous comparison?.tw. (1029)

112 mixed comparison?.tw. (78)

113 or/101-112 (714500)

114 100 and 113 [MARIJANA - REVIEWS] (2488)

115 114 use amed [AMED RECORDS] (9)

116 Cannabis/ (43482)

117 exp Cannabinoids/ (75945)

118 Medical Marijuana/ (1149)

119 Marijuana Smoking/ (6815)

120 ("c.indica" or cannabi* or bhang or cannador or charas or eucannabinolide* or ganja or ganjah or hash or hashish or hemp or marihuana* or marijuana*).ti,ab,kw. (111748)

121 (epidiolex or gwp 42003p or gwp42003p or nabidiolex).ti,ab,kw. (30)

122 (dronabinol or thc or trans tetrahydrocannabinol* or tetrahydrocannabinol* or ea 1477 or ea1477 or marinol or qcd 84924 or syndros or tetranabinex).ti,ab,kw. (23209)

123 (deltanyne or "abbott 40566" or namisol or dronabinolum or "QCD 84924" or "CCRIS 4726").ti,ab,kw. (13)

124 or/116-123 [MARIJUANA] (142553)

125 124 use coch,dare,clhta [DSR, DARE, HTA RECORDS] (71)

126 33 or 67 or 93 or 115 or 125 [ALL DATABASES] (3100)

127 remove duplicates from 126 (2004) [TOTAL UNIQUE RECORDS]

128 127 use ppez (860) [UNIQUE MEDLINE RECORDS]

129 127 use emczd (860) [UNIQUE EMBASE RECORDS]

130 127 use amed (2) [UNIQUE AMED RECORDS]

131 127 use coch (26) [UNIQUE COCHRANE RECORDS]

132 127 use dare (26) [UNIQUE DARE RECORDS]

133 127 use clhta (19) [UNIQUE HTA RECORDS]

134 127 not (128 or 129 or 130 or 131 or 132 or 133) (211) [UNIQUE PSYCINFO RECORDS]
