# Supplementary material for: Benefits and harms of medical cannabis: a scoping review of systematic reviews
Source: Syst Rev. 2019 Dec 10;8:320. doi: 10.1186/s13643-019-1243-x (PMC6905063; doi:10.1186/s13643-019-1243-x)
Supplement: Supplementary file 3 — Additional file 3. Grey Literature Sources. [file 13643_2019_1243_MOESM3_ESM.docx]

**Appendix 3: Grey Literature Search**

- The Alberta College of Family Physicians
- Alberta Health and Wellness
- Canadian Agency for Drugs and Technologies in Health (CADTH)
- Health Quality Council of Alberta (HQCA)
- Health Quality Ontario
- The Hospital for Sick Children (SickKids)
- Institut national d’excellence en santé et en services sociaux (INESSS)
- Institute of Health Economics (IHE)
- Manitoba Centre for Health Policy (MCHP)
- McGill University Health Centre (MUHC)
- NLCAHR: Newfoundland and Labrador Centre for Applied Health Research. Contextualized Health Research Synthesis Program (CHRSP)
- Ottawa Hospital Research Institute (OHRI)
- Pan-Canadian HTA Collaborative
- Program for Assessment of Technology in Health (PATH)
- Therapeutics Initiative.
- International Network of Agencies for Health Technology Assessment (INAHTA)
- WHO
- Australian Government: Department of Health and Ageing
- Australian Government Department of Health and Ageing. Medical Services Advisory Committee (MSAC)
- Joanna Briggs Institute (JBI)
- TRIP database
- Queensland Government: Health Policy Advisory Committee on Technology (HealthPACT)
- Royal Australasian College of Surgeons
- Institute of Technology Assessment
- [Ludwig Boltzmann Institut für Health Technology Assessment](http://hta.lbg.ac.at/page/about-us) (LBI)
- Belgian Health Care Knowledge Centre (KCE)
- [Sundhedsstyrelsen. Danish Health and Medicines Authority](http://sundhedsstyrelsen.dk/en/about-us) (DHMA)
- Comite d’Evaluation de Diffusion des Innovations Technologiques
- [Haute Autorité de santé/ French National Authority for Health](http://www.has-sante.fr/portail/display.jsp?id=c_5443&pcid=c_5443) (HAS)
- [Health Information and Quality Authority](http://www.hiqa.ie/about-us).
- [Health Service Executive](http://www.lenus.ie/hse/pages/About%20Lenus.html). Irish Health Repository **(**Lenus**)**
- [De Gezondheidsraad](http://www.gezondheidsraad.nl/en/about-us) (GR). Health Council of the Netherlands
- [Zorginstituut Nederland](https://www.zorginstituutnederland.nl/). National Health Care Institute Netherlands
- [Nasjonalt kunnskapssenter for helsetjenesten. Norwegian Knowledge Centre for the Health Services](http://www.kunnskapssenteret.no/en/frontpage).
- [Agencia de Evaluación de Tecnologías Sanitarias, Instituto de Salud “Carlos III”](http://www.eng.isciii.es/ISCIII/es/contenidos/fd-el-instituto/quienes-somos.shtml). Institute of Health Carlos III
- [Agència de Qualitat i Avaluació Sanitàries de Catalunya](http://aquas.gencat.cat/ca/sobre_aquas/) (AQuAS). Agency for Health Quality and Assessment of Catalonia
- Sahlgrenska University Hospital
- [Swedish Council on Health Technology Assessment](http://www.sbu.se/en/About-SBU) (SBU).
- Healthcare Improvement Scotland
- [National Institute for Health and Care Excellence](http://www.nice.org.uk/aboutnice/) (NICE)
- [National Institute for Health Research. Horizon Scanning Centre](http://www.hsc.nihr.ac.uk/about-us/) (NHSC)
- NHS Purchasing and Supply Agency: Centre for Evidence-based Purchasing
- NIHR Evaluation, Trials, and Studies Coordinating Centre
- UK Department of Health. International Resource for Infection Control (iNRIC)
- National Health Service UK (NHS.)
- Agency for Healthcare Research and Quality (AHRQ)
- [Blue Cross and Blue Shield Association](http://www.bcbs.com/blueresources/tec/)**.** Technology Evaluation Center (TEC)
- Centers for Medicare and Medicaid Services
- ECRI Institute
- Institute for Clinical and Economic Review (ICER)
- Washington State Health Care Authority (HCA)
- [Alberta Medical Association](http://www.topalbertadoctors.org/about-top/)
- British Columbia Ministry of Health: BC Guidelines
- [Canadian Medical Association](https://www.cma.ca/En/Pages/history-mission-vision.aspx) (CMA). CMA Infobase: Clinical Practice Guidelines
- Canadian Partnership Against Cancer Corporation: Cancer Guidelines Resource Centre
- Canadian Standards Association
- [The College of Physicians and Surgeons of Ontario](http://www.cpso.on.ca/About-Us) (CPSO). CPGs & Other Guidelines
- [Ontario Association of Medical Laboratories](http://www.oaml.com/about.html) (OAML)
- [Public Health Agency of Canada](http://www.phac-aspc.gc.ca/about_apropos/index-eng.php) (PHAC)
- Registered Nurses’ Association of Ontario (RNAO.) Nursing Best Practice Guidelines
- [University of Ottawa. School of Rehabilitation Science](http://www.health.uottawa.ca/rehabguidelines/en/login.php). Evidence-based Practice
- [Winnipeg Regional Health Authority](http://www.wrha.mb.ca/about/aboutus.php) (WRHA). Evidence Informed Practice Tools
- Academy of Medicine of Malaysia
- Aetna Inc
- American Association of Clinical Chemistry
- Best Practice Advocacy Centre New Zealand
- Centres for Disease Control and Prevention
- Guidelines and Audit Implementation Network (GAIN)
- French National Authority for Health (HAS)
- Institute for Clinical Systems Improvement (ICSI)
- National Guideline Clearinghouse (NGC)
- National Health and Medical Research Council (NHMRC)
- Scottish Intercollegiate Guidelines Network (SIGN)
- [McMaster University, McMaster Health Forum](https://www.healthsystemsevidence.org/about?). Health Systems Evidence
- [Centre for Reviews and Dissemination](http://www.york.ac.uk/crd/about/). HTA Database
- [SAMHSA - Substance Abuse and Mental Health Services Administration](https://www.samhsa.gov/)
- Canadian Centre on Substance Abuse and Addiction (CCSA)
