# Supplementary material for: Benefits and harms of medical cannabis: a scoping review of systematic reviews
Source: Syst Rev. 2019 Dec 10;8:320. doi: 10.1186/s13643-019-1243-x (PMC6905063; doi:10.1186/s13643-019-1243-x)
Supplement: Supplementary file 4 — Additional file 4. Listing of Data Extraction Items. [file 13643_2019_1243_MOESM4_ESM.docx]

**Appendix 4: Data extraction items**

| - Type of report - Journal Name - First author (last name, initial) - Year - Country of Corresponding Author - Country of First Author Funding - Objective of SR - Intent of SR - Databases Searched - Total # of databases searched - Search dates - Selection criteria - Protocol reported - How was medical marijuana use defined? - Where was MM use defined? - Type of cannabis - Type of cannabis - comment - Comparator (as defined in methods) - ICD-10 - Specific Conditions/illnesses - Setting defined | - Population - Sex - Special populations (e.g., pregnancy) - Indication for prescription - Outcomes specified a priori? - Outcomes defined? - Quality assessment tool used - Total # studies on medical cannabis - Total # participants for included cannabis studies - Range of participants for included cannabis studies - Study designs included - Various study designs (list) - Populations in included studies - Type of analysis - Type of analysis (comment) - Illness/condition (as relates to following outcomes) | - Intervention - Comparator - Pain taxonomy (if applicable to this outcome) - Other pain (list) - Results for pain outcomes - Other outcomes reported (list per row) - Results for other outcomes - Comments - General conclusions - Strengths and limitations of review - Serious adverse events? - Minor adverse events? - Types of adverse events - Adverse events: additional comments |
| --- | --- | --- |
