# Supplementary material for: Benefits and harms of medical cannabis: a scoping review of systematic reviews
Source: Syst Rev. 2019 Dec 10;8:320. doi: 10.1186/s13643-019-1243-x (PMC6905063; doi:10.1186/s13643-019-1243-x)
Supplement: Supplementary file 6 — Additional file 6. Listing of Studies Excluded During Full Text Screening. [file 13643_2019_1243_MOESM6_ESM.docx]

**Appendix 6: List of Studies Excluded at Full Text Review**

Citations of study reports that were excluded during full text review are provided below, categorized by the reason for exclusion.

**OTHER STUDY DESIGNS**

Position paper: access to therapeutic cannabis. National Nurses Society on Addictions. Perspect Addict Nurs 1995;6(3):3-4.

Marijuana: federal smoke clears, a little. CMAJ 2001;164(10):1397-9.

Herbal medicine. Focus Altern Complement Ther 2005;10(3):222-41.

For neuropathic pain, optimize opioids. J Supportive Oncol 2006;4(2):95-6.

Cannabis derivatives and pain. Prescrire Int 2009;18(103):226.

Adverse effects of cannabis: Inform psychologically vulnerable patients of the risk of serious, dose-dependent disorders. Prescrire Int 2011;20(112):18-23.

What place for cannabis extract in MS? Drug Ther Bull 2012;50(12):141-4.

Erratum: Cannabinoids for medical use: A systematic review and meta-analysis (JAMA - Journal of the American Medical Association (2015) 313:24 (2456-2473)). JAMA - Journal of the American Medical Association 2015;314(21):2308.

Nausea as a symptom cluster. Clin Adv Hematol Oncol 2016;14(8 Supplement 10):18-9.

Committee Opinion Summary NO. 722: Marijuana Use During Pregnancy and Lactation. Obstet Gynecol 2017;130(4):931-2.

Ablin JN, Hauser W. Fibromyalgia syndrome: Novel therapeutic targets. Pain manag 2016;6(4):371-81.

Abrams DI, Trafton J. Is marijuana medicinal? Oncol Rep 2012;(APRIL):10.

Abrams DI. Using medical cannabis in an oncology practice. ONCOLOGY 2016;30(5).

Addington J, Case N, Saleem MM, Auther AM, Cornblatt BA, Cadenhead KS. Substance use in clinical high risk for psychosis: a review of the literature. Early Interv Psychiatry 2014;8(2):104-12.

Aigner M, Treasure J, Kaye W, Kasper S, WFSBP Task Force On Eating Disorders. World Federation of Societies of Biological Psychiatry (WFSBP) guidelines for the pharmacological treatment of eating disorders. World J Biol Psychiatry 2011;12(6):400-43.

Albert RH. End-of-life care: Managing common symptoms. Am Fam Phys 2017;95(6):356-61.

Alberta Heritage Foundation for Medical Research. Use of cannabis or cannabinoids for non-malignant chronic pain (Structured abstract). Health Technology Assessment Database 2016;(2016 Issue 4).

Alderson HL, Lawrie SM. Does cigarette smoking cause psychosis? The Lancet Psychiatry 2015;2(8):672-3.

Alemzadeh-Ansari MJ, Ansari-Ramandi MM, Naderi N. Chronic pain in chronic heart failure: A review article. J Tehran Uni Heart Cent 2017;12(2):49-56.

Alexander CN, Robinson P, Rainforth M. Treating and preventing alcohol, nicotine, and drug abuse through transcendental meditation: A review and statistical meta-analysis. Alcohol Treat Q 1994;11(1-2):13-87.

Alharbi GS, Chen L-C, Knaggs R. Efficacy of anticonvulsant, antidepressant and opioid in treating neuropathic pain - A systematic review and meta-analysis. Pharmacoepidemiol Drug Saf 2016;25:582.

All Wales Medicines Strategy Group (. Delta-9-tetrahydrocannabinol/cannabidiol (SativexReg.) (Structured abstract). Health Technology Assessment Database 2016;(2016 Issue 4).

Ananth P, Reed-Weston A, Wolfe J. Medical marijuana in pediatric oncology: A review of the evidence and implications for practice. Pediatr Blood Cancer 2017.

Andrade C. Cannabis and neuropsychiatry, 1: benefits and risks. J Clin Psychiatry 2016;77(5):e551-e554.

Andre A, Gonthier MP. The endocannabinoid system: its roles in energy balance and potential as a target for obesity treatment. Int J Biochem Cell Biol 2010;42(11):1788-801.

Andreae MH, Sacks H, Carter G, Indyk D, Suslov K, DiMaggio C, Hall C, Johnson M. Bayesian meta regression of cannabis for chronic neuropathy. Clin Transl Sci 2013;6(2):105.

Andrzejewski K, Barbano R, Mink J. Cannabinoids in the treatment of movement disorders: A systematic review of case series and clinical trials. Basal Ganglia 2016;6(3):173-81.

Anthony JC, Degenhardt L. Projecting the impact of changes in cannabis use upon schizophrenia in England and Wales: the role of assumptions and balance in framing an evidence-based cannabis policy. Addiction 2007;102(4):515-8.

Anthony JC, Lopez-Quintero C, Alshaarawy O. Cannabis Epidemiology: A Selective Review. Curr Pharm Des 2017;22(42):6340-52.

Arendt M. Review: current evidence does not show a strong causal relation between the use of cannabis in young people and psychosocial harm. Evid Based Ment Health 2004;7(4):119.

Armentano P. Cannabis and psychomotor performance: a rational review of the evidence and implications for public policy. Drug Test Anal 2013;5(1):52-6.

Ashton CH. Biomedical benefits of cannabinoids? Addict Biol 1999;4(2):111-26.

Ashton CH, Moore PB, Gallagher P, Young AH. Cannabinoids in bipolar affective disorder: a review and discussion of their therapeutic potential. J Psychopharmacol 2005;19(3):293-300.

Ashton J, Hancox RJ. The case for cannabinoid CB1 receptors as a target for bronchodilator therapy for beta-agonist resistant asthma. Curr Drug Targets 2017.

Attal N, Mazaltarine G, Perrouin-Verbe B, Albert T, SOFMER French Society for Physical Medicine and Rehabilitation. Chronic neuropathic pain management in spinal cord injury patients. What is the efficacy of pharmacological treatments with a general mode of administration? (oral, transdermal, intravenous). Ann Phys Rehabil Med 2009;52(2):124-41.

Attal N, Bouhassira D. Pharmacotherapy of neuropathic pain: Which drugs, which treatment algorithms? Pain 2015;156(4 Supplement 1):S104-S114.

Attal N. Symptomatic therapy in ATTR amyloidosis: Pain killers in TTR-FAP. Orphanet J Rare Dis 2015;10.

Baastrup C, Finnerup NB. Pharmacological management of neuropathic pain following spinal cord injury. CNS drugs 2008;22(6):455-75.

Babson KA, Sottile J, Morabito D. Cannabis, Cannabinoids, and Sleep: a Review of the Literature. Curr Psychiatry Rep 2017;19(4):23.

Badowski ME. A review of oral cannabinoids and medical marijuana for the treatment of chemotherapy-induced nausea and vomiting: a focus on pharmacokinetic variability and pharmacodynamics. Cancer Chemother Pharmacol 2017;80(3):441-9.

Bahji A, Mazhar MN. Treatment of cannabis dependence with synthetic cannabinoids: A systematic review. Can J Addict 2016;7(4):8-13.

Baldwin DS. Pharmacological treatment of social phobia: Efficacy, acceptability and unmet needs. Eur Neuropsychopharmacol 2012;22:S132-S133.

Bally N, Zullino D, Aubry J-M. Cannabis use and first manic episode. J Affect Disord 2014;165:103-8.

Balog DL, Epstein ME, Amodio-Groton MI. HIV wasting syndrome: treatment update. Ann Pharmacother 1998;32(4):446-58.

Bandelow B. Generalized anxiety disorder and pain. Mod Trends Pharmacopsychiatry 2015;30:153-65.

Bao Y, Kong X, Yang L, Liu R, Shi Z, Li W, Hua B, Hou W. Complementary and alternative medicine for cancer pain: an overview of systematic reviews. Evid Based Complement Alternat Med 2014;2014:170396.

Barbetta C, Currow DC, Johnson MJ. Non-opioid medications for the relief of chronic breathlessness: current evidence. Expert Rev Respir Med 2017;11(4):333-41.

Barkin JA, Nemeth Z, Saluja AK, Barkin JS. Cannabis-Induced Acute Pancreatitis: A Systematic Review. Pancreas 2017;46(8):1035-8.

Barnes J. Systematic review of cannabinoids for treatment of pain. Focus Altern Complement Ther 2001;6(4):256-7.

Barnes J. Systematic review of cannabinoids for cancer chemotherapy-induced nausea and vomiting. Focus Altern Complement Ther 2002;7(2):144-5.

Baron EP. Comprehensive review of medicinal marijuana, cannabinoids, and therapeutic implications in medicine and headache: What a long strange trip it's been . Headache 2015;55(6):885-916.

Baumrucker SJ. Medical marijuana. Am J Hosp Palliat Care 2001;18(4):227-8.

Beal BR, Wallace MS. An Overview of Pharmacologic Management of Chronic Pain. Med Clin North Am 2016;100(1):65-79.

Beaulicu P. Non-opioid strategies for acute pain management. Can J Anesth 2007;54(6):481-5.

Beaulieu S, Saury S, Sareen J, Tremblay J, Schutz CG, McIntyre RS, Schaffer A, Canadian Network for Mood and Anxiety Treatments (CANMAT) Task Force. The Canadian Network for Mood and Anxiety Treatments (CANMAT) task force recommendations for the management of patients with mood disorders and comorbid substance use disorders. Ann Clin Psychiatry 2012;24(1):38-55.

Beg S, Swain S, Hasan H, Barkat MA, Hussain MS. Systematic review of herbals as potential anti-inflammatory agents: Recent advances, current clinical status and future perspectives. pharmacogn Rev 2011;5(10):120-37.

Bega D, Gonzalez-Latapi P, Zadikoff C, Simuni T. A Review of the Clinical Evidence for Complementary and Alternative Therapies in Parkinson's Disease. Curr Treat Options Neurol 2014;16(10).

Bell H. The Intractable Pain conundrum. Minn Med 2016;99(4):12-7.

Bell JA, Doege TC. Athletes' Use and Abuse of Drugs. Phys Sportsmed 1987;15(3):99-108.

Ben Amar M. Cannabinoids in medicine: A review of their therapeutic potential. J Ethnopharmacol 2006;105(1-2):1-25.

Beniczky S, Tajti J, Timea VE, Vecsei L. Evidence-based pharmacological treatment of neuropathic pain syndromes. J Neural Transm 2005;112(6):735-49.

Bethoux F. Spasticity Management After Stroke. Phys Med Rehabil Clin North Am 2015;26(4):625-39.

Bettin P, Di MF. Glaucoma: Present challenges and future trends. Ophthalmic Res 2013;50(4):197-208.

Boccia RV. Chemotherapy-induced nausea and vomiting: Identifying and addressing unmet needs. J Clin Outcomes Manage 2013;20(8):377-84.

Bonino JA, McCallum RW. Pharmacological management of chronic gastroparesis. Pract Gastroenterol 2007;31(6):44-59.

Bortolato M, Bini V, Tambaro S. Vulnerability Factors for the Psychiatric and Behavioral Effects of Cannabis. Pharmaceuticals (Basel) 2010;3(9):2799-820.

Bostwick JM. Blurred boundaries: the therapeutics and politics of medical marijuana. Mayo Clin Proc 2012;87(2):172-86.

Boychuk DG, Goddard G, Mauro G, Orellana MF. The effectiveness of cannabinoids in the management of chronic nonmalignant neuropathic pain: a systematic review. J Oral Facial Pain Headache 2015;29(1):7-14.

Braun I. Systematic review of marijuana's medicinal aspects. Psycho-Oncology 2015;24:253-4.

Brownjohn PW, Ashton JC. Novel targets in pain research: The case for CB2 receptors as a biorational pain target. Curr Anaesth Crit Care 2009;20(5-6):198-203.

Broyd SJ, van Hell HH, Beale C, Yucel M, Solowij N. Acute and Chronic Effects of Cannabinoids on Human Cognition-A Systematic Review. Biol Psychiatry 2016;79(7):557-67.

Bumb JM, Enning F, Leweke FM. Drug repurposing and emerging adjunctive treatments for schizophrenia. Expert Opin Pharmacother 2015;16(7):1049-67.

Bunt G. Marijuana is not good medicine. PAIN MED 2013;14(6):799.

Burch J, McKenna C, Palmer S, Norman G, Glanville J, Sculpher M, Woolacott N. Rimonabant for the treatment of overweight and obese people (Structured abstract). Health Technology Assessment Database 2016;(2016 Issue 4).

Burns JK. Pathways from cannabis to psychosis: a review of the evidence. Front Psychiatr 2013;4:128.

Burns TL, Ineck JR. Cannabinoid analgesia as a potential new therapeutic option in the treatment of chronic pain. Ann Pharmacother 2006;40(2):251-60.

CADTH. Medical marijuana for the treatment of mental illness: clinical evidence (Structured abstract). Health Technology Assessment Database 2016;(2016 Issue 4).

CADTH. Nabilone for chronic pain management: a review of clinical effectiveness, safety, and guidelines (Structured abstract). Health Technology Assessment Database 2016;(2016 Issue 4).

CADTH. The use of medical marijuana: guidelines and recommendations (Structured abstract). Health Technology Assessment Database 2016;(2016 Issue 4).

Campbell RL, Germain A. Nightmares and Posttraumatic Stress Disorder (PTSD). Curr Sleep Med Rep 2016;2(2):74-80.

Canadian Agency for Drugs and Technologies in Health. Cannabinoids as co-analgesics: review of clinical effectiveness (Structured abstract). Health Technology Assessment Database 2016;(2016 Issue 4).

Canadian Agency for Drugs and Technologies in Health. Cannabinoids for the management of neuropathic pain: review of clinical effectiveness (Structured abstract). Health Technology Assessment Database 2016;(2016 Issue 4).

Carey MP, Burish TG, Brenner DE. Delta-9-tetrahydrocannabinol in cancer chemotherapy: research problems and issues. Ann Intern Med 1983;99(1):106-14.

Carrillo MR, Jennings GE, Le C, Dong A, Devineni D, Sahafi R, Goo H, Afghani B. Use of medical marijuana for treatment of pediatric patients with epilepsy. J Invest Med 2016;64(1):200-1.

Carter GT, Rosen BS. Marijuana in the management of amyotrophic lateral sclerosis. Am J Hosp Palliat Care 2001;18(4):264-70.

Carter GT. The argument for medical marijuana for the treatment of chronic pain. PAIN MED 2013;14(6):800.

Carter GT, Flanagan AM, Earleywine M, Abrams DI, Aggarwal SK, Grinspoon L. Cannabis in palliative medicine: Improving care and reducing opioid-related morbidity. American Journal of Hospice & Palliative Medicine 2011;28(5):297-303.

Centre for Reviews and Dissemination. A meta-analytic review of school-based prevention for cannabis use (Structured abstract). Database of Abstracts of Reviews of Effects 2015;(2).

Centre for Reviews and Dissemination. Appetite stimulants in cystic fibrosis: a systematic review (Structured abstract). Database of Abstracts of Reviews of Effects 2015;(2).

Centre for Reviews and Dissemination. Assessment of cannabis use disorders: a systematic review of screening and diagnostic instruments (Provisional abstract). Database of Abstracts of Reviews of Effects 2015;(2).

Centre for Reviews and Dissemination. Behavioral therapies for treatment-seeking cannabis users: a meta-analysis of randomized controlled trials (Provisional abstract). Database of Abstracts of Reviews of Effects 2015;(2).

Centre for Reviews and Dissemination. Cannabis for therapeutic purposes and public health and safety: a systematic and critical review (Provisional abstract). Database of Abstracts of Reviews of Effects 2015;(2).

Centre for Reviews and Dissemination. Short scales to assess cannabis-related problems: a review of psychometric properties (Structured abstract). Database of Abstracts of Reviews of Effects 2015;(2).

Chang F-Y. Irritable bowel syndrome: The evolution of multi-dimensional looking and multidisciplinary treatments. World J Gastroenterol 2014;20(10):2499-514.

Cherny NI. Taking care of the terminally ill cancer patient: Management of gastrointestinal symptoms in patients with advanced cancer. Ann Oncol 2004;15(SUPPL. 4).

Chetty S, Baalbergen E, Bhigjee AI, Kamerman P, Ouma J, Raath R, Raff M, Salduker S. Clinical practice guidelines for management of neuropathic pain: Expert panel recommendations for South Africa. S Afr Fam Pract 2013;55(2):143-58.

Chinuck RS, Fortnum H, Baldwin DR. Appetite stimulants in cystic fibrosis: a systematic review. J Hum Nutr Diet 2007;20(6):526-37.

Cinti S. Medical marijuana in HIV-positive patients: what do we know? J Int Assoc Physicians AIDS Care (Chic Ill) 2009;8(6):342-6.

Cocchetto DM, Cook LF, Cato AE. A critical review of the safety and antiemetic efficacy of delta-9-tetrahydrocannabinol. Drug Intell Clin Pharm 1981;15(11):867-75.

Cohen PJ. Medical marijuana: the conflict between scientific evidence and political ideology. Part two of two. J Pain Pall Care Pharmacother 2009;23(2):120-40.

Colombo B, Annovazzi POL, Comi G. Medications for neuropathic pain: Current trends. Neurol Sci 2006;27(SUPPL. 2):s183-s189.

Comeau P. New dosage limits for medical marijuana: but where's the science? CMAJ 2007;177(6):556-7.

Cook JL, Green C, de la Ronde S, Dell CA, Graves L, Ordean A, Ruiter J, Steeves M, Wong S. Epidemiology and Effects of Substance Use in Pregnancy. J Obstet Gynaecol Can 2017;39(10):906-15.

Copeland J. The glass ceiling on evidence of cannabis related harms - flawed or just false? Addiction 2011;106(2):249-2.

Copeland J, Pokorski I. Progress toward pharmacotherapies for cannabis-use disorder: an evidence-based review. Subst abuse rehabil 2016;7:41-53.

Corcoran C, Grinspoon S. Treatments for wasting in patients with the acquired immunodeficiency syndrome. New Engl J Med 1999;340(22):1740-50.

Corliss M, Zhang Y, Broadbent J, Salazar-Grueso E. A meta-analysis of spasticity and pain: Implications for botulinum toxin treatment. Pain Med (USA) 2009;10(1):273.

Cotter J. Efficacy of Crude Marijuana and Synthetic Delta-9-Tetrahydrocannabinol as Treatment for Chemotherapy-Induced Nausea and Vomiting: A Systematic Literature Review. Oncol Nurs Forum 2009;36(3):345-52.

Coulston CM, Perdices M, Tennant CC. The neuropsychology of cannabis and other substance use in schizophrenia: review of the literature and critical evaluation of methodological issues. Aust N Z J Psychiatry 2007;41(11):869-84.

Cridge BJ, Rosengren RJ. Critical appraisal of the potential use of cannabinoids in cancer management. Cancer Manag Res 2013;5:301-13.

Crippa JA, Zuardi AW, Martin-Santos R, Bhattacharyya S, Atakan Z, McGuire P, Fusar-Poli P. Cannabis and anxiety: a critical review of the evidence. HUM PSYCHOPHARMACOL 2009;24(7):515-23.

Croxford JL, Miller SD. Towards cannabis and cannabinoid treatment of multiple sclerosis. Drugs Today 2004;40(8):663-76.

Cuba LF, Salum FG, Cherubini K, Figueiredo MAZ. Cannabidiol: an alternative therapeutic agent for oral mucositis? J Clin Pharm Ther 2017;42(3):245-50.

Curatolo M, Bogduk N. Pharmacologic pain treatment of musculoskeletal disorders: current perspectives and future prospects. Clin J Pain 2001;17(1):25-32.

Dalacorte RR, Rigo JC, Dalacorte A. Pain management in the elderly at the end of life. North Am J Med Sci 2011;3(8):348-54.

Dantas FG. Epilepsy and marijuana - A review. J Epilepsy Clin Neurophysiol 2005;11(2):91-3.

Davis M. Nausea and vomiting of pregnancy: An evidence-based review. J Perinat Neonat Nurs 2004;18(4):312-28.

Davis M, Goforth H. Cannabinoids: Now and in the future. J Pain Symptom Manage 2015;49(2):364.

Davis MP. Systematic review of adverse effects of medical cannabinoids. J Pain Palliative Care Pharmacother 2008;22(4):316-7.

Davis MP. Oral nabilone capsules in the treatment of chemotherapy-induced nausea and vomiting and pain. Expert Opin Investig Drugs 2008;17(1):85-95.

De Marqui ABT. Evaluation of endometriosis-associated pain and influence of conventional treatment: A systematic review. Rev Assoc Med Bras 2015;61(6):507-18.

De Sa JCC, Airas L, Bartholome E, Grigoriadis N, Mattle H, Oreja-Guevara C, O'Riordan J, Sellebjerg F, Stankoff B, Vass K, Walczak A, Wiendl H, Kieseier BC. Symptomatic therapy in multiple sclerosis: A review for a multimodal approach in clinical practice. Ther Adv Neurol Disord 2011;4(3):139-68.

Denis C, Lavie E, Fatseas M, Auriacombe M. WITHDRAWN: Psychotherapeutic interventions for cannabis abuse and/or dependence in outpatient settings. Cochrane Database Syst Rev 2013;(6):CD005336.

Diehl V, Marty M. Efficacy and safety of antiemetics. CANCER TREAT REV 1994;20(4):379-92.

DiVall MV, Cersosimo RJ. Prevention and treatment of chemotherapy-induced nausea and vomiting: A review. Formulary 2007;42(6):378-88.

Dong J, Cui Y, Li S, Le W. Current pharmaceutical treatments and alternative therapies of parkinson's disease. Curr Neuropharmacol 2016;14(4):339-55.

Donoghue K, Doody GA. Effect of illegal substance use on cognitive function in individuals with a psychotic disorder, a review and meta-analysis. Neuropsychology 2012;26(6):785-801.

dos Santos RG, Hallak JEC, Leite JP, Zuardi AW, Crippa JAS. Phytocannabinoids and epilepsy. J Clin Pharm Ther 2015;40(2):135-43.

Dosenovic S, Jelicic Kadic A, Miljanovic M, Biocic M, Boric K, Cavar M, Markovina N, Vucic K, Puljak L. Interventions for Neuropathic Pain: An Overview of Systematic Reviews. Anesth Analg 2017;125(2):643-52.

Downer EJ. Cannabinoids and innate immunity: taking a toll on neuroinflammation. ScientificWorldJournal 2011;11:855-65.

Drum PA. How marijuana promoters bypass the law - And the public good. Drug Topics 2015;159(12).

Dy SM, Apostol CC. Evidence-based approaches to other symptoms in advanced cancer. Cancer J 2010;16(5):507-13.

Eisenstein TK, Meissler JJ. Effects of Cannabinoids on T-cell Function and Resistance to Infection. J Neuroimmune Pharmacol 2015;10(2):204-16.

El-Alfy AT, Abourashed EA, Matsumoto RR. Nature against depression. Curr Med Chem 2012;19(14):2229-41.

Elmariah SB. Adjunctive Management of Itch in Atopic Dermatitis. Dermatol Clin 2017;35(3):373-94.

Engh JA, Bramness JG. Psychosis relapse, medication non-adherence, and cannabis. The Lancet Psychiatry 2017;4(8):578-9.

English DR, Hulse GK, Milne E, Holman CD, Bower CI. Maternal cannabis use and birth weight: a meta-analysis. Addiction 1997;92(11):1553-60.

Ernst E. Herbal Medicine in the Treatment of Rheumatic Diseases. Rheum Dis Clin North Am 2011;37(1):95-102.

Ettinger DS, Bierman PJ, Bradbury B, Comish CC, Ellis G, Ignoffo RJ, Kirkegaard S, Kloth DD, Kris MG, Lim D, Markiewicz MA, McNulty R, Nabati L, Todaro B, Urba S, Yowell S. Antiemesis: Clinical practice guidelines in OncologyTM. JNCCN J Nat Compr Cancer Netw 2007;5(1):12-33.

Ettinger DS, Armstrong DK, Barbour S, Berger MJ, Bierman PJ, Bradbury B, Ellis G, Kirkegaard S, Kloth DD, Kris MG, Lim D, Michaud LB, Nabati L, Noonan K, Rugo HS, Siler D, Sorscher SM, Stelts S, Stucky-Marshall L, Todaro B, Urba SG. Antiemesis. JNCCN J Nat Compr Cancer Netw 2012;10(4):456-85.

Farquhar-Smith WP. Do cannabinoids have a role in cancer pain management? Curr opin support palliat care 2009;3(1):7-13.

Farrell M, Buchbinder R, Hall W. Should doctors prescribe cannabinoids? BMJ 2014;348:g2737.

Farzaei MH, Shahpiri Z, Bahramsoltani R, Nia MM, Najafi F, Rahimi R. Efficacy and Tolerability of Phytomedicines in Multiple Sclerosis Patients: A Review. CNS drugs 2017;31(10):867-89.

Fife TD, Moawad H, Moschonas C, Shepard K, Hammond N. Clinical perspectives on medical marijuana (cannabis) for neurologic disorders. Neurol Clin Pract 2015;5(4):344-51.

Filloux FM. Cannabinoids for pediatric epilepsy? Up in smoke or real science? Transl pediatr 2015;4(4):271-82.

Fischer B, Jeffries V, Hall W, Room R, Goldner E, Rehm J. Lower Risk Cannabis use Guidelines for Canada (LRCUG): a narrative review of evidence and recommendations. Can J Public Health 2011;102(5):324-7.

Fischer B, Murphy Y, Kurdyak P, Goldner E, Rehm J. Medical marijuana programs - Why might they matter for public health and why should we better understand their impacts? Prev Med Rep 2015;2:53-6.

Fisher B, Johnston D, Leake P. Marijuana for medicinal purposes: an evidence-based assessment (Structured abstract). Health Technology Assessment Database 2016;(2016 Issue 4).

Flament MF, Bissada H, Spettigue W. Evidence-based pharmacotherapy of eating disorders. Int J Neuropsychopharmacol 2012;15(2):189-207.

Forray A. Substance use during pregnancy. F1000Res 2016;5.

Fowler CJ. Plant-derived, synthetic and endogenous cannabinoids as neuroprotective agents. Non-psychoactive cannabinoids, 'entourage' compounds and inhibitors of N-acyl ethanolamine breakdown as therapeutic strategies to avoid pyschotropic effects. Brain Res Brain Res Rev 2003;41(1):26-43.

Frank GKW, Shott ME. The Role of Psychotropic Medications in the Management of Anorexia Nervosa: Rationale, Evidence and Future Prospects. CNS drugs 2016;30(5):419-42.

Friedman D, Devinsky O. Cannabinoids in the treatment of epilepsy. New Engl J Med 2015;373(11):1048-58.

Friedrichsdorf S. Management of distressing non-pain symptoms in pediatric end-of-life care. J Pain Symptom Manage 2012;43(2):332-3.

Gadde KM, Allison DB. Cannabinoid-1 receptor antagonist, rimonabant, for management of obesity and related risks. Circulation 2006;114(9):974-84.

Gaertner J, Schiessl C. Cancer Pain Management: What's New? Curr Pain Headache Rep 2013;17(4):1-9.

Gandhi S, Vasisth G, Kapoor A. Systematic review of the potential role of cannabinoids as antiproliferative agents for urological cancers. Can Urol Assoc J 2017;11(3-4):E138-E142.

Garcia K, Rada G. Do cannabinoids have a role to play in Tourettes syndrome? Medwave 2016;16(Suppl5):e6793.

Gevirtz C. Cannabinoids: an emerging role in pain management? Nursing 2009;39(6):59-60.

Gilron I, Dickenson AH. Emerging drugs for neuropathic pain. Expert Opin Emerg Drugs 2014;19(3):329-41.

Godsey J, Grundmann O. Review of Various Herbal Supplements as Complementary Treatments for Oral Cancer. J Diet Suppl 2016;13(5):538-50.

Goldenberg DL, Clauw DJ, Fitzcharles M-A. New Concepts in Pain Research and Pain Management of the Rheumatic Diseases. Semin Arthritis Rheum 2011;41(3):319-34.

Goldenberg M, IsHak WW, Danovitch I. Quality of life and recreational cannabis use. Am J Addict 2017;26(1):8-25.

Gonzalez R, Carey C, Grant I. Nonacute (residual) neuropsychological effects of cannabis use: a qualitative analysis and systematic review. J Clin Pharmacol 2002;42(11 Suppl):48S-57S.

Gorenek B, Pelliccia A, Benjamin EJ, Boriani G, Crijns HJ, Fogel RI, Van Gelder IC, Halle M, Kudaiberdieva G, Lane DA, Bjerregaard LT, Lip GYH, Lochen M-L, Marin F, Niebauer J, Sanders P, Tokgozoglu L, Vos MA, Van Wagoner DR, Fauchier L, Savelieva I, Goette A, Agewall S, Chiang C-E, Figueiredo M, Stiles M, Dickfeld T, Patton K, Piepoli M, Corra U, Manuel Marques-Vidal P, Faggiano P, Schmid J-P, Abreu A. European Heart Rhythm Association (EHRA)/European Association of Cardiovascular Prevention and Rehabilitation (EACPR) position paper on how to prevent atrial fibrillation endorsed by the Heart Rhythm Society (HRS) and Asia Pacific Heart Rhythm Society (APHRS). Eur J Prev Cardiol 2017;24(1):4-40.

Granot R, Day RO, Cohen ML, Murnion B, Garrick R. Targeted pharmacotherapy of evoked phenomena in neuropathic pain: A review of the current evidence. Pain Med (USA) 2007;8(1):48-64.

Green AJ, De-Vries K. Cannabis use in palliative care - an examination of the evidence and the implications for nurses. J Clin Nurs 2010;19(17-18):2454-62.

Grotenhermen F, Muller-Vahl K. The therapeutic potential of cannabis and cannabinoids. Dtsch Arztebl int 2012;109(29-30):495-501.

Gruenbaum SE, Zlotnik A, Gruenbaum BF, Hersey D, Bilotta F. Pharmacologic Neuroprotection for Functional Outcomes After Traumatic Brain Injury: A Systematic Review of the Clinical Literature. CNS drugs 2016;30(9):791-806.

Guindon J, Hohmann AG. The endocannabinoid system and cancer: therapeutic implication. Br J Pharmacol 2011;163(7):1447-63.

Gunderson EW, Haughey HM, Ait-Daoud N, Joshi AS, Hart CL. "Spice" and "K2" herbal highs: a case series and systematic review of the clinical effects and biopsychosocial implications of synthetic cannabinoid use in humans. Am J Addict 2012;21(4):320-6.

Gunn JKL, Rosales CB, Center KE, Nunez AV, Gibson SJ, Ehiri JE. The effects of prenatal cannabis exposure on fetal development and pregnancy outcomes: a protocol. BMJ open 2015;5(3):e007227.

Guy SD, Mehta S, Casalino A, Cote I, Kras-Dupuis A, Moulin DE, Parrent AG, Potter P, Short C, Teasell R, Bradbury CL, Bryce TN, Craven BC, Finnerup NB, Harvey D, Hitzig SL, Lau B, Middleton JW, O'Connell C, Orenczuk S, Siddall PJ, Townson A, Truchon C, Widerstrom-Noga E, Wolfe D, Loh E. The CanPain SCI Clinical Practice Guidelines for Rehabilitation Management of Neuropathic Pain after Spinal Cord: Recommendations for treatment. Spinal Cord 2016;54(S1):S14-S23.

Hall W, Christie M, Currow D. Cannabinoids and cancer: causation, remediation, and palliation. Lancet Oncol 2005;6(1):35-42.

Hamilton GR, Baskett TF. In the arms of Morpheus the development of morphine for postoperative pain relief. Can J Anaesth 2000;47(4):367-74.

Hassell KJ, Ezzati M, Alonso-Alconada D, Hausenloy DJ, Robertson NJ. New horizons for newborn brain protection: Enhancing endogenous neuroprotection. Arch Dis Child Fetal Neonatal Ed 2015;100(6):F541-F551.

Hauser W, Walitt B, Fitzcharles M-A, Sommer C. Review of pharmacological therapies in fibromyalgia syndrome. Arthritis Res Ther 2014;16(1):201.

Hauser W, Petzke F, Fitzcharles MA. Efficacy, tolerability and safety of cannabis-based medicines for chronic pain management - An overview of systematic reviews. Eur J Pain 2017.

Hauser W, Fitzcharles MA, Radbruch L, Petzke F. Cannabinoids in Pain Management and Palliative Medicine. Dtsch Arztebl int 2017;114(38):627-34.

Hay P, Touyz S. Treatment of patients with severe and enduring eating disorders. Curr Opin Psychiatry 2015;28(6):473-7.

Health Council of the Netherlands Gezondheidsraad. Marihuana as medicine (Structured abstract). Health Technology Assessment Database 2016;(2016 Issue 4).

Health TA. Randomised controlled trial of the clinical and cost-effectiveness of a contingency management intervention for reduction of cannabis use and of relapse in early psychosis (Project record). Health Technology Assessment Database 2016;(2016 Issue 4).

Hecht I, Achiron A, Man V, Burgansky-Eliash Z. Modifiable factors in the management of glaucoma: a systematic review of current evidence. Graefe's Arch Clin Exp Ophthalmol 2017;255(4):789-96.

Hempenstall K, Rice ASC. Current treatment options in neuropathic pain. Curr Opin Invest Drugs 2002;3(3):441-8.

Hendren G, Aponte-Feliciano A, Kovac A. Safety and efficacy of commonly used antiemetics. Expert Opin Drug Metab Toxicol 2015;11(11):1753-67.

Henze T, Rieckmann P, Toyka KV. Symptomatic treatment of multiple sclerosis: Multiple Sclerosis Therapy Consensus Group (MSTCG) of the German Multiple Sclerosis Society. Eur Neurol 2006;56(2):78-105.

Hermann D, Schneider M. Potential protective effects of cannabidiol on neuroanatomical alterations in cannabis users and psychosis: a critical review. Curr Pharm Des 2012;18(32):4897-905.

Herrstedt J, Aapro MS, Smyth JF, Del Favero A. Corticosteroids, dopamine antagonists and other drugs. Support Care Cancer 1998;6(3):204-14.

Hill KP. Medical Marijuana for Treatment of Chronic Pain and Other Medical and Psychiatric Problems: A Clinical Review. JAMA 2015;313(24):2474-83.

Hill KP, Palastro MD, Johnson B, Ditre JW. Cannabis and Pain: A Clinical Review. Cannabis Cannabinoid Res 2017;2(1):96-104.

Hill M, Reed K. Pregnancy, breast-feeding, and marijuana: a review article. Obstet Gynecol Surv 2013;68(10):710-8.

Hitzeroth V, Kramer L. Cannabis in South Africa: Back to basics. S Afr J Psychiatry 2014;20(3):99-100.

Hoch E, Bonnetn U, Thomasius R, Ganzer F, Havemann-Reinecke U, Preuss UW. Risks associated with the non-medicinal use of cannabis. Dtsch Arztebl int 2015;112(16):271-8.

Houze B, El-Khatib H, Arbour C. Efficacy, tolerability, and safety of non-pharmacological therapies for chronic pain: An umbrella review on various CAM approaches. Prog Neuropsychopharmacol Biol Psychiatry 2017;79(Pt B):192-205.

Hsieh JTC, Wolfe DL, Connolly S, Townson AF, Curt A, Blackmer J, Sequeira K, Aubut J. Spasticity after spinal cord injury: An evidence-based review of current interventions. Top Spinal Cord Inj Rehabil 2007;13(1):81-97.

Huntley A. A review of the evidence for efficacy of complementary and alternative medicines in MS. Int MS J 2006;13(1):5-4.

Hurd YL, Yoon M, Manini AF, Hernandez S, Olmedo R, Ostman M, Jutras-Aswad D. Early Phase in the Development of Cannabidiol as a Treatment for Addiction: Opioid Relapse Takes Initial Center Stage. Neurother 2015;12(4):807-15.

Hwang JK, Clarke H. Cannabis and pain: A review. J Pain Manage 2016;9(4):395-413.

Hyman SM, Sinha R. Stress-related factors in cannabis use and misuse: Implications for prevention and treatment. J Subst Abuse Treat 2009;36(4):400-13.

Imam A. Evidence level of integrative medicine in supportive care. Asia-Pac J Clin Oncol 2014;10:154.

Inrhaoun H, Kullmann T, Elghissassi I, Mrabti H, Errihani H. Treatment of chemotherapy-induced nausea and vomiting. J Gastrointest Cancer 2012;43(4):541-6.

Irner TB. Substance exposure in utero and developmental consequences in adolescence: a systematic review. CHILD NEUROPSYCHOL 2012;18(6):521-49.

Iseger TA, Bossong MG. A systematic review of the antipsychotic properties of cannabidiol in humans. Schizophr Res 2015;162(1-3):153-61.

Jain NB, Kuhn JE, Murrell WD, Archer KR. What's new in orthopaedic rehabilitation. J Bone Jt Surg Am Vol 2016;98(22):1937-42.

Jankovic J. Medical treatment of dystonia. Mov Disord 2013;28(7):1001-12.

Jaques SC, Kingsbury A, Henshcke P, Chomchai C, Clews S, Falconer J, Abdel-Latif ME, Feller JM, Oei JL. Cannabis, the pregnant woman and her child: weeding out the myths. J Perinatol 2014;34(6):417-24.

Jawahar R, Oh U, Yang S, Lapane KL. A systematic review of pharmacological pain management in multiple sclerosis. Drugs 2013;73(15):1711-22.

Jensen B, Chen J, Furnish T, Wallace M. Medical Marijuana and Chronic Pain: a Review of Basic Science and Clinical Evidence. Curr Pain Headache Rep 2015;19(10):50.

Jordan K, Schmoll HJ, Aapro MS. Comparative activity of antiemetic drugs. Crit Rev Oncol Hematol 2007;61(2):162-75.

Jordan K, Roila F, Molassiotis A, Maranzano E, Clark-Snow RA, Feyer P, MASCC/ESMO. Antiemetics in children receiving chemotherapy. MASCC/ESMO guideline update 2009. Support Care Cancer 2011;19 Suppl 1:S37-S42.

Jouanjus E, Raymond V, Lapeyre-Mestre M, Wolff V. What is the Current Knowledge About the Cardiovascular Risk for Users of Cannabis-Based Products? A Systematic Review. Curr Atheroscler Rep 2017;19(6):26.

Kahan M, Srivastava A. New medical marijuana regulations: the coming storm. CMAJ 2014;186(12):895-6.

Kahan M, Srivastava A, Spithoff S, Bromley L. Prescribing smoked cannabis for chronic noncancer pain: preliminary recommendations. Can Fam Physician 2014;60(12):1083-90.

Kalant H. Smoked marijuana as medicine: not much future. Clin Pharmacol Ther 2008;83(4):517-9.

Kalb C. No green light yet. A long-awaited report supports medical marijuana. So now what? Newsweek 1999;133(13):35.

Kalso E. Cannabinoids for pain and nausea. BMJ 2001;323(7303):2-3.

Kanat O, O'Neil BH. Cancer cachexia: An unmet need in cancer treatment. Clin Invest 2014;4(7):601-4.

Karst M, Wippermann S, Ahrens J. Role of cannabinoids in the treatment of pain and (painful) spasticity. Drugs 2010;70(18):2409-38.

Katchan V, David P, Shoenfeld Y. Cannabinoids and autoimmune diseases: A systematic review. Autoimmun Rev 2016;15(6):513-28.

Katz I, Katz D, Shoenfeld Y, Porat-Katz BS. Clinical evidence for utilizing cannabinoids in the elderly. Isr Med Assoc J 2017;19(2):71-5.

Katzberg HD. Neurogenic muscle cramps. J Neurol 2015;262(8):1814-21.

Kaur G, Andriola M, Manganas L. Efficacy of cannabidiol in children with intractable epilepsy. Neurology 2017;88(16 Supplement 1).

Keeley PW. Nausea and vomiting in people with cancer and other chronic diseases. Clin Evid (Online) 2009;2009.

Kenny C. Mental health. High time for a change? Nurs Times 2001;97(45):12.

Kerbage H, Richa S. Non-antidepressant long-term treatment in post-traumatic stress disorder (Ptsd). Curr Clin Pharmacol 2015;10(2):116-25.

Khoury JM, Neves MdCLd, Roque MAV, Queiroz DAdB, Correa de Freitas AA, de Fatima A, Moreira FA, Garcia FD. Is there a role for cannabidiol in psychiatry? World J Biol Psychiatry 2017;1-16.

Killestein J, Polman C, Uitdehaag MB. Cannabinoids for multiple sclerosis. (10).

Killestein J, Polman CH. Current trials in multiple sclerosis: established evidence and future hopes. Curr Opin Neurol 2005;18(3):253-60.

Kinzbrunner BM. Review: cannabinoids and codeine have similar effects on pain relief, but cannabinoids commonly cause adverse psychotropic effects. ACP J Club 2002;136(1):18.

Kinzbrunner BM. Review: cannabinoids control chemotherapy-induced nausea and vomiting but increase the risk for side effects. ACP J Club 2002;136(1):19.

Kleber HD, Weiss RD, Anton J, George TP, Greenfield SF, Kosten TR, O'Brien CP, Rounsaville BJ, Strain EC, Ziedonis DM, Hennessy G, Connery HS, McIntyre JS, Charles SC, Anzia DJ, Cook IA, Finnerty MT, Johnson BR, Nininger JE, Summergrad P, Woods SM, Yager J. Treatment of patients with substance use disorders: Second edition. Am J Psychiatry 2006;163(8 SUPPL.):1-81.

Knops RRG, Kremer LCM, Verhagen AAE. Paediatric palliative care: Recommendations for treatment of symptoms in the Netherlands Palliative care in other conditions. BMC Palliative Care 2015;14(1):57.

Ko GD, Bober SL, Mindra S, Moreau JM. Medical cannabis - The Canadian perspective. J Pain Res 2016;9:735-44.

Kollas C, Boyer-Kollas B. Medical marijuana: What should palliative care specialists know? (330). J Pain Symptom Manage 2011;41(1):210.

Kooij JS, Bijlenga D. The circadian rhythm in adult attention-deficit/hyperactivity disorder: Current state of affairs. Expert Rev Neurother 2013;13(10):1107-16.

Koppel BS. Cannabis in the Treatment of Dystonia, Dyskinesias, and Tics. Neurotherapeutics 2015;12(4):788-92.

Kovacic K, Di LC. Functional nausea in children. J Pediatr Gastroenterol Nutr 2016;62(3):365-71.

Kraan T, Velthorst E, Koenders L, Zwaart K, Ising HK, van den Berg D, de Haan L, van der Gaag M. Cannabis use and transition to psychosis in individuals at ultra-high risk: review and meta-analysis. Psychol Med 2016;46(4):673-81.

Kramer JL. Medical marijuana for cancer. CA Cancer J Clin 2015;65(2):109-22.

Kung T, Hochman J, Sun ., Bessette L, Haraoui B, Pope J, Bykerk V. Efficacy and safety of cannabinoids for pain in musculoskeletal diseases: A systematic review and meta-analysis. Reumatol Clin Supl 2011;7:166.

Lackner TE. Advances in managing overactive bladder. J Pharm Pract 2000;13(4):277-89.

Lamarine RJ. Marijuana: Modern medical chimaera. J Drug Educ 2012;42(1):1-11.

Laurance J. The medical year: 2010: A nudge in the wrong direction. BMJ 2011;342(7787):22-4.

Le Foll B, Trigo JM, Sharkey KA, Le Strat Y. Cannabis and DELTA9-tetrahydrocannabinol (THC) for weight loss? Med Hypotheses 2013;80(5):564-7.

Lee LA, Chen J, Yin J. Complementary and alternative medicine for gastroparesis. Gastroenterol Clin North Am 2015;44(1):137-50.

Liou GI. Diabetic retinopathy: Role of inflammation and potential therapies for anti-inflammation. World J Diabetes 2010;1(1):12-8.

Liu CS, Chau SA, Ruthirakuhan M, Lanctot KL, Herrmann N. Cannabinoids for the treatment of agitation and aggression in Alzheimer's disease. CNS drugs 2015;29(8):615-23.

Lu J, Gary KW, Neimeier JP, Ward J, Lapane KL. Randomized controlled trials in adult traumatic brain injury. Brain Inj 2012;26(13-14):1523-48.

Lu L, Stein K, Shearer J, Roome C, Lang I, Pearce H. Cost effectiveness of sativex for spasticity in multiple sclerosis. Value Health 2013;16(3):A110.

Lubman DI, Baker A. Cannabis and mental health - management in primary care. Aust Fam Physician 2010;39(8):554-7.

Lynch M. The efficacy of cannabinoids for the treatment of pain: Results of a systematic review. Pain Res Manage 2015;20(3):e34.

Lynch ME, Watson CPN. The pharmacotherapy of chronic pain: A review. Pain Res Manage 2006;11(1):11-38.

Ma J, Wang Y, Wang Z, Li H, Wang Z, Chen G. Neuroprotective Effects of Drug-Induced Therapeutic Hypothermia in Central Nervous System Diseases. Curr Drug Targets 2017;18(12):1392-8.

Maas AIR, Roozenbeek B, Manley GT. Clinical Trials in Traumatic Brain Injury: Past Experience and Current Developments. Neurotherapeutics 2010;7(1):115-26.

Maccoun RJ. The implicit rules of evidence-based policy analysis, updated. Addiction 2010;105(8):1335-6.

Macleod J, Hickman M. Response to commentaries: moving towards an evidence-based policy around cannabis use. Addiction 2010;105(8):1337-9.

Madeddu C, Mantovani G. An update on promising agents for the treatment of cancer cachexia. Curr opin support palliat care 2009;3(4):258-62.

Maher DP, Cohen SP. Medical marijuana research for chronic pain. The Lancet Psychiatry 2017;4(7):513-5.

Mandelbaum DE, de la Monte SM. Adverse Structural and Functional Effects of Marijuana on the Brain: Evidence Reviewed. Pediatr Neurol 2017;66:12-20.

Manseau MW, Goff DC. Cannabinoids and Schizophrenia: Risks and Therapeutic Potential. Neurotherapeutics 2015;12(4):816-24.

Martinotti G, Santacroce R, Papanti D, Elgharably Y, Prilutskaya M, Corazza O. Synthetic Cannabinoids: Psychopharmacology, Clinical Aspects, Psychotic Onset. CNS Neurol Disord Drug Targets 2017;16(5):567-75.

Masdrakis VG, Turic D, Baldwin DS. Pharmacological treatment of social anxiety disorder. Mod Trends Pharmacopsychiatry 2013;29:144-53.

Mather L. Cannabinoid pharmacotherapy: past, present and future. Minerva Anestesiol 2005;71(7-8):405-12.

May MB, Glode AE. Dronabinol for chemotherapy-induced nausea and vomiting unresponsive to antiemetics. Cancer Manag Res 2016;8:49-55.

Mcallister-Williams RH, Baldwin DS, Cantwell R, Easter A, Gilvarry E, Glover V, Green L, Gregoire A, Howard LM, Jones I, Khalifeh H, Lingford-Hughes A, McDonald E, Micali N, Pariante CM, Peters L, Roberts A, Smith NC, Taylor D, Wieck A, Yates LM, Young AH. British Association for Psychopharmacology consensus guidance on the use of psychotropic medication preconception, in pregnancy and postpartum 2017. J Psychopharmacol 2017;31(5):519-52.

McCarberg BH. Cannabinoids:their role in pain and palliation. J Pain Pall Care Pharmacother 2007;21(3):19-28.

McPartland JM, Pruitt PL. Side effects of pharmaceuticals not elicited by comparable herbal medicines: the case of tetrahydrocannabinol and marijuana. Altern Ther Health Med 1999;5(4):57-62.

Mealy NE, Bayes M. Monograph updates of drugs for pain and anesthesia. Drugs Future 2003;28(5):505-24.

Messenger DE, Hallam S, Thomas MG. A systematic review of local excision following neoadjuvant therapy for rectal cancer: Are ypT0 tumours the limit? Gut 2015;64:A346.

Messinger-Rapport BJ, Gammack JK, Thomas DR, Morley JE. Clinical update on nursing home medicine: 2013. J Am Med Dir Assoc 2013;14(12):860-76.

Metts J, Wright S, Sundaram J, Hashemi N. Medical marijuana: A treatment worth trying? J Fam Pract 2016;65(3):178-85.

Metz TD, Stickrath EH. Marijuana use in pregnancy and lactation: a review of the evidence. Am J Obstet Gynecol 2015;213(6):761-78.

Meyer MJ, Megyesi J, Meythaler J, Murie-Fernandez M, Aubut J, Foley N, Katherine S, Bayley M, Marshall S, Teasell R. Acute management of acquired brain injury: An evidence-based review. Can J Neurol Sci 2010;37(3 SUPPL. 1):S36-S37.

Miller G. Pot and pain. Science 2016;354(6312):566-8.

Miller NS, Oberbarnscheidt T. Health policy for marijuana. J Global Drug Policy Pract 2017;11(3).

Minozzi S, Davoli M, Bargagli AM, Amato L, Vecchi S, Perucci CA. An overview of systematic reviews on cannabis and psychosis: discussing apparently conflicting results. Drug Alcohol Rev 2010;29(3):304-17.

Molina PE, Amedee A, LeCapitaine NJ, Zabaleta J, Mohan M, Winsauer P, Vande Stouwe C. Cannabinoid neuroimmune modulation of SIV disease. J Neuroimmune Pharmacol 2011;6(4):516-27.

Moller H-J. Non-neuroleptic approaches to treating negative symptoms in schizophrenia. Eur Arch Psychiatry Clin Neurosci 2004;254(2):108-16.

Morral AR, McCafrey DF, Paddock SM. Evidence does not favor marijuana gateway effects over a common-factor interpretation of drug use initiation: responses to Anthony, Kenkel & Mathios and Lynskey. Addiction 2002;97(12):1509-10.

Moulin D, Boulanger A, Clark AJ, Clarke H, Dao T, Finley GA, Furlan A, Gilron I, Gordon A, Morley-Forster PK, Sessle BJ, Squire P, Stinson J, Taenzer P, Velly A, Ware MA, Weinberg EL, Williamson OD, Canadian Pain Society. Pharmacological management of chronic neuropathic pain: revised consensus statement from the Canadian Pain Society. Pain Res Manag 2014;19(6):328-35.

Mujoomdar M, Spry C, Banks R. Cannabinoids for the treatment of post-traumatic stress disorder: a review of the clinical effectiveness and guidelines (Structured abstract). Health Technology Assessment Database 2016;(2016 Issue 4).

Murphy L, Ng KWK, Su VCH, Woodworth-Giroux S, Levy TS, Sproule BA, Furlan AD. Approach to the pharmacological management of chronic pain in patients with an alcohol use disorder. J Pain Res 2015;8:851-7.

Myles H, Myles N, Large M. Cannabis use in first episode psychosis: Meta-analysis of prevalence, and the time course of initiation and continued use. Aust N Z J Psychiatry 2016;50(3):208-19.

Nader DA, Sanchez ZM. Effects of regular cannabis use on neurocognition, brain structure, and function: a systematic review of findings in adults. Am J Drug Alcohol Abuse 2017;1-15.

Naftali T, Mechulam R, Lev LB, Konikoff FM. Cannabis for inflammatory bowel disease. Dig Dis 2014;32(4):468-74.

Nagarwala J, Dev S, Markin A. The Vomiting Patient: Small Bowel Obstruction, Cyclic Vomiting, and Gastroparesis. Emerg Med Clin North Am 2016;34(2):271-91.

Naguib M, Foss JF. Medical use of marijuana: Truth in evidence. Anesth Analg 2015;121(5):1124-7.

Nair KPS, Marsden J. The management of spasticity in adults. BMJ (Online) 2014;349:A2413.

National Academies of Sciences EaMU, Health and Medicine Division (US), Board on Population Health and Public Health Practice (US), Committee on the Health Effects of Marijuana: An Evidence Review and Research Agenda (US). The Health Effects of Cannabis and Cannabinoids: The Current State of Evidence and Recommendations for Research. Washington (DC): National Academies Press (US); 2017 Jan 2017.

Navari RM. Antiemetic control: toward a new standard of care for emetogenic chemotherapy. Expert Opin Pharmacother 2009;10(4):629-44.

Neumeister A. The endocannabinoid system provides an avenue for evidence-based treatment development for PTSD. Depression Anxiety 2013;30(2):93-6.

Ng L, Khan F, Young CA, Galea M. Symptomatic treatments for amyotrophic lateral sclerosis/motor neuron disease. Cochrane Database Syst Rev 2017;1:CD011776.

O'Connor AB, Schwid SR, Herrmann DN, Markman JD, Dworkin RH. Pain associated with multiple sclerosis: Systematic review and proposed classification. Pain 2008;137(1):96-111.

Ordean A, Wong S, Graves L. No. 349-Substance Use in Pregnancy. J Obstet Gynaecol Can 2017;39(10):922.

Orsolini L, Papanti D, Corkery J, De Luca MA, Cadoni C, Di Chiara G, Schifano F. Is there a Teratogenicity Risk Associated with Cannabis and Synthetic Cannabimimetics' ('Spice') Intake? CNS Neurol Disord Drug Targets 2017;16(5):585-91.

Ortolon K. Lighting up a controversy. Tex Med 2004;100(9):41-5.

Osborne AL, Solowij N, Weston-Green K. A systematic review of the effect of cannabidiol on cognitive function: Relevance to schizophrenia. Neurosci Biobehav Rev 2017;72:310-24.

Ostendorf AP, Ng Y-T. Treatment-resistant Lennox-Gastaut syndrome: Therapeutic trends, challenges and future directions. Neuropsychiatr Dis Treat 2017;13:1131-40.

Otero-Romero S, Sastre-Garriga J, Comi G, Hartung H-P, Soelberg SP, Thompson AJ, Vermersch P, Gold R, Montalban X. Pharmacological management of spasticity in multiple sclerosis: Systematic review and consensus paper. Mult Scler 2016;22(11):1386-96.

Oxentine H, Musec M, Johnson T. Medical marijuana: Can't we all just get a bong? Am J Addict 2017;26(3):277.

Papanti D, Schifano F, Botteon G, Bertossi F, Mannix J, Vidoni D, Impagnatiello M, Pascolo-Fabrici E, Bonavigo T. "Spiceophrenia": a systematic overview of "spice"-related psychopathological issues and a case report. HUM PSYCHOPHARMACOL 2013;28(4):379-89.

Papathanasopoulos P, Messinis L, Lyros E, Kastellakis A, Panagis G. Multiple sclerosis, cannabinoids, and cognition. J Neuropsychiatry Clin Neurosci 2008;20(1):36-51.

Park JY, Wu LT. Prevalence, reasons, perceived effects, and correlates of medical marijuana use: A review. Drug Alcohol Depend 2017;177:1-13.

Parsai S, Herman R, Johnson S. Systematic literature review of randomized controlled trials to evaluate the efficacy of medical marijuana for analgesia. Pharmacotherapy 2014;34(10):e287.

Pearl PL, Robbins EL, Bennett HD, Conry JA. Use of complementary and alternative therapies in epilepsy: Cause for concern. Arch Neurol 2005;62(9):1472-5.

Peng M, Khaiser M, Ahrari S, Pasetka M, DeAngelis C. Medical marijuana as a therapeutic option for cancer anorexia and cachexia: A scoping review of current evidence. J Pain Manage 2016;9(4):435-47.

Perras C. Sativex for the management of multiple sclerosis symptoms (Structured abstract). Health Technology Assessment Database 2016;(2016 Issue 4).

Pessoa BL, Escudeiro G, Nascimento OJM. Emerging Treatments for Neuropathic Pain. Curr Pain Headache Rep 2015;19(12):1-9.

Phillips JA, Holland MG, Baldwin DD, Meuleveld LG, Mueller KL, Perkison B, Upfal M, Dreger M. Marijuana in the workplace: guidance for occupational health professionals and employers: Joint Guidance Statement of the American Association of Occupational Health Nurses and the American College of Occupational and Environmental Medicine. J Occup Environ Med 2015;57(4):459-75.

Phillips R, Gopaul S, Gibson F, Houghton E, Craig J, Light K, Pizer B. Antiemetic medication for prevention and treatment of chemotherapy induced nausea and vomiting in childhood: A systematic reviewand meta-analysis. Pediatr Blood Cancer 2010;55(5):946.

Phillips TJC, Cherry CL, Cox S, Marshall S, Rice ASC. A metanalysis of randomised controlled clinical trials assessing the effectivness of pharmacological interventions for painful HIV-associated sensory neuropathy. Eur J Pain Suppl 2010;4(1):141.

Pidgeon C, Rickards H. The pathophysiology and pharmacological treatment of Huntington disease. BEHAV NEUROL 2013;26(4):245-53.

Pisanti S, Bifulco M. Endocannabinoid system modulation in cancer biology and therapy. Pharmacol Res 2009;60(2):107-16.

Pittler MH, Ernst E. Complementary therapies for neuropathic and neuralgic pain: systematic review. Clin J Pain 2008;24(8):731-3.

Posadzki P, Khalil MMK, AlBedah AMN, Zhabenko O, Car J. Complementary and alternative medicine for addiction: an overview of systematic reviews. Focus Altern Complement Ther 2016;21(2):69-81.

Potvin S, Joyal CC, Pelletier J, Stip E. Contradictory cognitive capacities among substance-abusing patients with schizophrenia: A meta-analysis. Schizophr Res 2008;100(1-3):242-51.

Prager C, Cross JH. Management of Dravet syndrome and emerging therapy options. Expert Opin Orphan Drugs 2017;5(3):219-27.

Prud'Homme M, Cata R, Jutras-Aswad D. Cannabidiol as an intervention for addictive behaviors: A systematic review of the evidence. Subst Abuse Res Treat 2015;9:33-8.

Quezada SM, Briscoe J, Cross RK. Complementary and alternative medicine. Inflammatory Bowel Dis 2016;22(6):1523-30.

Quintans JSS, Antoniolli AR, Almeida JRGS, Santana-Filho VJ, Quintans-Junior LJ. Natural products evaluated in neuropathic pain models - a systematic review. Basic Clin Pharmacol Toxicol 2014;114(6):442-50.

Rabin RA, Zakzanis KK, George TP. The effects of cannabis use on neurocognition in schizophrenia: a meta-analysis. Schizophr Res 2011;128(1-3):111-6.

Rai A, Meng H, Weinrib A, Englesakis M, Kumbhare D, Grosman-Rimon L, Katz J, Clarke H. A Review of Adjunctive CNS Medications Used for the Treatment of Post-Surgical Pain. CNS drugs 2017;31(7):605-15.

Reche M, Ivars D. Anorexia nervosa. treatment review. Eur Psychiatry 2015;30:1355.

Reddy DS, Golub VM. The Pharmacological Basis of Cannabis Therapy for Epilepsy. J Pharmacol Exp Ther 2016;357(1):45-55.

Rein JL, Wyatt CM. Marijuana and Cannabinoids in ESRD and Earlier Stages of CKD. Am J Kidney Dis 2017.

Reisner SH, Eisenberg NH, Stahl B, Hauser GJ. Maternal medications and breast-feeding. Dev Pharmacol Ther 1983;6(5):285-304.

Rey JM, Martin A, Krabman P. Is the party over? Cannabis and juvenile psychiatric disorder: the past 10 years. J Am Acad Child Adolesc Psychiatry 2004;43(10):1194-205.

Rigby E, Reid L, Schipperheijn JA, Weston L, Ikkos G. Clinical librarians: a journey through a clinical question. Health Info Libr J 2002;19(3):158-60.

Riggs PD. Stimulant medication for ADHD not associated with subsequent substance use disorders. Evid -Based Med 2014;19(2):78.

Rinaldi B, Vaisfeld A, Amarri S, Baldo C, Gobbi G, Magini P, Melli E, Neri G, Novara F, Pippucci T, Rizzi R, Soresina A, Zampini L, Zuffardi O, Crimi M. Guideline recommendations for diagnosis and clinical management of Ring14 syndrome - first report of an ad hoc task force. Orphanet J Rare Dis 2017;12(1):69.

Rinaldi L. Marijuana: a research overview. Alaska Med 1994;36(2):107-13.

Rocha FCM, Dos Santos Junior JG, Stefano SC, da Silveira DX. Systematic review of the literature on clinical and experimental trials on the antitumor effects of cannabinoids in gliomas. J Neurooncol 2014;116(1):11-24.

Rodrigo C, Rajapakse S. Cannabis and schizophrenia spectrum disorders: a review of clinical studies. Indian j psychol med 2009;31(2):62-70.

Rohleder C, Muller JK, Lange B, Leweke FM. Cannabidiol as a potential new type of an antipsychotic. A critical review of the evidence. Front Pharmacol 2016;7(NOV):422.

Roila F, Ciccarese G, Palladino MA, De A, V. Prevention of radiotherapy-induced emesis. Tumori 1998;84(2):274-8.

Roller L, Gauau J. Epilepsy. Aust J Pharm 2016;97(1155):62-72.

Rong C, Lee Y, Carmona NE, Cha DS, Ragguett R-M, Rosenblat JD, Mansur RB, Ho RC, McIntyre RS. Cannabidiol in medical marijuana: Research vistas and potential opportunities. Pharmacol Res 2017;121:213-8.

Rosati A, De MS, Guerrini R. Antiepileptic Drug Treatment in Children with Epilepsy. CNS drugs 2015;29(10):847-63.

Rosenberg EC, Tsien RW, Whalley BJ, Devinsky O. Cannabinoids and Epilepsy. Neurotherapeutics 2015;12(4):747-68.

Roser P, Haussleiter IS. Antipsychotic-like effects of cannabidiol and rimonabant: systematic review of animal and human studies. Curr Pharm Des 2012;18(32):5141-55.

Ross MW. Factors affecting information and education, and behaviour change. AIDS care 1991;3(4):419-21.

Roth MD, Baldwin GC, Tashkin DP. Effects of delta-9-tetrahydrocannabinol on human immune function and host defense. Chem Phys Lipids 2002;121(1-2):229-39.

Rothbart R, Stein DJ. Pharmacotherapy of trichotillomania (hair pulling disorder): an updated systematic review. Expert Opin Pharmacother 2014;15(18):2709-19.

Routsolias JC. In Response to: Cannabinoid Hyperemesis Syndrome: Diagnosis, Pathophysiology, and Treatment-a Systematic Review. J Med Toxicol 2017;13(2):197.

Rowbotham DJ. Pain management. Anaesthesia 2003;58(12):1196-9.

Rudnick A, Roe D. Toward a vision for the development, study, and use of electronic health technology for and by people with psychiatric and/or substance use disorders: A strengths, weaknesses, opportunities, and threats analysis and illustration. J Dual Diagn 2012;8(4):333-6.

Samavati R, Ducza E, Hajagos-Toth J, Gaspar R. Herbal laxatives and antiemetics in pregnancy. Reprod Toxicol 2017;72:153-8.

Sanadgol N, Zahedani SS, Sharifzadeh M, Khalseh R, Barbari GR, Abdollahi M. Recent Updates in Imperative Natural Compounds for Healthy Brain and Nerve Function: A Systematic Review of Implications for Multiple Sclerosis. Curr Drug Targets 2017;18(13):1499-517.

Sandor P. Pharmacological management of tics in patients with TS. J Psychosom Res 2003;55(1):41-8.

Sankhala KK, Karnad AB. New generation agents for the prevention of chemotherapy-induced nausea and vomiting. Eur J Clin Med Oncol 2011;3(3):10-5.

Santana TA, Trufelli DC, de Matos LL, Cruz FM, Del GA. Meta-analysis of adjunctive non-NK1 receptor antagonist medications for the control of acute and delayed chemotherapy-induced nausea and vomiting. Supportive Care Cancer 2015;23(1):213-22.

Sara GE. Drugs and psychosis ... and now for some good news. Aust New Zealand J Psychiatry 2014;48(5):484-5.

Sarris J, McIntyre E, Camfield DA. Plant-based medicines for anxiety disorders, part 1: A review of preclinical studies. CNS drugs 2013;27(3):207-19.

Saulino M, Averna JF. Evaluation and Management of SCI-Associated Pain. Curr Pain Headache Rep 2016;20(9):53.

Sawynok J. Topical Analgesics for Neuropathic Pain in the Elderly: Current and Future Prospects. Drugs Aging 2014;31(12):853-62.

Schatman ME, Darnall BD. Ethical pain care in a complex case. PAIN MED 2013;14(6):800-1.

Schatman ME, Darnall BD. Medical marijuana: a viable tool in the armamentaria of physicians treating chronic pain? A case study and commentary. PAIN MED 2013;14(6):799.

Schempf AH. Illicit drug use and neonatal outcomes: a critical review. Obstet Gynecol Surv 2007;62(11):749-57.

Schestatsky P, Vidor L, Winckler PB, de Araujo TG, Caumo W. Promising treatments for neuropathic pain. Arq Neuro-Psiquiatr 2014;72(11):881-8.

Schoeler T, Monk A, Sami M, Camuri G, Brown R, Bhattacharyya S. The effects of cannabis on relapse in psychosis. Schizophr Res 2014;153:S235.

Schoeler T, Kambeitz J, Behlke I, Murray R, Bhattacharyya S. The effects of cannabis on memory function in users with and without a psychotic disorder: findings from a combined meta-analysis. Psychol Med 2016;46(1):177-88.

Schroder S, Beckmann K, Franconi G, Meyer-Hamme G, Friedemann T, Greten HJ, Rostock M, Efferth T. Can medical herbs stimulate regeneration or neuroprotection and treat neuropathic pain in chemotherapy-induced peripheral neuropathy? Evid Based Complement Alternat Med 2013;2013:423713.

Selles RR, Murphy TK, Obregon D, Storch EA, Lewin AB. Treatment decisions for chronic tic disorders. Clin Pract 2013;10(6):765-80.

Serafini G, Pompili M, Innamorati M, Girardi P. The association between cannabis use and suicidal risk in psychosis. Eur Psychiatry 2012;27.

Shamloul R, Bella AJ. Impact of cannabis use on male sexual health. J Sex Med 2011;8(4):971-5.

Sharma R, Tobin P, Clarke SJ. Management of chemotherapy-induced nausea, vomiting, oral mucositis, and diarrhoea. Lancet Oncol 2005;6(2):93-102.

Shorter D, Hsieh J, Kosten TR. Pharmacologic management of comorbid post-traumatic stress disorder and addictions. Am J Addict 2015;24(8):705-12.

Simoff MJ, Lally B, Slade MG, Goldberg WG, Lee P, Michaud GC, Wahidi MM, Chawla M. Symptom management in patients with lung cancer: Diagnosis and management of lung cancer, 3rd ed: American college of chest physicians evidence-based clinical practice guidelines. Chest 2013;143(5 SUPPL):e455S-e497S.

Skalski L, Towe S, Sikkema K, Teitell S, Meade CS. Impact of marijuana use on memory in patients with HIV/AIDS: A systematic review. Drug Alcohol Depend 2015;146:e100.

Skalski LM, Towe SL, Sikkema KJ, Meade CS. The Impact of Marijuana Use on Memory in HIV-Infected Patients: A Comprehensive Review of the HIV and Marijuana Literatures. Curr Drug Abuse Rev 2017;9(2):126-41.

Smith KZ, Ravven S, Boyd JW. The impact of cannabis use on opioiddependence treatment: A systematic review. Drug Alcohol Depend 2015;146:e101-e102.

Smith SC, Wagner MS. Clinical endocannabinoid deficiency (CECD) revisited: Can this concept explain the therapeutic benefits of cannabis in migraine, fibromyalgia, irritable bowel syndrome and other treatment-resistant conditions? Neuroendocrinol Lett 2014;35(3):198-201.

Snedecor SJ, Sudharshan L, Cappelleri JC, Sadosky A, Desai P, Jalundhwala Y, Botteman M. Systematic review and meta-analysis of pharmacological therapies for pain associated with postherpetic neuralgia and less common neuropathic conditions. Int J Clin Pract 2014;68(7):900-18.

Soler R, Andersson KE, Chancellor MB, Chapple CR, de Groat WC, Drake MJ, Gratzke C, Lee R, Cruz F. Future direction in pharmacotherapy for non-neurogenic male lower urinary tract symptoms. Eur Urol 2013;64(4):610-21.

Sorensen CJ, DeSanto K, Borgelt L, Phillips KT, Monte AA. In Response to Letter to the Editor Regarding: Cannabinoid Hyperemesis Syndrome: Diagnosis, Pathophysiology, and Treatment-a Systematic Review. J Med Toxicol 2017;13(2):198.

Spithoff S, Emerson B, Spithoff A. Cannabis legalization: adhering to public health best practice. CMAJ 2015;187(16):1211-6.

Stajkovic S, Aitken EM, Holroyd-Leduc J. Unintentional weight loss in older adults. CMAJ 2011;183(4):443-9.

Stein M. Advancing pharmacological treatment of Posttraumatic Stress Disorder. Int J Neuropsychopharmacol 2010;13:40.

Steinborn JJ, Chinn AK, Carter GT. The latest buzz on medicinal marijuana: a legal and medical perspective. Am J Hosp Palliat Care 2001;18(5):295-8.

Strouse T. Cannabinoids in cancer treatment settings. J Community Supportive Oncol 2016;14(1):1-5.

Suarez-Pinilla P, Lopez-Gil J, Crespo-Facorro B. Immune system: a possible nexus between cannabinoids and psychosis. Brain Behav Immun 2014;40:269-82.

Sun-Edelstein C, Mauskop A. Alternative headache treatments: nutraceuticals, behavioral and physical treatments. Headache 2011;51(3):469-83.

Sun X, Xu CS, Chadha N, Chen A, Liu J. Marijuana for Glaucoma: A Recipe for Disaster or Treatment? Yale J Biol Med 2015;88(3):265-9.

Sznitman SR, Zolotov Y. Cannabis for therapeutic purposes and public health and safety: a systematic and critical review. Int J Drug Policy 2015;26(1):20-9.

Talamo A, Centorrino F, Tondo L, Dimitri A, Hennen J, Baldessarini RJ. Comorbid substance-use in schizophrenia: Relation to positive and negative symptoms. Schizophr Res 2006;86(1-3):251-5.

Tan C, Hatam N, Treasure T. Bullous disease of the lung and cannabis smoking: Insufficient evidence for a causative link. J R Soc Med 2006;99(2):77-80.

Teasell RW, Mehta S, Aubut J-A, Foulon B, Wolfe DL, Hsieh JTC, Townson AF, Short C. Pharmacological treatments of pain following SCI: A systematic review. J Spinal Cord Med 2009;32(4):479-80.

Tetrault JM, Crothers K. What are the pulmonary effects of smoking marijuana? J Respir Dis 2007;28(6):253-4.

Thaera GM, Wellik KE, Carter JL, Demaerschalk BM, Wingerchuk DM. Do cannabinoids reduce multiple sclerosis-related spasticity? Neurolog 2009;15(6):369-71.

Thomas D-A, West M, Legler A, Maslin B, Kim RK, Vadivelu N. Efficacy and safety of nonopioid analgesics in perioperative pain control. Curr Drug Saf 2016;11(3):196-205.

Togami JM. Voiding Dysfunction in Multiple Sclerosis: Disease Review and Management. Curr Bladder Dysfunct Rep 2013;8(4):304-11.

Tramer MR, Carroll D, Campbell FA, Reynolds DJ, Moore RA, McQuay HJ. Cannabinoids for control of chemotherapy induced nausea and vomiting: quantitative systematic review. BMJ 2001;323(7303):16-21.

Truini A, Galeotti F, Cruccu G. Treating pain in multiple sclerosis. Expert Opin Pharmacother 2011;12(15):2355-68.

Tsang CC, Giudice MG. Nabilone for the Management of Pain. Pharmacotherapy 2016;36(3):273-86.

Tubaro A, Puccini F, De NC, Digesu GA, Elneil S, Gobbi C, Khullar V. The treatment of lower urinary tract symptoms in patients with multiple sclerosis: A systematic review. Curr Urol Rep 2012;13(5):335-42.

Tuca A, Jimenez-Fonseca P, Gascon P. Clinical evaluation and optimal management of cancer cachexia. Crit Rev Oncol Hematol 2013;88(3):625-36.

Tucci SA. Phytochemicals in the control of human appetite and body weight. Pharmaceuticals 2010;3(3):748-63.

Turek PJ. Practical approaches to the diagnosis and management of male infertility. Nat Clin Pract Urol 2005;2(5):226-38.

Turgeman I, Bar-Sela G. Cannabis use in palliative oncology: A review of the evidence for popular indications. Isr Med Assoc J 2017;19(2):85-8.

Turna J, Patterson B, Van AM. Is cannabis treatment for anxiety, mood, and related disorders ready for prime time? Depression Anxiety 2017.

Turner D, Travis SPL, Griffiths AM, Ruemmele FM, Levine A, Benchimol EI, Dubinsky M, Alex G, Baldassano RN, Langer JC, Shamberger R, Hyams JS, Cucchiara S, Bousvaros A, Escher JC, Markowitz J, Wilson DC, Van AG, Russell RK. Consensus for managing acute severe ulcerative colitis in children: A systematic review and joint statement from ECCO, ESPGHAN, and the porto IBD working group of ESPGHAN. Am J Gastroenterol 2011;106(4):574-88.

Tyler LS, Lipman AG. Anorexia and cachexia in palliative care patients. J Pharm Care Pain Symptom Control 1999;7(4):11-22.

van den Brink W, Goppel M, van Ree JM. Management of opioid dependence. Curr Opin Psychiatry 2003;16(3):297-304.

van der Meer FJ, Velthorst E, Meijer CJ, Machielsen MWJ, de Haan L. Cannabis use in patients at clinical high risk of psychosis: impact on prodromal symptoms and transition to psychosis. Curr Pharm Des 2012;18(32):5036-44.

van Os J, Krabbendam L, Myin-Germeys I, Delespaul P. The schizophrenia envirome. Curr Opin Psychiatry 2005;18(2):141-5.

Vasconcelos SC, Silva AO, Moreira MASP, Correia AdSB, Guerra ALAG, Santos ARD, Frazao IdS. Bioethical analysis to the therapeutic use of Cannabis: Integrative review. Nurs Ethics 2017;969733017703699.

Viteri OA, Soto EE, Bahado-Singh RO, Christensen CW, Chauhan SP, Sibai BM. Fetal anomalies and long-term effects associated with substance abuse in pregnancy: A literature review. Am J Perinatol 2015;32(5 Supplement 11):405-16.

Volk DW, Lewis DA. GABA targets for the treatment of cognitive dysfunction in schizophrenia. Curr Neuropharmacol 2005;3(1):45-62.

Volkow ND, Compton WM, Wargo EM. The risks of marijuana use during pregnancy. Obstet Gynecol Surv 2017;72(5):257-8.

von Haehling S, Lainscak M, Springer J, Anker SD. Cardiac cachexia: a systematic overview. Pharmacol Ther 2009;121(3):227-52.

Vyas MB, LeBaron VT, Gilson AM. The use of cannabis in response to the opioid crisis: A review of the literature. Nurs Outlook 2017.

Wade DT, Collin C, Stott C, Duncombe P. Meta-analysis of the efficacy and safety of Sativex (nabiximols), on spasticity in people with multiple sclerosis. Mult Scler 2010;16(6):707-14.

Waldon K, Hill J, Termine C, Balottin U, Cavanna AE. Trials of pharmacological interventions for Tourette Syndrome: A systematic review. BEHAV NEUROL 2013;26(4):265-73.

Wallace JM. Update on pharmacotherapy guidelines for treatment of neuropathic pain. Curr Pain Headache Rep 2007;11(3):208-14.

Wallace MS, Ware MA. Medicinal Marijuana: Here to Stay and Time to Take Responsibility. Clin J Pain 2015;31(11):931-2.

Ware MA, Dubin R, Lynch M. Cannabis for pain: Evidence, education and evolution. Pain Res Manage 2015;20(3):e33.

Ware MA, St Arnaud-Trempe E. The abuse potential of the synthetic cannabinoid nabilone. Addiction 2010;105(3):494-503.

Wasserman P, Segal-Maurer S, Rubin DS. The treatment of human immunodeficiency virus-associated wasting syndrome with recombinant human growth hormone, then and now: A pivotal paper review. Nutr Clin Prac 2004;19(4):388-94.

Watson CPN, Gilron I, Sawynok J, Lynch ME. Nontricyclic antidepressant analgesics and pain: Are serotonin norepinephrine reuptake inhibitors (SNRIs) any better? Pain 2011;152(10):2206-10.

Watts DC, Oakley ER, Malashock HR, Moore EC, LoVecchio F. Articles You Might Have Missed. J Med Toxicol 2017;13(1):95-8.

WCB Evidence Based Practice Group. Efficacy of marijuana in treating chronic non cancer pain: a short review (Structured abstract). Health Technology Assessment Database 2016;(2016 Issue 4).

Weinshenker BG. Cannabinoids did not reduce muscle spasticity in stable multiple sclerosis. ACP J Club 2004;140(3):69.

Weiss PA. Does smoking marijuana contribute to the risk of developing lung cancer? Clin J Oncol Nurs 2008;12(3):517-9.

Wilkinson JT, Fraunfelder FW. Use of herbal medicines and nutritional supplements in ocular disorders: An evidence-based review. Drugs 2011;71(18):2421-34.

Wilkinson ST. Medical and recreational marijuana: commentary and review of the literature. Mo Med 2013;110(6):524-8.

Wilkinson ST, Radhakrishnan R, D'Souza DC. A Systematic Review of the Evidence for Medical Marijuana in Psychiatric Indications. J Clin Psychiatry 2016;77(8):1050-64.

Williams IL. The intersection of structurally traumatized communities and substance use treatment: Dominant discourses and hidden themes. J Ethn Subst Abuse 2016;15(2):95-126.

Williams JHG, Ross L. Consequences of prenatal toxin exposure for mental health in children and adolescents: A systematic review. European Child & Adolescent Psychiatry 2007;16(4):243-53.

Windisch PA, Papatheofanis FJ, Matuszewski KA. Recombinant human growth hormone for AIDS-associated wasting. Ann Pharmacother 1998;32(4):437-45.

Winstock AR, Ford C, Witton J. Assessment and management of cannabis use disorders in primary care. BMJ (Online) 2010;340(7750):800-4.

Woller A, Berthele A, Leucht S, Tolle TR. Antidepressants for central pain. Cochrane Database Syst Rev 2007;(3):CD006631.

Wong SS, Wilens TE. Medical Cannabinoids in Children and Adolescents: A Systematic Review. Pediatrics 2017;140(5).

Wood S. Evidence for using cannabis and cannabinoids to manage pain. Nurs Times 2004;100(49):38-40.

Wright S. Summary of evidence-based guideline: Complementary and alternative medicine in multiple sclerosis: Report of the guideline development subcommittee of the American Academy of Neurology. Neurology 2014;83(16):1484-5.

Wu SW, Harris E, Gilbert DL. Tic suppression: The medical model. J Child Adolesc Psychopharmacol 2010;20(4):263-76.

Yadav V, Narayanaswami P. Complementary and alternative medical therapies in multiple sclerosis--the American Academy of Neurology guidelines: a commentary. Clin Ther 2014;36(12):1972-8.

Yap M, Easterbrook L, Connors J, Koopmans L. Use of cannabis in severe childhood epilepsy and child protection considerations. J Paediatr Child Health 2015;51(5):491-6.

Yarnell S. The Use of Medicinal Marijuana for Posttraumatic Stress Disorder: A Review of the Current Literature. Prim Care Companion CNS Disord 2015;17(3).

Yeh S-S, Lovitt S, Schuster MW. Pharmacological Treatment of Geriatric Cachexia: Evidence and Safety in Perspective. J Am Med Dir Assoc 2007;8(6):363-77.

Yorio T, Dibas A. New therapies for glaucoma: Are they all up to the task? Expert Opin Ther Pat 2004;14(12):1743-62.

Yucel M, Bora E, Lubman DI, Solowij N, Brewer WJ, Cotton S, Conus P, Fornito A, McGorry PD, Pantelis C. The impact of cannabis use on cognitive functioning in schizophrenia: A meta-analysis of existing findings and new data. Eur Neuropsychopharmacol 2010;20:S432.

Yurcheshen M, Seehuus M, Pigeon W. Updates on nutraceutical sleep therapeutics and investigational research. Evid -Based Complement Altern Med 2015;2015:105256.

Zer-Aviv TM, Segev A, Akirav I. Cannabinoids and post-traumatic stress disorder: Clinical and preclinical evidence for treatment and prevention. Behav Pharmacol 2016;27(7):561-9.

Zettl UK, Rommer P, Hipp PETR, Patejdl R. Evidence for the efficacy and effectiveness of THC-CBD oromucosal spray in symptom management of patients with spasticity due to multiple sclerosis. Ther Adv Neurol Disord 2016;9(1):9-30.

Zhornitsky S, Potvin S. Cannabidiol in humans-the quest for therapeutic targets. Pharmaceuticals (Basel) 2012;5(5):529-52.

Zielinski L, Bhatt M, Eisen RB, Perera S, Bhatnagar N, MacKillop J, Steiner M, McDermid Vaz S, Thabane L, Samaan Z. Association between cannabis use and treatment outcomes in patients receiving methadone maintenance treatment: a systematic review protocol. Syst rev 2016;5(1):139.

Zlebnik NE, Cheer JF. Beyond the CB1 Receptor: Is Cannabidiol the Answer for Disorders of Motivation? Annu Rev Neurosci 2016;39:1-17.

Zuardi AW, Crippa JA, Hallak JEC, Bhattacharyya S, Atakan Z, Martin-Santos R, McGuire PK, Guimaraes FS. A critical review of the antipsychotic effects of cannabidiol: 30 years of a translational investigation. Curr Pharm Des 2012;18(32):5131-40.

**LANGUAGE**

Drug and Non-Drug Treatment Strategies to Assist Smoking Cessation. Therapie 2003;58(6):479-97. ***Published in French.***

[Testing the administration of cannabinoids in neurological diseases]. Ideggyogy Sz 2005;58(5-6):175-6. ***Published in Hungarian.***

Al-Khalil O. [Cannabinoids in medical procedures - Review and Meta-analysis]. Praxis (Bern 1994) 2015;104(20):1103-4. ***Published in German.***

Allende-Salazar RF, Rada G. Are cannabinoids an effective treatment for chronic non-cancer pain? Medwave 2017;17(Suppl2):e6972 ***Published in Spanish***

Ballester Verneda MR. Therapeutic use of cannabis for the prevention of antineoplastic chemotherapy induced nausea and vomiting (II). Circ Farm 2002;60(2):5-13. ***Published in Spanish***

Bazinski H, Jensen HB, Stenager E. [There is evidence for the use of cannabinoids for symptomatic treatment of multiple sclerosis]. Ugeskr Laeger 2015;177(20):956-60. ***Published in Danish.***

Beaulieu P, Rice ASC. [The pharmacology of cannabinoid derivatives: are there applications to treatment of pain?]. Ann Fr Anesth Reanim 2002;21(6):493-508. ***Published in French.***

Benze G, Geyer A, Alt-Epping B, Nauck F. [Treatment of nausea and vomiting with 5HT3 receptor antagonists, steroids, antihistamines, anticholinergics, somatostatinantagonists, benzodiazepines and cannabinoids in palliative care patients : a systematic review]. Schmerz 2012;26(5):481-99. ***Published in German.***

Bifulco M, Di Marzo V. [The endocannabinoid system as a target for the development of new drugs for cancer therapy]. Recenti Prog Med 2003;94(5):194-8. ***Published in Italian.***

Bjorkhem-Bergman L, Wallin C. [Methylphenidate and cannabinoids may have a place in palliative care. Literature review and clinical experience provides evidence]. Lakartidningen 2013;110(32-33):1409-11. ***Published in Swedish***

Bogaczewicz A, Sobow T, Bogaczewicz J, Sysa-Jedrzejowska A, Wozniacka A. Meta-analysis of the usage of drugs affecting the central nervous system for cholestatic pruritus. Dematol Kliniczna 2012;14(1):5-12. ***Published in German.***

Bravo-Soto GA, Juri C. Are cannabinoids effective for Parkinsons disease? Medwave 2017;17(Suppl2):e6974 ***Published in Spanish***

Centre for Reviews and Dissemination. Treatment of nausea and vomiting with 5HT3 receptor antagonists, steroids, antihistamines, anticholinergics, somatostatin antagonists, benzodiazepines and cannabinoids in palliative care patients: a systematic review (Provisional abstract). Database of Abstracts of Reviews of Effects 2015;(2). ***Published in German.***

Duran-Ferreras E, Chacon JR. Treatment of psychosis in Parkinson. Rev Neurol 2009;48(12):645-53. ***Published in Spanish***

Guinguis R, Ruiz MI, Rada G. Is cannabidiol an effective treatment for schizophrenia? Medwave 2017;17(7):e7010 ***Published in Spanish***

Henze T. Symptomatic treatment for multiple sclerosis. Nervenarzt 2004;75(SUPPL. 1):S2-S39 ***Published in German.***

Karila L, Cazas O, Danel T, Reynaud M. [Short- and long-term consequences of prenatal exposure to cannabis]. J Gynecol Obstet Biol Reprod (Paris) 2006;35(1):62-70. ***Published in French.***

Kreutzkamp B. Cannabinoids in medicine: Level of evidence rather poor until now - A meta-analysis. Krankenhauspharmazie 2015;36(10):516-7. ***Published in German.***

Langhorst J. Complementary and alternative medicine treatments in inflammatory bowel diseases. J Gastroenterol Hepatol Erkr 2016;14(1):9-16. ***Language unclear***

Lobos Urbina D, Pena Duran J. Are cannabinoids effective for treatment of pain in patients with active cancer? Medwave 2016;16 Suppl 3:e6539 ***Published in Spanish***

Marxer N. Cannabinoids: Serious side-effects limit the use. Pharm Ztg 2001;146(34):36-7. ***Language unclear***

Meza R, Pena J, Garcia K, Corsi O, Rada G. Are cannabinoids effective in multiple sclerosis? Medwave 2017;17(Suppl1):e6865 ***Published in Spanish***

Mucke M, Carter C, Cuhls H, Prus M, Radbruch L, Hauser W. [Cannabinoids in palliative care: Systematic review and meta-analysis of efficacy, tolerability and safety]. Schmerz 2016;30(1):25-36. ***Published in German.***

Neuhaus O, Kieseier BC, Klimke A, Gaebel W, Hohlfeld R, Hartung HP. [Cannabinoids in multiple sclerosis. Opportunity or hazard?]. Nervenarzt 2004;75(10):1022-6. ***Published in German.***

Pena J, Rada G. Are cannabinoids effective for epilepsy? Medwave 2017;17(Suppl1):e6821 ***Published in Spanish***

Petzke F, Enax-Krumova EK, Hauser W. [Efficacy, tolerability and safety of cannabinoids for chronic neuropathic pain: A systematic review of randomized controlled studies]. Schmerz 2016;30(1):62-88. ***Published in German.***

Pollmann W, Feneberg W, Steinbrecher A, Haupts MR, Henze T. [Therapy of pain syndromes in multiple sclerosis -- an overview with evidence-based recommendations]. Fortschr Neurol Psychiatr 2005;73(5):268-85. ***Published in German.***

Radbruch L, Nauck F. Review of cannabinoids in the treatment of nausea and vomiting. Schmerz 2004;18(4):306-10. ***Published in German.***

Ribeiro M, Marques ACPR, Laranjeira R, Alves HNP, Araujo MRd, Baltieri DA, Bernardo WM, Castro LAGP, Karniol IG, Kerr-Correa F, Nicastri S, Nobre MRC, Oliveira RAd, Romano M, Seibel SD, Silva CJd. [Marijuana Abuse]. Rev Assoc Med Bras 2005;51(5):247-9. ***Published in Portuguese***

Rivera-Olmos VM, Parra-Bernal MC. [Cannabis: Effects in the Central Nervous System. Therapeutic, societal and legal consequences]. REV MED INST MEX SEGURO SOC 2016;54(5):626-34. ***Published in Spanish***

Sales J, Arnaiz JA. Cannabinoids, a myth or a scientific reality? Med Paliativa 2002;9(3):129-33. ***Published in Portuguese***

Soyka M, Preuss U, Hoch E. [Cannabis-induced disorders]. Nervenarzt 2017;88(3):311-25. ***Published in German***

Stepien A, Domzal TM. [New guidelines for the treatment of neuropathic pain]. Neurol Neurochir Pol 2010;44(5):437-42. ***Published in Polish***

Villegas R. Cannabis therapeutic utility (Structured abstract). Health Technology Assessment Database 2016;(2016 Issue 4). ***Language unclear***

Volz MS, Siegmund B, Hauser W. [Efficacy, tolerability, and safety of cannabinoids in gastroenterology: A systematic review]. Schmerz 2016;30(1):37-46. ***Published in German***

Wasielewski S. German Pain Management Congress: Cannabinoids in medicine. Dtsch Apoth Ztg 2003;143(45):36-8. ***Published in German***

**MARIJUANA USE/ABUSE**

Beard S, Hunn A, Wight J. Treatments for spasticity and pain in multiple sclerosis: a systematic review. Health Technol Assess 2003;7(40):iii-111

Branas P, Jordan R, Fry-Smith A, Burls A, Hyde C. Treatments for fatigue in multiple sclerosis: a rapid and systematic review. Health Technol Assess 2000;4(27):1-61.

Carney R, Cotter J, Firth J, Bradshaw T, Yung AR. Cannabis use and symptom severity in individuals at ultra high risk for psychosis: a meta-analysis. Acta Psychiatr Scand 2017;136(1):5-15.

Cashman CM, Beirne P, Greiner B, Verbeek J. Alcohol and drug screening of occupational drivers for preventing injury. Cochrane Database Syst Rev 2007;(2):CD006566.

Centre for Reviews and Dissemination. (Provisional abstract). Database of Abstracts of Reviews of Effects 2015;(2).

Centre for Reviews and Dissemination. A systematic review of school-based marijuana and alcohol prevention programs targeting adolescents aged 10-15 (Structured abstract). Database of Abstracts of Reviews of Effects 2015;(2).

Centre for Reviews and Dissemination. Cannabis for therapeutic purposes and public health and safety: a systematic and critical review (Provisional abstract). Database of Abstracts of Reviews of Effects 2015;(2).

Centre for Reviews and Dissemination. Systematic review and meta-analysis on the adverse events of rimonabant treatment: considerations for its potential use in hepatology (Structured abstract). Database of Abstracts of Reviews of Effects 2015;(2).

Conner SN, Bedell V, Lipsey K, Macones GA, Cahill AG, Tuuli MG. Maternal Marijuana Use and Adverse Neonatal Outcomes: A Systematic Review and Meta-analysis. Obstet Gynecol 2016;128(4):713-23.

Farley AC, Hajek P, Lycett D, Aveyard P. Interventions for preventing weight gain after smoking cessation. Cochrane Database Syst Rev 2012;1:CD006219

Gibbs M, Winsper C, Marwaha S, Gilbert E, Broome M, Singh SP. Cannabis use and mania symptoms: a systematic review and meta-analysis. J Affect Disord 2015;171:39-47.

Goldenberg M, IsHak WW, Danovitch I. Quality of life and recreational cannabis use. Am J Addict 2017;26(1):8-25.

Gunn JKL, Rosales CB, Center KE, Nunez A, Gibson SJ, Christ C, Ehiri JE. Prenatal exposure to cannabis and maternal and child health outcomes: a systematic review and meta-analysis. BMJ open 2016;6(4):e009986

Gupta P, Mullin K, Nielssen O, Harris A, Large M. Do former substance users with psychosis differ in their symptoms or function from non-substance users? A systematic meta-analysis. Aust N Z J Psychiatry 2013;47(6):524-37.

Harrison AM, Heritier F, Childs BG, Bostwick JM, Dziadzko MA. Systematic Review of the Use of Phytochemicals for Management of Pain in Cancer Therapy. BioMed Res Int 2015;2015:506327.

Hecht I, Achiron A, Man V, Burgansky-Eliash Z. Modifiable factors in the management of glaucoma: a systematic review of current evidence. Graefe's Arch Clin Exp Ophthalmol 2017;255(4):789-96.

Lu J, Gary KW, Neimeier JP, Ward J, Lapane KL. Randomized controlled trials in adult traumatic brain injury. Brain Inj 2012;26(13-14):1523-48.

Marshall M, Rathbone J. Early intervention for psychosis. Cochrane Database Syst Rev 2011;(6):CD004718

McCrea JT, Langhorne P, Pandyan AD, Pollock A, Van WF. Systemically-acting pharmacological interventions for spasticity after stroke. Cochrane Database Syst Rev 2008;(1):CD006874.

Ruisch IH, Dietrich A, Glennon JC, Buitelaar JK, Hoekstra PJ. Maternal substance use during pregnancy and offspring conduct problems: A meta-analysis. Neurosci Biobehav Rev 2017;

Ruiz-Veguilla M, Callado LF, Ferrin M. Neurological soft signs in patients with psychosis and cannabis abuse: a systematic review and meta-analysis of paradox. Curr Pharm Des 2012;18(32):5156-64.

Schoeler T, Monk A, Sami MB, Klamerus E, Foglia E, Brown R, Camuri G, Altamura AC, Murray R, Bhattacharyya S. Continued versus discontinued cannabis use in patients with psychosis: a systematic review and meta-analysis. Lancet Psychiatry 2016;3(3):215-25.

Watson CPN, Gilron I, Sawynok J. A qualitative systematic review of head-to-head randomized controlled trials of oral analgesics in neuropathic pain. Pain Res Manage 2010;15(3):147-57.

Wilson RP, Bhattacharyya S. Antipsychotic efficacy in psychosis with co-morbid cannabis misuse: A systematic review. J Psychopharmacol 2016;30(2):99-111.

Zammit S, Moore THM, Lingford-Hughes A, Barnes TRE, Jones PB, Burke M, Lewis G. Effects of cannabis use on outcomes of psychotic disorders: systematic review. Br J Psychiatry 2008;193(5):357-63.

**FULL TEXT NOT AVAILABLE**

12th European Congress of Endocrinology 2010. Endocrine Abstracts 2010;22.

6th Annual Meeting Frontiers in Neurology. Ir J Med Sci 2017;186(7 Supplement 1).

BFS Annual Meeting 2014. Hum Fertil 2014;17(2).

Cannabis derivatives and pain. A small role for delta9-tetrahydrocannabinol (THC) in some forms of multiple sclerosis. Prescrire Int 2009;18(103):226

Archie S, Gyomorey K. First episode psychosis, substance abuse and prognosis: A systematic review. Curr Psychiatry Rev 2009;5(3):153-63.

Burggren AC. The effects of cannabis use among older adults. In: Adler, editors. Complementary and integrative therapies for mental health and aging, New York, NY, US: Oxford University Press; 2016. p. 109-20.

Castle D, Murray R. Marijuana and madness: Psychiatry and neurobiology. New York, NY, US: Cambridge University Press; 2004.

Centre for Reviews and Dissemination. Antipsychotic drug treatment of schizophrenic patients with substance abuse disorders (Provisional abstract). Database of Abstracts of Reviews of Effects 2015;(2).

Centre for Reviews and Dissemination. Are cannabinoids more effective than placebo in decreasing MS-related bladder dysfunction? (Provisional abstract). Database of Abstracts of Reviews of Effects 2015;(2).

Fride E, Russo E. Neuropsychiatry: Schizophrenia, Depression, and Anxiety. In: Andreasson, editors. Endocannabinoids: The brain and body's marijuana and beyond, Boca Raton, FL, US: CRC Press; 2006. p. 371-82.

Karila L, Benyamina A, Blecha L, Cottencin O, Billieux J. The Synthetic Cannabinoids Phenomenon. Curr Pharm Des 2016;22(42):6420-5.

Mahvan TD, Hilaire ML, Mann A, Brown A, Linn B, Gardner T, Lai B. Marijuana Use in the Elderly: Implications and Considerations. Consult Pharm 2017;32(6):341-51.

Nahas GG, Sutin KM, Harvey D, et al. Marihuana and medicine. Totowa, NJ, US: Humana Press; 1999.

Palleria C, Cozza G, Khengar R, Libri V, De Sarro G. Safety profile of the newest antiepileptic drugs: a curated literature review. Curr Pharm Des 2017;

Ritsner MS. Is a neuroprotective therapy suitable for schizophrenia patients? In: Addington, editors. Brain protection in schizophrenia, mood and cognitive disorders, New York, NY, US: Springer Science + Business Media; 2010. p. 343-95.

Serafini G, Pompili M, Innamorati M, Rihmer Z, Sher L, Girardi P. Can cannabis increase the suicide risk in psychosis? A critical review. Curr Pharm Des 2012;18(32):5165-87.

Taylor HG. Analysis of the medical use of marijuana and its societal implications. J Am Pharm Assoc (Wash) 1998;38(2):220-7.

Walsh BT. Pharmacological treatment. In: Psychobiology and treatment of anorexia nervosa and bulimia nervosa, Arlington, VA, US: American Psychiatric Association; 1992. p. 329-40.

**DUPLICATES**

CADTH. Nabilone for Non-chemotherapy Associated Nausea and Weight Loss due to Medical Conditions: A Review of the Clinical Effectiveness and Guidelines. Rapid Response Report: Summary with Critical Appraisal. Ottawa (ON): Canadian Agency for Drugs and Technologies in Health; 2014 Sep 12.

Centre for Reviews and Dissemination. A systematic review of pharmacologic treatments of pain after spinal cord injury (Structured abstract). Database of Abstracts of Reviews of Effects 2015;(2).

Centre for Reviews and Dissemination. Adverse effects of medical cannabinoids: a systematic review (Structured abstract). Database of Abstracts of Reviews of Effects 2015;(2).

Centre for Reviews and Dissemination. Are cannabinoids an effective and safe treatment option in the management of pain: a qualitative systematic review (Structured abstract). Database of Abstracts of Reviews of Effects 2015;(2).

Centre for Reviews and Dissemination. Cannabinoids for treatment of chronic non-cancer pain: a systematic review of randomized trials (Structured abstract). Database of Abstracts of Reviews of Effects 2015;(2).

Markovic D, Bosnjak D, Brkovic T, Jeric M, Rubic Z, Vuica VA, Puljak L. Cannabinoids for the treatment of dementia. Cochrane Database Syst Rev 2017;2017(10):CD012820.

**ANIMAL STUDY**

England TJ, Hind WH, Rasid NA, O'Sullivan SE. Cannabinoids in experimental stroke: A systematic review and meta-analysis. J Cereb Blood Flow Metab 2015;35(3):348-58.

**NO RCT DATA**

Harrison F, Aerts L, Brodaty H. Apathy in Dementia: Systematic Review of Recent Evidence on Pharmacological Treatments. Curr Psychiatry Rep 2016;18(11):103.

Hesketh PJ, Kris MG, Basch E, Bohlke K, Barbour SY, Clark-Snow RA, Danso MA, Dennis K, Dupuis LL, Dusetzina SB, Eng C, Feyer PC, Jordan K, Noonan K, Sparacio D, Somerfield MR, Lyman GH. Antiemetics: American Society of Clinical Oncology clinical practice guideline update. J Clin Oncol 2017;35(28):3240-61.

Zammit S, Moore THM, Lingford-Hughes A, Barnes TRE, Jones PB, Burke M, Lewis G. Effects of cannabis use on outcomes of psychotic disorders: systematic review. Br J Psychiatry 2008;193(5):357-63.
