# Supplementary material for: Benefits and harms of medical cannabis: a scoping review of systematic reviews
Source: Syst Rev. 2019 Dec 10;8:320. doi: 10.1186/s13643-019-1243-x (PMC6905063; doi:10.1186/s13643-019-1243-x)
Supplement: Supplementary file 8 — Additional file 8. AMSTAR Scores by Review. [file 13643_2019_1243_MOESM8_ESM.docx]

**Appendix 6: AMSTAR 2 Scores, by Review**

| **Author, Year** | **AMSTAR-2 Criterion** | | | | | | | | | | | | | | | | | |
| --- | --- | --- | --- | --- | --- | --- | --- | --- | --- | --- | --- | --- | --- | --- | --- | --- | --- | --- |
|  | **Q1** | **Q2** | **Q3** | ***Q4** | **Q5** | **Q6** | **Q7** | **Q8** | ***Q9A** | **Q10** | ***Q11A** | **Q12** | ***Q13** | **Q14** | **Q15** | **Q16** | **Overall score** | |
| Herzog, 2017^4^ | N | PY | N | Y | Y | N | Y | Y | N | Y | No MA | No MA | Y | N | N | Y | **L** |  |
| Claflin, 2018^23^ | N | N | N | N | N | N | N | Y | PY | N | No MA | No MA | Y | N | N | N | **L** |  |
| Behm, 2017^24^ | N | N | Y | PY | Y | Y | N | Y | Y | N | No MA for cannabis | No MA for cannabis | N | N | N | N | **L** |  |
| Kim, 2017^97^ | N | N | N | PY | N | N | N | Y | Y | N | No MA | No MA | Y | N | N | Y | **M** |  |
| Aviram, 2017^26^ | Y | N | N | PY | N | N | Y | Y | PY | N | Y | Y | PY | Y | N | Y | **M** |  |
| Da Rovare, 2017^27^ | Y | N | N | Y | Y | Y | N | Y | Y | N | Y | Y | Y | Y | Y | Y | **M** |  |
| Norton, 2017^28^ | Y | PY | Y | N | N | N | Y | Y | includes only NRSI | N | No MA | No MA | N | N | N | N | **CL** |  |
| Walsh, 2017^22^ | N | N | N | N | N | N | N | Y | N | N | No MA | No MA | N | N | N | Y | **CL** |  |
| Snedecor, 2013^29^ | N | N | N | N | Y | Y | N | Y | Y | N | Y | Y | N | Y | N | Y | **CL** |  |
| Goldenberg, 2017^30^ | N | N | N | PY | Y | N | N | Y | N | N | N | Y | Y | N | N | Y | **L** |  |
| Meng, 2017^31^ | Y | Y | N | Y | Y | N | N | Y | Y | N | Y | Y | Y | Y | Y | Y | **M** |  |
| Nugent, 2017^32^ | N | Y | Y | Y | Y | Y | N | Y | Y | N | Y | Y | Y | Y | N | Y | **M** |  |
| O’Neil, 2017^33^ | Y | Y | N | Y | N | N | N | Y | includes only NRSI | N | No MA | No MA | Y | N | N | Y | **M** |  |
| Youssef, 2017^34^ | N | Y | N | PY | Y | Y | N | Y | Y | Y | N | Y | Y | N | N | Y | **L** |  |
| Mehta, 2016^35^ | N | N | N | PY | Y | N | N | PY | Y | N | No MA | No MA | N | N | N | N | **L** |  |
| van den Beuken-van Everdingen, 2017^84^ | Y | N | N | N | Y | N | N | Y | Y | N | No MA | No MA | N | N | N | N | **CL** |  |
| Fitzcharles, 2016^36^ | Y | N | N | Y | Y | Y | N | Y | Y | Y | No MA | No MA | Y | N | N | Y | **M** |  |
| Tateo, 2017^37^ | N | N | N | PY | N | N | N | Y | Y | N | No Ma | No Ma | Y | N | N | N | **M** |  |
| Stevens, 2017^38^ | Y | Y | N | PY | Y | Y | N | PY | Y | N | No MA | No MA | Y | N | N | Y | **M** |  |
| Walitt, 2016^39^ | Y | PY | N | Y | Y | Y | Y | Y | Y | Y | No MA | No MA | Y | N | N | Y | **M** |  |
| Smith, 2015^40^ | Y | PY | N | Y | Y | Y | Y | Y | Y | N | Y | Y | Y | Y | N | Y | **M** |  |
| Deshpande, 2015^41^ | N | N | N | N | Y | Y | N | Y | Y | N | no MA | no MA | Y | Y | N | N | **L** |  |
| Andreae, 2015^42^ | Y | Y | Y | PY | Y | Y | Y | Y | Y | Y | Y | Y | Y | N | N | Y | **M** |  |
| Whiting, 2015^43^ | N | Y | Y | PY | Y | Y | Y | Y | Y | N | Y | Y | Y | Y | N | Y | **M** |  |
| Langhorst, 2015^44^ | N | PY | N | PY | Y | Y | PY | Y | Y | N | No MA | No MA | Y | N | N | Y | **M** |  |
| McLoughlin, 2014^45^ | Y | PY | Y | Y | Y | Y | Y | Y | Y | Y | No MA for this question | No MA | Y | Y | Y | Y | **H** |  |
| Koppel, 2014^85^ | N | N | N | PY | N | N | N | Y | PY | N | N | No MA | Y | N | N | Y | **L** |  |
| Gates, 2014^46^ | N | N | N | PY | Y | N | N | N | N | N | No MA | No MA | Y | N | N | N | **L** |  |
| Yadav, 2014^92^ | N | N | N | PY | Y | N | N | Y | Y | N | No MA | No MA | Y | N | N | Y | **M** |  |
| van den Elsen, 2014^47^ | N | PY | N | PY | Y | N | N | Y | Y | N | No MA | No MA | Y | N | N | N | **M** |  |
| Lutge, 2013^48^ | Y | N | N | PY | Y | Y | Y | Y | Y | N | No MA | No MA | Y | Y | N | N | **M** |  |
| Fitzcharles, 2016^49^ | Y | N | N | N | N | Y | N | PY | PY | N | No MA | No MA | Y | N | N | N | **M** |  |
| Baldinger, 2012^50^ | N | Y | N | N | Y | Y | Y | Y | Y | N | No MA | No MA | Y | Y | N | Y | **L** |  |
| Richards, 2012^51^ | Y | Y | N | Y | Y | Y | Y | Y | Y | N | No MA | No MA | Y | Y | N | Y | **M** |  |
| Lynch, 2011^52^ | Y | N | N | PY | N | N | N | PY | PY | N | No MA | No MA | N | N | N | N | **L** |  |
| Phillips, 2010^53^ | N | N | N | PY | N | N | Y | Y | N | N | N | Y | Y | N | N | Y | **CL** |  |
| Phillips, 2010^54^ | Y | N | N | PY | Y | Y | Y | Y | PY | Y | No MA | No MA | Y | Y | Y | Y | **M** |  |
| Meyer, 2010^55^ | N | N | N | PY | N | N | N | N | PY | N | No MA | No MA | Y | N | N | N | **M** |  |
| Lakhan, 2009^56^ | Y | N | N | PY | N | Y | N | PY | PY | N | No MA | No MA | Y | Y | N | N | **M** |  |
| Curtis, 2009^57^ | Y | Y | N | PY | N | N | Y | Y | Y | N | No MA | No MA | Y | N | No MA | Y | **M** |  |
| Martin-Sanchez, 2009^58^ | Y | N | N | PY | N | N | N | Y | PY | N | Y | Y | Y | Y | Y | N | **M** |  |
| Krishnan, 2009^59^ | Y | N | N | N | N | Y | Y | Y | Y | Y | No MA | No MA | Y | Y | No MA | N | **L** |  |
| Machado Rocha, 2008^60^ | N | N | N | PY | N | N | N | Y | PY | N | Y | Y | Y | Y | Y | N | **M** |  |
| Wang, 2008^61^ | N | N | N | N | Y | N | N | Y | PY | N | Y | Y | Y | Y | N | Y | **L** |  |
| Iskedjian, 2007^62^ | Y | N | N | N | Y | Y | N | Y | PY | N | N | Y | Y | Y | Y | N | **CL** |  |
| Mills, 2007^63^ | Y | PY | N | N | Y | Y | Y | PY | PY | N | No MA | No MA | Y | N | N | N | **L** |  |
| Shakespeare, 2003^64^ | Y | Y | N | N | Y | Y | Y | PY | N | N | No MA | No MA | Y | N | N | N | **CL** |  |
| Campbell, 2001^65^ | Y | N | N | PY | Y | Y | N | Y | PY | N | No MA | No MA | Y | N | N | Y | **M** |  |
| Huntley, 2000^66^ | N | N | N | PY | N | N | N | PY | Y | N | No MA | No MA | Y | N | N | N | **M** |  |
| Merlin, 2016^67^ | N | N | N | N | Y | Y | N | Y | N | Y | No MA | No MA | Y | N | N | Y | **CL** |  |
| Lynch, 2015^68^ | Y | N | N | PY | N | N | N | PY | PY | N | No MA | No MA | N | N | N | N | **L** |  |
| Finnerup, 2015^69^ | Y | PY | N | PY | Y | Y | N | PY | PY | N | Y | Y | Y | Y | Y | Y | **M** |  |
| Snedecor, 2013^70^ | N | N | N | PY | Y | Y | N | Y | Y | N | Y | N | N | Y | N | Y | **L** |  |
| Kuspinar, 2012^71^ | Y | N | Y | PY | Y | Y | N | Y | Y | N | N | N | Y | N | N | Y | **L** |  |
| Gloss, 2014^72^ | Y | PY | N | Y | Y | Y | Y | PY | Y | N | No MA | No MA | Y | N | N | N | **M** |  |
| Macfarlane, 2011^73^ | N | N | N | N | Y | N | N | PY | PY | N | No MA | No MA | Y | N | N | Y | **L** |  |
| Hanson, 2011^74^ | N | PY | N | N | Y | Y | N | Y | Y | N | No MA | No MA | Y | N | N | Y | **L** |  |
| Mestre, 2009^75^ | Y | PY | N | Y | Y | Y | Y | Y | Y | N | No MA | No MA | Y | Y | N | Y | **M** |  |
| Wheaton, 2009^76^ | N | N | N | N | N | N | N | N | PY | N | N | Y | N | N | N | Y | **CL** |  |
| Singh, 2007^77^ | N | N | Y | Y | N | N | N | Y | PY | N | No MA | No MA | Y | N | N | Y | **M** |  |
| Chung, 2006^78^ | Y | N | N | N | Y | Y | N | Y | Y | N | No MA | No MA | Y | NA - one study | N | N | **L** |  |
| Yavuzsen, 2005^79^ | N | N | N | N | N | N | N | PY | PY | N | No MA | No MA | N | N | N | N | **CL** |  |
| Nielsen, 2017^80^ | N | N | N | PY | Y | Y | N | Y | Y | N | No MA | No MA | Y | N | Y | Y | **M** |  |
| Pringsheim, 2012^86^ | N | N | N | N | Y | Y | N | N | Y | N | No MA | No MA | Y | N | N | Y | **L** |  |
| CADTH, 2014^81^ | Y | N | N | N | N | N | N | Y | PY | N | No MA | No MA | Y | N | N | N | **L** |  |
| SIGN, 2008^87^ | N | N | N | Y | N | N | N | N | PY | N | No MA | No MA | Y | N | N | N | **M** |  |
| Paice (American Society of Clinical Oncology), 2016^88^ | N | PY | N | N | Y | Y | N | Y | PY | Y | No MA | No MA | Y | N | N | Y | **L** |  |
| Staples, CADTH, 2018^83^ | Y | N | N | N | N | N | N | Y | PY | N | No MA | No MA | N | N | N | N | **CL** |  |
| CADTH, 2011^6^ | Y | N | N | N | N | N | N | Y | PY | N | No MA | No MA | Y | N | N | N | **L** |  |
| Verleye, The Belgian Health Care Knowledge Centre (KCE), 2012^89^ | N | N | N | PY | N | N | Y | Y | Y | Y | No MA | No MA | Y | N | N | N | **M** |  |
| NICE, 2013^90^ | Y | Y | N | PY | N | N | Y | Y | Y | N | No MA | No MA | Y | Y | No MA | N | **M** |  |
| NICE, 2014^91^ | Y | Y | Y | PY | N | N | Y | PY | Y | Y | No MA | No MA | Y | Y | N | Y | **M** |  |

*critical question; Y-yes; N-no; PY-partial yes; MA-meta-analysis; NRSI-non randomised study of interventions; M-moderate; L-low; CL-critically low; SIGN-Scottish Intercollegiate Guidelines Network; CADTH-Canadian Agency for Drugs and Technologies in Health; NICE-National Institute for Health and Care Excellence
